# Potential‐Driven Dynamic Spring‐Effect of Pd─Cu Dual‐Atoms Empowered Stability and Activity for Electrocatalytic Reduction

**DOI:** 10.1002/advs.202501393

**Published:** 2025-04-26

**Authors:** Pei‐Hua Li, Yuan‐Fan Yang, Zong‐Yin Song, Bo Liang, Yong‐Huan Zhao, Xin Cai, Zi‐Hao Liu, Jing‐Yi Lin, Meng Yang, Xiangyu Xiao, Jing Zhang, Wen‐Qing Liu, Xing‐Jiu Huang

**Affiliations:** ^1^ Institute of Environment Hefei Comprehensive National Science Center Hefei 230088 P. R. China; ^2^ Key Laboratory of Environmental Optics and Technology And Environmental Materials and Pollution Control Laboratory Institute of Solid State Physics HFIPS, Chinese Academy of Sciences Hefei 230031 P. R. China; ^3^ Key Laboratory of Organic Compound Pollution Control Engineering (MOE) School of Environmental and Chemical Engineering Shanghai University Shanghai 200444 P. R. China; ^4^ Beijing Synchrotron Radiation Facility Institute of High Energy Physics Chinese Academy of Sciences Beijing 100049 P. R. China

**Keywords:** dual‐atom catalysts, electrochemical catalysis, in situ XAFS, single‐atom catalysts, spring effect

## Abstract

Atomic‐level catalysts are extensively applied in heterogeneous catalysis fields. However, it is a general but ineluctable issue that active metal atoms may migrate, aggregate, deactivate, or leach during reaction processes, suppressing their catalytic performances. Designing superior intrinsic‐structural stability of atomic‐level catalysts with high activity and revealing their dynamic structure evolution is vital for their wide applications in complex reactions or harsh conditions. Herein, high‐stable Pd─Cu dual‐atom catalysts with PdN_3_─CuN_3_ coordination structure are engineered via strong chelation of Cu^2+^‐ions with electron pairs from palladium‐source, achieving the highest turnover frequency under the lowest overpotential for Cr(VI) electrocatalytic reduction detection in strong‐acid electrolytes. In situ X‐ray absorption fine structure spectra reveal dynamic “spring‐effect” of Cu─Pd and Cu─N bonds that are reversibly stretched with potential changes and can be recovered at 0.6 V for regeneration. The modulated electron‐orbit coupling effect of Pd─Cu pairs prevents Cu‐atoms from aggregating as metallic nanoparticles. Pd─Cu dual‐atoms interact with two O atoms of H_2_CrO_4_, forming stable bridge configurations and transferring electrons to promote Cr─O bond dissociation, which prominently decreases reaction energy barriers. This work provides a feasible route to boost the stability and robustness of metal single‐atoms that are easily affected by reaction conditions for sustainable catalytic applications.

## Graphical abstract

The dynamic “spring‐like” behavior of Pd─Cu dual atoms with PdN_3_─CuN_3_ coordination structure can be stretched and recovered with potential variation, which remarkably elevates the structural stability and catalytic activity of Cu atoms for electrocatalytic reduction reactions in strong‐acid electrolytes. The modulated electron‐orbit coupling effect and potential‐driven dynamic spring behavior of inter‐metals effectively prevent metallic single‐atoms from aggregating as nanoparticles.

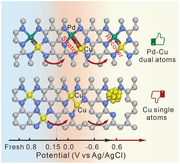

## 1 Introduction

Single‐atom catalysts (SACs) relate to active metal atoms chemically bonded with other elements and dispersed on the surface of support materials.^[^ ^1^ ^]^ SACs possess the unique properties of high catalytic activity, selectivity, optimal atomic utilization, and desirable controllability,^[^ ^2^ ^]^ and show great application potential in energy conversion,^[^ ^3^ ^]^ photocatalytic systems,^[^ ^4^ ^]^ pollutant degradation,^[^ ^5^ ^]^ batteries,^[^ ^6^ ^]^ etc. Currently, massive efforts have been made to further explore and optimize the catalytic performance of SACs,^[^ ^7^ ^]^ but some obstacles remain to retard their actual application in various scientific and industrial fields.^[^ ^8^ ^]^

Specifically, SACs usually have a relatively small active domain or even possess only one active site,^[^ ^9^ ^]^ which always generates onefold adsorption mode and fixed catalytic behavior toward targets, or even are inapplicable in multi‐step reactions with complex intermediates.^[^ ^10^ ^]^ Importantly, there is an inevitable contradiction between the high metallic load and the independence of single atoms under synthetic conditions.^[^ ^11^ ^]^ Besides, although stable SACs are successfully synthesized, it is still difficult to effectively avert the possible migration and inactivation of single atoms under reaction conditions, especially in complex multi‐step reactions or harsh environments.^[^ ^12^ ^]^ Recently, the instability and migration problems of SACs under operating conditions have been reported successively,^[^ ^13^ ^]^ typically transition metal Cu single‐atoms with easily variable valence. For example, Wang et al. designed copper single‐atoms on CeO_2_ substrate and elucidated the reconstitution process of Cu single atoms to Cu_4_ clusters during electrocatalytic urea synthesis.^[^ ^14^ ^]^ Yang et al. revealed that with the voltage changed from 0.0 to −1.0 V (vs reversible hydrogen electrode (RHE)), Cu single‐atoms on nitrogen‐carbon restructured to nanoparticles (≈5 nm) during electrochemical reduction of nitrate to ammonia, triggering concurrently Cu^2+^ reduced to Cu^+^ and Cu^0^.^[^ ^15^ ^]^ Yao et al. identified that Cu single‐atoms embedded on Au nanoparticles can migrate from the vertex position to the (100) plane under reduction potential conditions, markedly modulating the electronic structure of Au substrates.^[^ ^16^ ^]^ In essence, the unsaturated coordination structure and high activity of single‐atoms are frailly affected by reaction conditions, such as voltages, light, temperature, electrolyte, atmosphere, etc.^[^ ^17^ ^]^ then possibly occurring dynamic migration, reconstitution, or aggregation of active sites, thereby restraining their catalytic activity. Therefore, enhancing the intrinsic structural stability of Cu atomic‐level catalysts with high activity under operating conditions and exploring structural evolution simultaneously is of paramount imperative to facilitate wide applications and summarize the guiding strategies.

How to maintain the independence, activity, and stability of atomic‐level active sites during reaction processes has always been a thorny problem. Many researchers have sought various approaches to improve the stability of atomic‐level active sites, such as regulating ligand and number, optimizing supports, controlling synthesis conditions, alloying, etc.^[^ ^18^ ^]^ Beyond that, the introduction of heterogenic metal atoms to construct dual‐metallic atom catalysts (DACs) is considered one of the effective strategies to simultaneously stabilize single atoms, enhance activity, and increase active sites.^[^ ^19^ ^]^ Especially, the close coupling of two heterogenic metal atoms with different electronegativities could break charge symmetric distribution and motivate charge rearrangement,^[^ ^20^ ^]^ modulating local electronic structures to stabilize metallic atoms and strengthen their interaction with supports.^[^ ^21^ ^]^ Such tight interaction of two hetero‐metallic atoms with coordinated atoms contributes to more stable structure units to retain the independence and high activity of metallic atoms. Moreover, the highly controllable ability and synergistic catalytic effect of DACs enable optimization and customization of catalytic, selective, adsorptive, or desorption performances to satisfy specific reaction paths by regulating the type, proportion, coordination relation, and spatial position of two metallic atoms.^[^ ^22^ ^]^ Thus, designing heterogenic DACs with high activity is an inspiring pathway to boost the structural stability and catalytic capacity of metallic Cu‐atoms that are reported easily affected by experimental conditions.^[^ ^23^ ^]^ Although bimetallic sites of Co─Cu, Fe─Cu, Ag─Cu, etc. have been reported to significantly enhance catalytic performance,^[^ ^24^ ^]^ most of these bimetallic sites are randomly mixed and anchored on the support materials without a specific intermetallic coordination interaction. The preparation of precise and uniform coordination structures of dual‐metallic sites is still a technical difficulty. Furthermore, the exploration of the structural dynamic evolution and synergistic mechanism of DACs is crucial to recognizing true active structures and summarizing effective guiding strategies, which is often overlooked. As an outstanding hydrogenation metal, Pd single‐atoms possess variable valence states and controllable coordination structures^[^ ^25^ ^]^ and could be combined with Cu atoms generating unexpected synergistic effects in catalysis fields.^[^ ^26^ ^]^ Therefore, constructing high‐active and stable Pd─Cu dual‐atom catalysts with precise intermetallic coordination and exploring their structural dynamic evolution have seminal research significance to expand the application of atomic‐level catalysts in various fields.

In addition, a suitable research system is needed to explore the dynamic activity and stability of Pd─Cu dual‐atoms during reaction processes. A typical persistent hypertoxic pollutant of hexavalent chromium ion (Cr(VI)) is easily dissolved in water resources and bonded with oxyanion, exhibiting grievous teratogenic‐mutagenic properties in ecological environments.^[^ ^27^ ^]^ The electrochemical method is an efficient strategy to promote Cr(VI) transforming to nontoxic trivalent chromium ions (Cr(III)) and also is a rapid determination technique to on‐site evaluate Cr(VI) pollution.^[^ ^28^ ^]^ Notably, electrocatalytic reduction of Cr(VI) is mainly operated in strong acid electrolytes due to seven H^+^ ions demanded to participate in reduction reactions, which require high acid resistance and stability of catalysts.^[^ ^29^ ^]^ Besides, it is difficult to rapidly and effectively destroy several Cr─O bonds to reduce Cr(VI) to Cr(III), which often relies on precious metal‐based catalysts with high activity. Such harsh reaction conditions and low efficiency of Cr(VI) reduction have a high demand for excellent stability and activity of catalysts. Therefore, the electrocatalytic reduction of Cr(VI) is chosen as an ideal model system to explore the dynamic stability and activity of Pd─Cu dual‐atom catalysts during reaction processes.

In this work, Pd─Cu dual‐metal pairs, Pd single‐atoms, and Cu single‐atoms were designed on nitrogen‐doped carbon materials (N─C) for electrocatalytic reduction of Cr(VI), marked as Pd─Cu DAC, Pd SAC, and Cu SAC, respectively. Pd─Cu DAC exhibits remarkable activity and stability for Cr(VI) reductions, achieving the highest turnover frequency and the lowest overpotential. In situ X‐ray absorption fine structure (XAFS) spectra prove the dynamic “spring‐effect” of Cu─Pd and Cu─N bonds that reversibly stretched with reduction reactions of Cr(VI) driven by potentials, then recovered and regenerated under the potential of 0.6 V. Such a reversible chemical environment and coordination structure of Pd─Cu DAC ensures its high catalytic activity and structural stability. But if there is no coupling effect of Pd atoms, isolated Cu single‐atoms are inclined to aggregate forming dimers or metallic nanoparticles, which can not be regenerated and seriously refrain from their activity. A stable bridge interaction configuration of Pd─Cu atomic pairs with H_2_CrO_4_ and the corresponding catalytic mechanisms are demonstrated deeply.

## 2 Results and Discussion

### 2.1 Morphology and Structural Characterizations of Pd─Cu DAC, Pd SAC, and Cu SAC Catalysts

**Figure 1:**
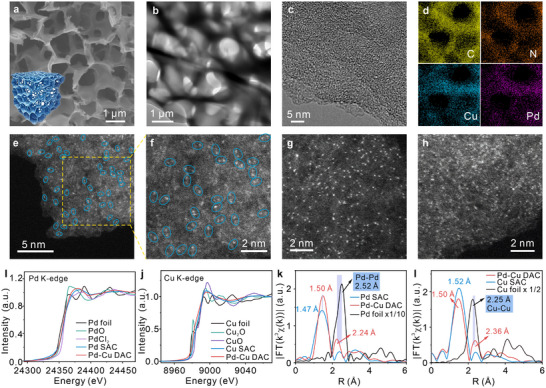
Morphology and structural characterizations of Pd─Cu DAC, Pd SAC, and Cu SAC catalysts. a) SEM, b) TEM, and c) HR‐TEM images of Pd─Cu DAC. The inset of Figure 1a shows a structural schematic diagram of Pd─Cu DAC. d) Elemental mapping images of C, N, Pd, and Cu elements in Pd─Cu DAC. Aberration‐corrected HAADF‐STEM images of e,f) Pd─Cu DAC, g) Pd SAC, and h) Cu SAC catalysts. The white spots denote metallic Pd or Cu atoms. i) Comparison in the normalized Pd K‐edge XANES spectra of Pd─Cu DAC and Pd SAC catalysts. j) Comparison in the normalized Cu K‐edge XANES spectra of Pd─Cu DAC and Cu SAC catalysts. k) Comparison in the Fourier transformed Pd K‐edge EXAFS spectra (R space, k^3^‐weighted) between Pd─Cu DAC and Pd SAC catalysts without correcting for scattering phase shift. l) Comparison in the Fourier transformed Cu K‐edge EXAFS spectra (R space, k^3^‐weighted) between Pd─Cu DAC and Cu SAC catalysts without correcting for scattering phase shift.

The strong chelation between free Cu^2+^ ions and the lone pair electron of NH_3_ from PdN_4_H_12_Cl_2_·H_2_O was utilized to construct fixed‐adjacent Pd─Cu dual‐atoms with a uniform structure and synthesized Pd single‐atoms and Cu single‐atoms catalysts for comparisons.^[^ ^30^ ^]^ From the scanning electron microscopy (SEM), and transmission electron microscopy (TEM) images in **Figure** **1**a,b, it is observed that Pd─Cu DAC exhibits a 3D porous cross‐linked structure, and its structural schematic diagram is displayed in the inset of Figure 1a. As shown in the high‐resolution TEM (HR‐TEM) image (Figure 1c), no small nanoparticles or lattice fringes can be found, which suggests that metal atoms do not agglomerate and Pd─Cu DAC possesses the amorphous state. Besides, elemental mapping images in Figure 1d reflect the homogeneous dispersion of C, N, Pd, and Cu elements on Pd─Cu DAC. Moreover, the high‐angle annular dark‐field scanning transmission electron microscopy (HAADF‐STEM) images (Figure 1e,f) display a lot of slight white spots and brighter spots with good dispersibility, which always appear in pairs as shown in blue ellipse lines.^[^ ^31^ ^]^ The slight white spots represent Cu atoms, and brighter spots signify Pd atoms due to the larger atomic number of Pd than Cu atoms. These illustrate that most of the Pd and Cu atoms show in pairs on the substrate. In addition, Pd SAC and Cu SAC have a similar morphology and structure to Pd─Cu DAC, as displayed in Figures S1 and S2 (Supporting Information). Only atomically dispersed bright spots of Pd atoms emerge on the substrate of Pd SAC (Figure 1g; Figure S1e,f, Supporting Information), and numerous isolated‐distributed bright spots of Cu atoms arise on Cu SAC catalyst (Figure 1h; Figure S2e–i, Supporting Information).

Furthermore, X‐ray diffraction (XRD) patterns and Raman spectra in Figure S3a,b (Supporting Information) confirm that their substrates all possess graphene‐like structures with similar defect contents. X‐ray photoelectron spectroscopy (XPS) in Figure S3c (Supporting Information) and energy dispersive spectroscopy (EDS) in Figure S3d–f (Supporting Information) demonstrate that only C, N, Pd, and/or Cu elements exist in these three catalysts without other impure elements. Besides, the specific surface areas of Pd─Cu DAC, Pd SAC, and Cu SAC are measured as 275, 363, and 223 m^2^ g^−1^, respectively, with similar average pore diameters (Figure S4, Supporting Information). Moreover, XAFS spectroscopy was employed to analyze elemental chemical states and coordination structures. As observed in normalized Pd K‐edge X‐ray absorption near edge structure (XANES) spectra (Figure 1i), it can be found that the pre‐edge tendencies of Pd K‐edge in Pd─Cu DAC position slightly left than that of Pd SAC, and both of them are close to PdO, which suggests that a relatively lower average valence state of Pd atom in Pd─Cu DAC than that in Pd SAC, although they are likely close to +2. The incorporation of the Cu atom slightly reduced the valence state of Pd atoms, which is also validated by the characteristic peak shift in high‐resolution X‐ray photoelectron spectroscopy (HR‐XPS) of Pd 3d, compared in Figure S5c (Supporting Information). As depicted in normalized XANES spectra of Cu K‐edge (Figure 1j), the pre‐edge trend of Cu K‐edge XANES spectra in Pd─Cu DAC moves to a lower energy than that in Cu SAC, and both of them locate between these in CuO and Cu_2_O. These imply that chemical valences of Cu atoms both in Pd─Cu DAC and Cu SAC are between +1 and +2,^[^ ^26^ ^]^ and the entrance of Pd atoms enables the decreased valence state of Cu atoms in Pd─Cu DAC,^[^ ^32^ ^]^ which is also certified by the increased relative content of Cu^+^ and decreased relative content of Cu^2+^ in Pd─Cu DAC compared with Cu SAC, as shown in HR‐XPS spectra of Cu 2p (Figure S5d, Supporting Information). Besides, the HR‐XPS spectra of N 1s in Figure S5b (Supporting Information) reveal that the introduction of Pd or Cu atoms renders differences in the N chemical states. Finally, the mass percents of Pd and Cu atoms in Pd─Cu DAC, Pd SAC, and Cu SAC are assayed as 3.0 wt.% (Pd)/3.2 wt.% (Cu), 4.5 wt.%, and 5.0 wt.% via inductively coupled plasma‐atomic emission spectrometry (ICP‐AES), respectively.

**Figure 2:**
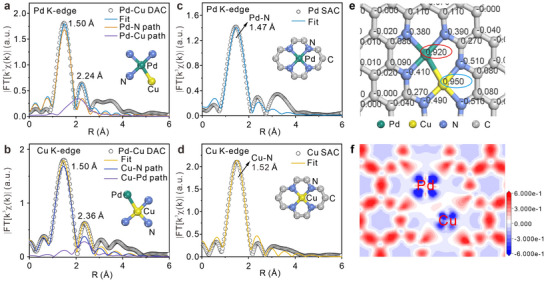
Revelation of coordination environment and electronic structure of Pd─Cu DAC. Pd K‐edge EXAFS spectra (R space, k^3^‐weighted) of a) Pd─Cu DAC and c) Pd SAC after Fourier transform and their corresponding fitting curves. Cu K‐edge EXAFS spectra (R space, k^3^‐weighted) of b) Pd─Cu DAC and d) Cu SAC catalysts after Fourier transform and their corresponding fitting curves. The insets show their structure schematic of coordination models. e) Coordination structure model of Pd─Cu DAC optimized by DFT calculation and its Mulliken charge distribution. f) Charge density difference plot of the horizontal slice through the Pd, Cu, N, and C atomic layer on Pd─Cu DAC. The blue area represents electron depletion, and the red area represents electron accumulation.

Extended X‐ray absorption fine structure (EXAFS) spectra of Pd K‐edge and Cu K‐edge are compared in Figure 1k,l after Fourier transform. Two main scattering signals of Pd─Cu DAC are present at 1.50 and 2.24 Å (Figure 1k), much differing from the Pd─Pd scattering signal (2.52 Å) of Pd foil. It verifies that there are no Pd─Pd metallic bonds in Pd─Cu DAC.^[^ ^33^ ^]^ The main scattering signal of Pd SAC arises at 1.47 Å, and no Pd─Pd scattering signals emerge at 2.52 Å, which proves that the Pd elements of Pd SAC are fully dispersed as Pd single‐atoms. For Cu K‐edge EXAFS spectra in Figure 1l, two main scattering signals of Pd─Cu DAC appear at 1.50 and 2.36 Å, and Cu─Cu scattering signals of Cu foil arise at 2.25 Å, which demonstrates that there are no Cu─Cu metallic bonds in Pd─Cu DAC. In the Cu SAC catalyst, only one main scattering signal is located at 1.52 Å, significantly differing from Cu─Cu scattering signals, which unravels that the Cu element of Cu SAC is totally distributed as Cu single atoms on the substrate. To further research the structure information of Pd and Cu atoms, the above EXAFS spectra are analyzed in **Figure** **2**, and the fitting results are displayed in Tables S1 and S2 (Supporting Information). As depicted in Figure 2a, there are Pd─N and Pd─Cu scattering paths existed in the first and second coordination shells of Pd atoms in Pd─Cu DAC with the coordination numbers of 2.9 and 0.7 and the scattering distances of 2.02 and 2.44 Å, respectively.^[^ ^34^ ^]^ Besides, it is discovered from the fitting results in Figure 2b and Table S2 (Supporting Information) that the Cu atom of Pd─Cu DAC is averagely coordinated with 2.8 N atoms and 0.6 Pd atom with scattering distances of 1.95 (Cu─N) and 2.53 Å (Cu─Pd). The existence of Pd─Cu and Cu─Pd scattering paths in the Pd and Cu K‐edge EXAFS spectra implies the formation of Pd─Cu metallic bonds in Pd─Cu DAC, and Pd─Cu atoms mostly appear in pairs as observed in Figure 1e,f. Meanwhile, both individual Pd and Cu atoms are also coordinated with ≈3 N atoms from the substrate. In contrast, a Pd atom of Pd SAC is averagely coordinated with 3.9 N atoms with a Pd─N bond length of 2.00 Å (Figure 2c; Table S1, Supporting Information).^[^ ^35^ ^]^ In addition, the Cu atom of Cu SAC is averagely coordinated with 3.7 N atoms with a bond length of 1.94 Å (Figure 2d; Table S2, Supporting Information).^[^ ^36^ ^]^ Notably, the small peak at ≈2.0–2.5 Å in the EXAFS spectra of Pd SAC and Cu SAC (Figure 2c,d) likely originates from the single‐scattering or multiple‐scattering effect involving C atom in the second‐shell coordination from the support.^[^ ^37^ ^]^ Moreover, to further rule out the contribution of Pd─Pd or Cu─Cu metallic bonds to the signal peak at ≈2.0–2.5 Å observed in Pd SAC and Cu SAC, their EXAFS data were analyzed via Wavelet transform to resolve distinct k‐ and R‐space signatures. Figure S6a,b (Supporting Information) shows the Wavelet transformed EXAFS (WT‐EXAFS) plot of Pd K‐edge in Pd SAC and Pd foil, respectively. It can be clearly observed that the absence of the signal intensity in high‐k or long‐R region in Pd SAC further confirms the atomic‐level dispersion of Pd metal sites, while Pd‐metal foil exhibits obvious characteristic Pd─Pd metallic bonding signals at high‐k and long‐R region.^[^ ^38^ ^]^ Such similar phenomena are also found in WT‐EXAFS spectra of Cu K‐edge in Cu SAC and Cu foil (Figure S6c,d, Supporting Information), further demonstrating the atomic‐level dispersion of Cu metal sites. The fitted k^3^χ(k) oscillations corresponding to Figure 2a–d coincide nicely with raw data (Figure S7, Supporting Information), affirming the veracity of these fitting results. Therefore, three atomic‐level catalysts of Pd─Cu atom pairs, Pd single atoms, and Cu single atoms with specific coordination structures are elaborately synthesized for later experiments, respectively.

### 2.2 Differences in Electronic Structures of Pd─Cu DAC, Pd SAC, and Cu SAC

Electronic structure differences of Pd─Cu DAC, Pd SAC, and Cu SAC are explored. Based on the atomic structure data of these three catalysts obtained from the above experiments, their corresponding atomic structure models were optimized by density functional theory (DFT) calculation. As shown in Figure S8a (Supporting Information), both Pd and Cu atoms in Pd─Cu DAC are coordinated with 3 N atoms, respectively, meanwhile forming Pd─Cu metallic bonds, and the atomic structure is marked as PdN_3_‐CuN_3_. For Pd SAC and Cu SAC catalysts, Pd and Cu atoms are coordinated with 4 N atoms, respectively (Figure S8b,c, Supporting Information), and their structures are labeled as PdN_4_ and CuN_4_. The formation energies of Pd─Cu DAC, Pd SAC, and Cu SAC are calculated as −9.69, −6.07, and −5.28 eV, respectively. The lowest formation energy of Pd─Cu DAC demonstrates that Pd─Cu diatomic sites are the most easily generated on the N─C substrate and exhibit the most stable structure. Besides, Mulliken charge distribution unravels that the coupling of Pd and Cu atoms in Pd─Cu DAC (Figure 2e) renders their charge decreasing, compared to that in Pd SAC or Cu SAC (Figure S9, Supporting Information). The charge density difference plots demonstrate that Pd─Cu bonds in Pd─Cu DAC (Figure 2f) are surrounded by the red area, implying the electron accumulation of Pd─Cu bonds, and the red area intensity of N atoms around Pd─Cu dual‐atoms is much lighter than that around Pd or Cu single‐atoms (Figure S10, Supporting Information), illustrating that Pd─Cu diatomic pairs transfer less electrons to the N─C substrate and form electron‐rich centers. In addition, the partial density of states (PDOS) of Pd and Cu atoms are calculated and compared in Figure S11 (Supporting Information). The *s*‐, *p*‐, and *d*‐orbital electrons of Pd atoms in Pd─Cu DAC shift toward lower energy levels, compared to those in Pd SAC, certifying that their coupling effect with Cu atoms enables the system more stable. Moreover, by comparing the Cu PDOS diagrams in Pd─Cu DAC and Cu SAC, it is found that new *p*‐ and *d*‐orbital electrons of Cu atoms in Pd─Cu DAC generated at ca. −1.9 and 0.4 eV, which coincide well with the *d*‐orbital electrons of Pd atoms in Pd─Cu DAC, indicating the strong coupling interaction between Pd and Cu atoms. Moreover, the *d*‐band centers of Pd and Cu atoms in Pd─Cu DAC decrease to −3.55 and −3.60 eV from −3.17 (in Pd SAC) and −3.50 eV (in Cu SAC), respectively. The mutually modulated electron‐orbit coupling effect of Pd and Cu atoms and negative shifts of *d*‐band centers are exceptionally favorable for the adsorption and catalysis behaviors toward Cr(VI).

### 2.3 Electrochemical Performance for the Catalytic Reduction of Cr(VI)

**Figure 3:**
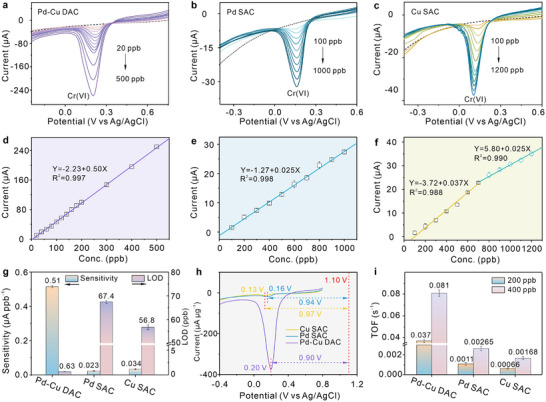
Excellent electrocatalysis reduction performance of Pd─Cu DAC for Cr(VI). LSV reduction signals of a) Pd─Cu DAC, b) Pd SAC, and c) Cu SAC modified electrodes with the increased concentration of Cr(VI). The corrected linear relationship plots of current values versus Cr(VI) concentrations of d) Pd─Cu DAC, e) Pd SAC, and f) Cu SAC. g) Comparison in response sensitivity and the theoretical limit of detection of Pd─Cu DAC, Pd SAC, and Cu SAC (*n* = 3). h) Comparison in reduction signals of 200 ppb Cr(VI) catalyzed by metal sites per microgram of Pd─Cu DAC, Pd SAC, Cu SAC, and their reduction overpotentials toward Cr(VI). i) Comparison in turnover frequencies of Pd─Cu DAC, Pd SAC, and Cu SAC for the catalytic reduction of 200 and 400 ppb Cr(VI) (*n* = 3).

The prepared Pd─Cu DAC, Pd SAC, and Cu SAC catalysts are applied to modify screen‐printed carbon electrodes (SPCEs) for catalyzing Cr(VI) reduction reactions. The experimental results of electrochemical impedance spectroscopy (EIS) and cyclic voltammetry (CV) certify that more exceptional activity and faster electronic transfer efficiency of Pd─Cu DAC than that of Pd SAC and Cu SAC (Figure S12, Supporting Information). **Figure** **3** displays the electrocatalytic reduction signals of these catalysts toward Cr(VI) measured by linear sweep voltammetry (LSV) in 0.5 m H_2_SO_4_ electrolytes. Pd─Cu DAC exhibits a low reaction limit of 20 ppb Cr(VI) (Figure 3a), and the generated current signals appear at 0.20 V (vs Ag/AgCl), then gradually elevating with increased concentrations of Cr(VI). However, both Pd SAC (Figure 3b) and Cu SAC (Figure 3c) can just catalyze Cr(VI) reduction reactions with a concentration limit of 100 ppb. Notably, when Cr(VI) concentration exceeds 700 ppb, the increase rate of peak currents and reaction efficiency of Cu SAC become slow. Moreover, as displayed in Figure 3d–f, their peak currents almost increase linearly as Cr(VI) concentrations, the slopes of their linear equations signify the response sensitivity. To ensure the stability and reliability of the experimental results, three independent repeat tests (*n* = 3) under the same condition were conducted to obtain the error bars of test data. The response sensitivity and theoretical limitations of detection (LOD) are displayed in Figure 3g. The sensitivity of Pd─Cu DAC, Pd SAC, and Cu SAC toward Cr(VI) are 0.51±0.01, 0.023±0.003, and 0.034±0.004 (or 0.025±0.002) µA ppb^−1^ (*n* = 3), respectively. Cu SAC electrodes show distinct electrocatalytic behavior at high and low concentrations of Cr(VI), which may be ascribed to saturation adsorption of active sites or the changes in active structure. Besides, their theoretical LOD values of Pd─Cu DAC, Pd SAC, and Cu SAC were calculated as 0.63±0.04, 67.4±0.57, and 56.8±1.06 ppb (3σ method, *n* = 3), respectively. Surprisedly, the Pd─Cu DAC presents the highest response sensitivity for Cr(VI) compared to other noble‐metal nanomaterials currently reported (Table S3, Supporting Information), which is also 22‐fold and 15‐fold higher than that of Pd SAC and Cu SAC. In addition, both the actual LOD (20 ppb) and theoretical LOD (0.63 ppb) of Pd─Cu DAC are far below the permissible limit (50 ppb) in drinking water prescribed by the World Health Organization, unraveling a great practical application potential of Pd─Cu DAC. Furthermore, to figure out the catalytic ability of these catalysts, the normalized LSV signals of 200 ppb Cr(VI) catalyzed by metal sites per microgram of Pd─Cu DAC, Pd SAC, and Cu SAC are compared in Figure 3h. The normalized reduction signal of Pd─Cu diatomic pairs per microgram is over 30 times higher than Pd or Cu single‐atoms. Furthermore, their reduction currents of Cr(VI) are located at 0.2, 0.16, and 0.13 V, respectively, while the standard reduction potential of Cr(VI) transforming to Cr(III) is calculated as 1.10 V (vs Ag/AgCl), implying that the reduction reaction overpotential of Cr(VI)/Cr(III) on Pd─Cu DAC (0.90 V) is lower than that on Pd SAC (0.94 V) and Cu SAC (0.97 V). These further demonstrate the excellent catalytic ability and high reaction efficiency of Pd─Cu DAC to accelerate Cr(VI) reducing to Cr(III). In addition, turnover frequencies (TOF) of Pd─Cu DAC, Pd SAC, and Cu SAC for the reduction reactions of 200 and 400 ppb Cr(VI) are achieved via calculating the integral areas of current signals with reaction times (Figure S13, Supporting Information). The detailed calculation method of TOF value is presented in Supporting Information. The obtained data and error bars displayed in Figure 3i were calculated based on three independent repeat experiments (*n* = 3). The TOF values of Pd─Cu DAC, Pd SAC, and Cu SAC toward 200 ppb Cr(VI) are 0.037±0.002, 1.1×10^−3^±1.4×10^−4^, 6.6×10^−4^±6.4×10^−5^ s^−1^, respectively, and their TOF values toward 400 ppb Cr(VI) are 0.081±0.003, 2.65×10^−3^±2.1×10^−4^, 1.68×10^−3^±1.77×10^−4^ s^−1^, respectively. Consequently, the TOF value of Pd─Cu DAC is over 30‐fold higher than that of Pd SAC and over 50 times higher than that of Cu SAC, revealing the superior catalytic activity and reduction efficiency of Pd─Cu DAC toward Cr(VI). Therefore, it is affirmed that the synergistic catalytic effect of Pd─Cu dual‐atoms prominently enhances outstanding electrochemical performances of Pd─Cu DAC, which exhibits superhigh catalytic ability for Cr(VI) reduction reactions.

The electrochemical stability of catalysts is of paramount significance for reliable catalytic performance and accurate detection results. The electrochemical stability test of Pd─Cu DAC, Pd SAC, and Cu SAC catalysts under electroreduction conditions has been operated, and the structural characterizations of the catalyst after electrochemical tests also have been supplemented. The corresponding results are shown in Figures S14 and S31–S33 (Supporting Information), respectively. As depicted in Figure S14a (Supporting Information) and the inset, during 10‐times cyclic electroreduction tests, the reduction current signals of Cr(VI) obtained by Pd─Cu DAC almost have no variation in peak value, peak shape, and potential position. The relative standard deviation (RSD) of these peak current values was calculated as 2.68%, proving the glorious electrochemical stability of Pd─Cu DAC under electroreduction conditions. Similarly, Pd SAC also exhibits excellent stability with consistent current signals and a small peak current RSD of 3.00% during 10 times of repetitive tests (Figure S14b, Supporting Information). In contrast, the reduction current signals of Cr(VI) achieved by Cu SAC begin to widen after 7th‐cycle tests, and these peak currents show a downward trend with cycle number increased with a large RSD of 7.21%. The large RSD value, decreased peak current, and widened peak shape demonstrate the inferior stability of Cu SAC. Pd─Cu DAC and Pd SAC exhibit excellent electrochemical stability to catalyze the electroreduction of Cr(VI), while Cu SAC possesses inferior stability during electroreduction conditions. Thus, the participation of Pd atoms extraordinarily elevates the overall structural stability of Cu atoms in Pd─Cu DAC under electrochemical conditions.

### 2.4 Variation in Atomic Structures and Chemical Environments of Pd─Cu DAC, Pd SAC, and Cu DAC After Interacting with Cr(VI)

**Figure 4:**
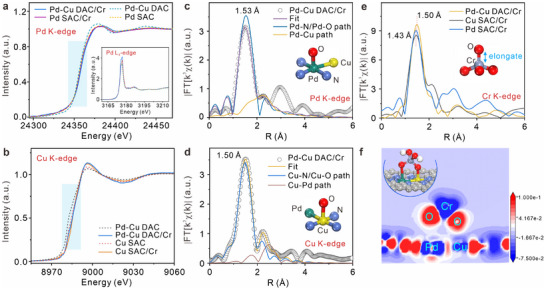
Interaction mechanism of Pd─Cu DAC with Cr(VI). a) Comparison in normalized Pd K‐edge and L_3_‐edge XANES (inset) spectra of Pd─Cu DAC and Pd SAC before and after interacting with Cr(VI). b) Comparison in normalized Cu K‐edge XANES spectra of Pd─Cu DAC and Cu SAC before and after interacting Cr(VI). c) Pd K‐edge and d) Cu K‐edge EXAFS spectra (R space, k^3^‐weighted) in Pd─Cu DAC after Fourier transform without correcting for scattering phase shift and their fitting curves. Insets show the coordination structure schematics of Pd and Cu atoms in Pd─Cu DAC. e) Comparison in Cr K‐edge EXAFS spectra (R space, k^2^‐weighted) in Pd─Cu DAC/Cr, Pd SAC/Cr, and Cu SAC/Cr samples after Fourier transform without correcting for scattering phase shift. Inset displays the coordination structure schematics of center Cr atoms. f) Charge density difference plot of the vertical slice through the Pd, Cu, Cr, and O atomic layer on Pd─Cu DAC/Cr. The blue area represents electron depletion, and the red area represents electron accumulation. The inset shows the bridge interaction configuration of Pd─Cu DAC with Cr(VI).

Furthermore, the variation in atomic structures and chemical environments of three catalysts before and after interacting with Cr(VI) are characterized via XAFS and XPS technologies, and the results are displayed in **Figure** **4**, Figures S15 and S16 (Supporting Information). The HR‐XPS spectra of Cr 2p_1/2_ and Cr 2p_3/2_ in Pd SAC/Cr and Cu SAC/Cr (Figure S15b, Supporting Information) can be deconvoluted into four characteristic peaks located at 577.1, 579.1, 586.6, and 588.4 eV, assigned to Cr^6+^ and Cr^3+^—Cr^6+^, respectively, which indicates that part of adsorbed Cr(VI) ions are reduced on Pd SAC and Cu SAC. The relative proportions of the reduced chromium ions are 75.5% and 62.0% on Pd SAC and Cu SAC, respectively. Similarly, four characteristic peaks of HR‐XPS Cr 2p spectra in Pd─Cu DAC/Cr arise at 577.1, 578.4, 586.6, and 587.8 eV. Their binding energies are all lower than those of Cr^6+^, which proves that all the adsorbed chromium ions were reduced by Pd─Cu DAC transforming to Cr^3+^—Cr^6+^. Pd─Cu DAC exhibits a more glorious catalytic ability to reduce Cr(VI) than Pd SAC and Cu SAC. Moreover, the left shift of Pd 3d HR‐XPS spectra (Figure S15c, Supporting Information) and the increased relative content of Cu^2+^ in Cu 2p HR‐XPS spectra (Figure S15d, Supporting Information) reflect that the electron loss phenomena of Pd─Cu dual‐atoms after Pd─Cu DAC interacting with Cr(VI). The increased chemical state of Pd─Cu dual‐atoms and the reduced chromium ions confirm that the electrons of Pd─Cu diatomic pairs transfer to chromium ions, catalyzing their reduction reactions. The slightly elevated surface chemical states of Pd single‐atoms and Cu single‐atoms are observed after Pd SAC and Cu SAC interacted with Cr(VI) (Figure S16a,b, Supporting Information), proving their weak interaction. These further certify the Pd─Cu diatomic pairs of Pd─Cu DAC served as two active centers to catalyze efficiently Cr(VI) reductions.

In addition, the white‐line peak of Pd K‐edge XANES spectra in Pd─Cu DAC/Cr shifts toward higher energy (Figure 4a), compared to that in Pd─Cu DAC, implying that Pd atoms in Pd─Cu DAC lose electrons and their average valent state is increased after Cr(VI) adsorption. Similarly, the increased average valence state of Cu atoms in Pd─Cu DAC/Cr is also clearly unraveled in Figure 4b. These indicate that the Pd─Cu diatomic pairs as electron‐enriched centers transfer electrons to Cr(VI). In contrast, the average valence state of Pd single‐atoms in Pd SAC almost has no difference after adsorbing Cr(VI) (Figure 4a). Besides, the Pd L_3_‐edge XANES spectra (inset of Figure 4a) show that the intensity of the white line peak of Pd─Cu DAC/Cr is noticeably enhanced compared to that of Pd─Cu DAC, while there is almost no variation in the peak intensity between Pd SAC and Pd SAC/Cr. These further ascertain the elevated valence of Pd atoms in Pd─Cu DAC and the unchanged average valence of Pd atoms in Pd SAC, which coincide with the analysis results of XAFS spectra in Figure 4a. Furthermore, the white line peak of Cu K‐edge XANES spectra in Cu SAC/Cr (Figure 4b) slightly moves to higher energy after adsorbing Cr(VI), suggesting the mildly increased valence of Cu single‐atoms.

Moreover, the atomic and geometric structures of Pd and Cu atoms are obtained by fitting their Fourier‐transformed EXAFS spectra (Figure 4c,d; Figure S17, Supporting Information). The main characteristic peaks of Pd K‐edge EXAFS spectrum in Pd─Cu DAC/Cr can be identified as two scattering signals of Pd─N/Pd─O (2.10 Å) and Pd─Cu paths (2.45 Å) with the coordination number of 4.2 and 0.8, respectively (Figure 4c; Table S1, Supporting Information). The increased coordination number of Pd─N/Pd─O path (4.2 vs 2.9) can be attributed to the contribution of Pd atoms adsorbing O atoms from Cr(VI), because Cr(VI) ions mainly exist in the form of H_2_CrO_4_ in the condition of pH < 1.^[^ ^39^ ^]^ It reveals the formation of Pd─O bonds with a coordination number of ≈1.3. Besides, the length of Pd─N bonds is stretched to 2.10 Å. It is proved that the Pd atom in Pd─Cu diatomic pair is one of the active centers and averagely interacted with one O atom of H_2_CrO_4_. In addition, the main signals of Cu K‐edge EXAFS spectra in Pd─Cu DAC/Cr are recognized as two scattering paths of Cu─N/Cu─O and Cu─Pd (Figure 4d). The increased coordination number (4.0 vs 2.8) of Cu─N/Cu─O path is also ascribed to the contribution of Cu atoms adsorbing O atoms from H_2_CrO_4_ (Table S2, Supporting Information). The formation of Cu─O bonds with a coordination number of 1.2 demonstrates that Cu atoms of Pd─Cu diatomic pairs serve as active sites for adsorbing and interacting with H_2_CrO_4_. Thus, both Pd and Cu atoms in Pd─Cu DAC are active centers for capturing and catalyzing H_2_CrO_4_ molecules. In contrast, as depicted in Figure S17, Tables S1 and S2 (Supporting Information), there is almost no obvious difference in the atomic structures of Pd atoms in Pd SAC and Cu atoms in Cu SAC before and after Cr(VI) adsorption. Their corresponding k^3^χ(k) oscillation curves in Figures S18 and S19 (Supporting Information) reflect a good match between the original data and fitting curves, implying the accuracy and reliability of these analyses. Therefore, it is demonstrated that Pd─Cu diatomic pairs are inclined to capture and interact with H_2_CrO_4_ by forming Pd─O and Cu─O bonds, but the isolate Pd and Cu single‐atoms can not effectively adsorb and interact with H_2_CrO_4_.

In addition, the changes in the geometric structure and chemical state of H_2_CrO_4_ before and after interacting with these three catalysts were explored. Their normalized Cr K‐edge XANES and EXAFS spectra are compared in Figure 4e and Figure S20 (Supporting Information). Noticeably, the pre‐edge trend of Cr K‐edge in Pd─Cu DAC/Cr moves left to lower energy, compared with that in Pd SAC/Cr, and Cu SAC/Cr, which indicates that the adsorbed‐Cr ions on Pd─Cu DAC present the lowest valent state and obtain more electrons during the interaction process. Figure 4e displays the Cr K‐edge EXAFS spectra after the Fourier transform. The main scattering signal of Pd─Cu DAC/Cr is situated at 1.50 Å, while the scattering signals of Pd SAC/Cr and Cu SAC/Cr samples are located at 1.43 Å. The coordination structures of Cr atoms are depicted in Figure S21 and Table S4 (Supporting Information). It reveals that the scattering distance of Cr─O path in Pd─Cu DAC/Cr (1.97 Å) is longer than that in Pd SAC/Cr (1.92 Å) and Cu SAC/Cr (1.93 Å), which implies that Cr─O bonds of H_2_CrO_4_ are stretched by Pd─Cu DAC. Combined with the above analysis, it is inferred that Pd─Cu diatomic pairs as dual‐active centers directly interact with two O atoms of H_2_CrO_4_, stretching Cr─O bonds, transferring electrons to activate H_2_CrO_4_, then accelerating reduction reactions of H_2_CrO_4_ to Cr(III).

### 2.5 Electronic Structure Changes of Pd─Cu DAC/Cr, Pd SAC/Cr, and Cu SAC/Cr Revealed by DFT Calculation

The differences in electronic structures and interaction relationships of three catalysts toward Cr(VI) were further studied by DFT calculations. The optimal interaction configurations of Pd─Cu DAC/Cr, Pd SAC/Cr, and Cu SAC/Cr are displayed in Figure S22 (Supporting Information), their corresponding adsorptive energies are calculated as −0.58, −0.10, and −0.30 eV, respectively. Pd and Cu atoms on Pd─Cu DAC interact and bond with two O atoms of H_2_CrO_4_ forming a stable interaction configuration of the bi‐centric bridge (Figure S22a, Supporting Information). In contrast, there is just a weak adsorption between Pd SAC (or Cu SAC) with H_2_CrO_4_ without forming chemical bonds (Figure S22b,c, Supporting Information). From the slice of the charge density difference plot in Figure 4f, it is clear that the electrons both of Pd and Cu atoms are transferred to their bonded O atoms of H_2_CrO_4_, respectively. Besides, the phenomena of obvious electron transfer are also observed between Pd and Cu atoms (Figure S23, Supporting Information), as well as between Pd─Cu atomic pairs and N─C substrate. But an extremely weak electron transfer is present between Pd single atom of Pd SAC and O atom of H_2_CrO_4_ (Figure S24a, Supporting Information), as well as between Cu single atom of Cu SAC and O atom of H_2_CrO_4_ (Figure S24b, Supporting Information), which further proves the weak chemical interaction of Pd SAC (or Cu SAC) with H_2_CrO_4_. These calculation results are consistent with the above XAFS and XPS results. Via analyzing PDOS diagrams of Pd, Cu, and O atoms in the bridging interaction configuration of Pd─Cu DAC/Cr (Figure S25, Supporting Information), it is found that the *d*‐orbital electrons of Pd atom and the *p*‐orbital of O atom overlap well in the range of −1.0–2.3 eV, and the *d*‐orbital electrons of Cu atom and the *p*‐orbital of O atom also coincided well in the range of −1.0–3.8 eV. These demonstrate the strong coupling behavior of Pd with O atoms and Cu with O atoms, transferring electrons to activate H_2_CrO_4_. The synergistic catalytic effect of Pd─Cu diatomic pairs jointly promotes the adsorption and reduction reactions of Cr(VI). Nevertheless, there is no apparent orbital electron overlap in the PDOS diagrams of Pd (or Cu), Cr, and O atoms on Pd SAC/Cr (Figure S26, Supporting Information) or Cu SAC/Cr (Figure S27, Supporting Information), reflecting a relatively weak coupling of Pd SAC or Cu SAC with Cr(VI).

Furthermore, Mulliken charge distribution of Cr, O, H, Pd, Cu, and N atoms in Pd─Cu DAC, Pd SAC, and Cu SAC before and after adsorbing H_2_CrO_4_ are compared in Tables S5 and S6 (Supporting Information). Mulliken charges of Pd and Cu atoms in Pd─Cu DAC/Cr are increased to 0.96 and 0.99 eV (from 0.92 and 0.95 eV) after H_2_CrO_4_ adsorption, respectively. But Mulliken charges of O_1_ and O_2_ that bonded with Pd or Cu atoms are decreased to −0.52 and −0.55 eV, respectively. The H_2_CrO_4_ adsorbed on Pd─Cu DAC obtains the most electron of 0.40*e* from the substrate material (Table S5, Supporting Information), compared to that on Pd SAC (−0.10 eV) and Cu SAC (−0.13 eV). These further certify that Pd─Cu DAC exhibits an outstanding catalysis ability to dedicate more electrons and accelerate the reduction reactions of Cr(VI). Therefore, the key role of the bridging coupling effect of heterogeneous Pd─Cu dual‐centers in catalyzing Cr(VI) reduction is proved from experimental and theoretical perspectives.

### 2.6 Structural Evolution of Pd─Cu DAC and Cu SAC During Electrochemical Reduction of Cr(VI) via In Situ XAFS Technique

**Figure 5:**
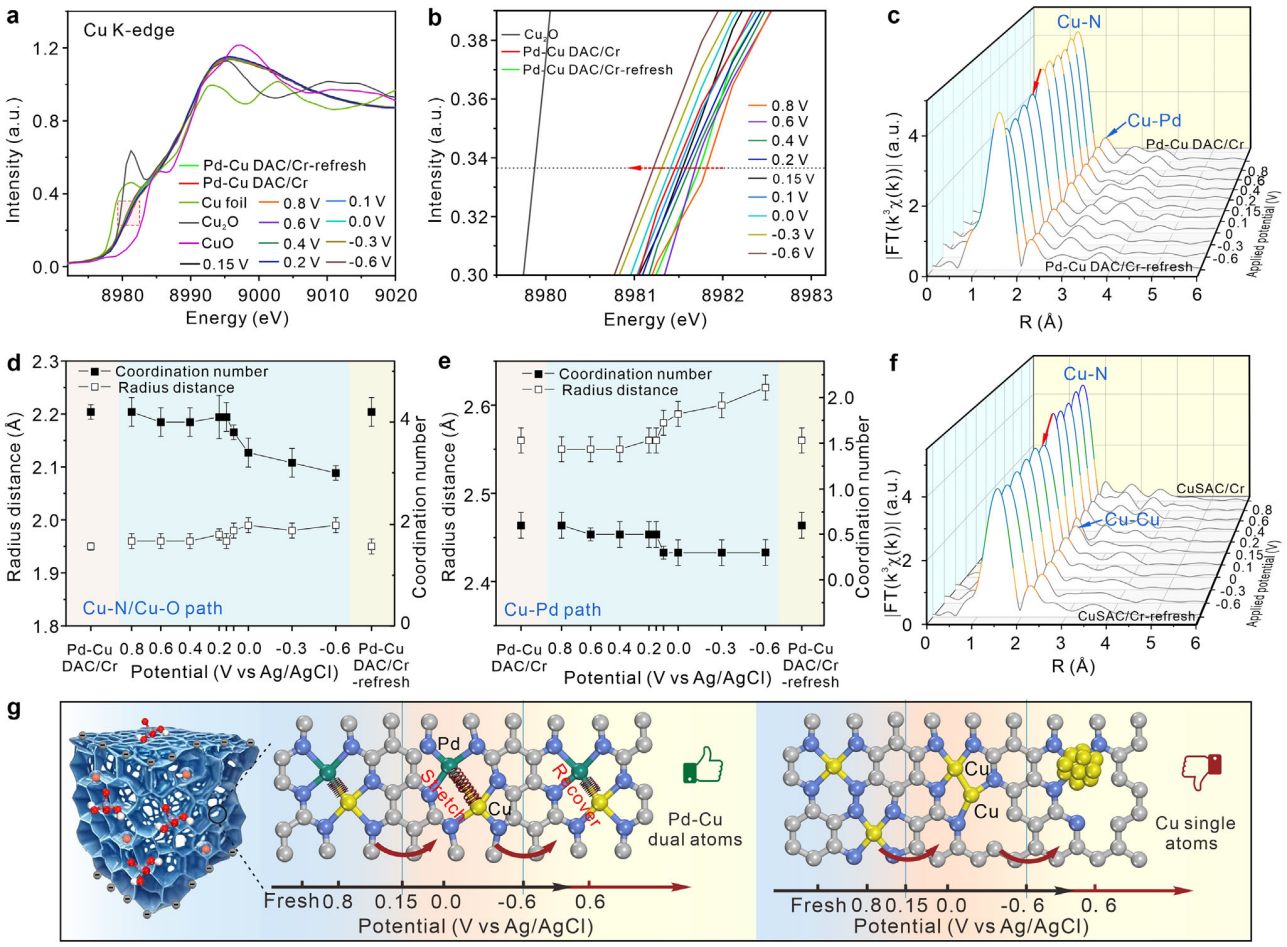
Study in structural evolution of catalysts by in situ XAFS technique. a) Normalized in situ XANES spectra of Cu K‐edge in Pd─Cu DAC/Cr under different applied potentials. b) Enlarged detail plot of the spectra in the red dotted box in Figure 5a. c) In situ EXAFS spectra of Cu K‐edge in Pd─Cu DAC/Cr under different potentials after Fourier transform without correcting for scattering phase shift. Variation on the radius distance and coordination number of d) Cu─N/Cu─O path and e) Cu─Pd path in Pd─Cu DAC/Cr under different potentials. f) In situ EXAFS spectra of Cu K‐edge in Cu SAC/Cr under different potentials after Fourier transform without correcting for scattering phase shift. g) Schematic diagrams of the structural evolution around Cu atoms in Pd─Cu DAC/Cr (left) and Cu SAC/Cr (right) driven by potential changes during Cr(VI) reduction reactions.

Additionally, the in situ XAFS technique was applied to research coordination structure evolutions and chemical environment variations of Cu atoms in Pd─Cu DAC and Cu SAC under different applied potentials during the electrochemical reduction processes of Cr(VI). The detailed experimental procedures are displayed in Supporting Information (Figure S28, Supporting Information). **Figure** **5**a shows XANES spectra of Cu K‐edge in Pd─Cu DAC/Cr collected at different potentials from 0.8 to −0.6 V. The partial spectra in the red dotted box of Figure 5a are amplified in Figure 5b. The red line denotes the Pd─Cu DAC/Cr without applied potential after immerging in the electrolyte containing 1000 ppb Cr(VI) for 180 s. It is observed that when the initial potential of 0.8 V was applied to the Pd─Cu DAC/Cr, the absorption edge of the Cu K‐edge obviously moved toward higher energy (orange line), indicating the increased oxide state of Cu atoms. Then, when the potential negatively changed from 0.8 to −0.6 V, the absorption edge of Cu K‐edge gradually shifted toward lower energy, demonstrating the successively decreased average valent state of Cu atoms driven by reduction potentials. Moreover, both the positive and negative shifts of Cu K‐edge absorption‐edge are between the Cu K‐edge spectra of Cu_2_O and CuO, reflecting that the average valent state of Cu atom just changes between +1 and +2 during reduction processes. In addition, after performing in situ XAFS and electrochemical experiments, we chose a potential of 0.6 V for reapplying to Pd─Cu DAC/Cr to excavate the chemical valence reversibility of Cu atom, which is marked as Pd─Cu DAC/Cr‐refresh. The pre‐edge of Cu K‐edge XANES spectra in Pd─Cu DAC/Cr‐refresh (green line) is very close to that in Pd─Cu DAC/Cr under 0.6 V (purple line), illustrating excellent reversibility and regeneration of Cu valent state in Pd─Cu DAC driven by the potential (Figure 5b).

Moreover, the coordination structure evolution of Cu atoms in Pd─Cu DAC under different potentials is also exploited. It is discovered from the EXAFS spectra (Figure 5c) that the intensity and position of the two main scattering signals (Cu─N/Cu─O path and Cu─Pd path) almost remain unchanged with potentials decreasing from 0.8 to 0.15 V in succession. Then, the intensity of the two scattering signals begins to decline apparently under 0.1 V and continuously decreases with potentials changing from 0.1 V to −0.6 V. However, their intensities in Pd─Cu DAC/Cr‐refresh obviously increase to the previous intensity in Pd─Cu DAC/Cr. Whereafter, the above EXAFS spectra are fitted and shown in Figure S29 and Table S7 (Supporting Information). The variation of the radius distance and coordination number of Cu─N/Cu─O and Cu─Pd paths with different applied potentials are depicted in Figure 5d,e. The radius distance of Cu─N/Cu─O scattering path almost has no obvious difference with potentials changing from 0.8 to 0.15 V (Figure 5d), then is slightly stretched to 1.99 Å (from 1.95 Å) as potentials continuously decrease to −0.6 V, which is attributed to the decreased oxide state of Cu atom affected by negative potentials. Meanwhile, its coordination number begins to fall at 0.1 V, then continuously decreases as the potential changes to −0.6 V in succession. The decreased coordination number of Cu─N/Cu─O path from 4.2 to 3.0 is ascribed to the release of the coordinated O atoms (in H_2_CrO_4_) from Cu atoms, unraveling the transformation of H_2_CrO_4_ to Cr(III) ions under reduction potentials. Similarly, the radius distance of Cu─Pd path remains unchanged during the potential range of 0.8–0.15 V (Figure 5e), then is stretched gradually to 2.62 from 2.56 Å as potentials decrease to −0.6 from 0.1 V. The stretched radius distance of Cu─Pd path is mostly caused by the decreased valent state of Cu atoms at −0.6 V. But the coordination number and radius distance of both Cu─N/Cu─O path and Cu─Pd path in Pd─Cu DAC/Cr‐refresh are basically the same as these in Pd─Cu DAC/Cr at 0.6 V, which proves that the evolution in chemical structures of Cu atoms exhibits satisfactory reversibility with potential changes. Notably, the changes in the chemical state and coordination structure of Cu atoms are reversibly driven by potentials. During Cr(VI) reduction reactions, the reversible stretching and recovering of Cu─Pd and Cu─N bonds driven by different potentials can be defined as the dynamic “spring‐like” effect. The corresponding schematic diagram of the “spring‐effect” on Pd─Cu DAC is displayed in the left part of Figure 5g. Therefore, combined with the electrochemical tests, in situ XAFS spectra certify the reversible valent state of Cu atoms and the dynamic “spring‐effect” of Cu─N and Cu─Pd bonds that can be reversibly stretched and recovered during reduction reactions of Cr(VI) to Cr(III). Such a reversible structure evolution of the dynamic “spring‐effect” guarantees the remarkable cyclic stability of Cu atoms and high catalytic activity of Pd─Cu DAC to form the stable bridging interaction configuration with H_2_CrO_4_ and transfer electrons efficiently accelerating the reduction reactions of Cr(VI).

In addition, the coordination structural evolution of Cu SAC/Cr was also explored via in situ EXAFS spectra (Figure 5f). The scattering signal of Cu─N path remains unchanged with potential altering from 0.8 to 0.2 V, but when the potential reaches 0.15 V, the signal intensity of Cu─N path significantly decreases with a new scattering signal emerging at 2–3 Å. The corresponding fitting results are displayed in Figure S30 and Table S8 (Supporting Information). The average coordination number of Cu─N bond is decreased to 2.92 at 0.15 V from 3.91, suggesting the breakage of Cu─N coordination bonds. Meanwhile, the new scattering signal is assigned to Cu─Cu metallic bond with an average coordination number of 0.47 and a bond length of 2.52 Å. The formation of Cu─Cu bonds and the decreased coordination number of Cu─N bonds illustrate the inferior stability of Cu SAC under applied potentials that tend to aggregate as dimer or small clusters. Then, with the potential changing to −0.6 V, the bond lengths of Cu─N and Cu─Cu bonds have almost no obvious variation. Thus, Cu elements exist as the form of Cu single‐atoms during the potential range of 0.8–0.2 V, and then part of them present as Cu─Cu dimers or small clusters under the potential range of 0.15–0.6 V. Finally, Cu SAC/Cr was regenerated at 0.6 V and remarked as Cu SAC/Cr‐refresh. The Cu─Cu bond still appears in Cu SAC/Cr‐refresh with an increased coordination number of 0.63. These indicate the irreversible atomic structure and poor stability of Cu SAC under applied potentials.

After stability tests of 10‐times cycle electrochemical experiments (Figure S14, Supporting Information), the chemical structure stability and atomic‐level dispersion state of dual‐atoms and single‐atoms were studied, and the relevant characterizations are depicted in Figures S31–S33 (Supporting Information). As displayed in SEM and HAADF‐STEM images (Figure S31, Supporting Information), the well‐maintained 3D porous cross‐linked structure and the clean surface of the used Pd─Cu DAC (Figure S31a, Supporting Information), as well as the pair‐dispersed bright spots of Pd and Cu sites (Figure S31b, Supporting Information), jointly prove the excellent structural stability and superior atomic‐level dispersibility of Pd─Cu dual atoms. The high‐resolution XPS (HR‐XPS) spectra of Pd 3d, and Cu 2p in the used Pd─Cu DAC after repeat electrochemical stability tests are shown in Figure S31c,d (Supporting Information). Figure S31c (Supporting Information) reflects that HR‐XPS peaks of Pd 3d_5/2_ and 3d_7/2_ in the used Pd─Cu DAC present at 337.4 and 342.6 eV, assigned to Pd^2+^, which are the same as those in fresh Pd─Cu DAC (Figure S5c, Supporting Information). Besides, as depicted in Figure S31d (Supporting Information), HR‐XPS peaks of Cu 2p_3/2_ and 2p_1/2_ in the used Pd─Cu DAC can be identified into four peaks, the characteristic peaks that appeared at 932.1 and 952.1 eV are assigned to Cu^+^, and the other two peaks at 934.4 and 954.4 eV are assigned to Cu^2+^. The binding energies of these four peaks are the same as those in fresh Pd─Cu DAC. Moreover, the relative content proportion of Cu^+^ in the used Pd─Cu DAC (71.0%) is very close to that in fresh Pd─Cu DAC (74.6%, Figure S5d, Supporting Information). These results confirm the chemical structural stability and robustness of the Pd─Cu DAC catalyst for catalyzing electrochemical detection of Cr(VI). Similarly, the isolated and well‐dispersed bright spots of Pd single‐atoms on the clean surface of the used Pd SAC demonstrate its outstanding atomic‐structural stability (Figure S32a,b, Supporting Information). The HR‐XPS spectra of Pd 3d_5/2_ and 3d_7/2_ in the used Pd SAC (Figure S32c, Supporting Information) indicate that the binding energies of two characteristic peaks assigned to Pd^2+^ (337.4 and 342.6 eV) are the same as those in fresh Pd SAC (Figure S5c, Supporting Information). No significant structural degradation was observed in the used Pd SAC. However, there are some small clusters/nanoparticles of Cu‐metal with distinct lattice fringes of (111) and (200) planes arising on the used Cu SAC surface (Figure S33a–c, Supporting Information), and the XRD pattern also shows obvious characteristic diffraction peaks of (111) and (200) planes of Cu metallic nanoparticles (Figure S33d, Supporting Information). Besides, the EXAFS spectra (Figure S33e,f, Supporting Information) of Cu SAC‐recycle after stability tests demonstrate that two main scattering signals of Cu─N and Cu─Cu paths appear with coordination numbers of 1.63 and 2.56, respectively. The decreased coordination number of Cu─N bonds and the increased coordination number of Cu─Cu bonds, compared to Cu SAC/Cr‐refresh, also prove the breakage of Cu─N bonds and the agglomeration of Cu single‐atoms forming Cu‐metallic clusters/nanoparticles. Combining the analysis results of in situ XAFS spectra in Figure 5f, we can suspect as follows, and the schematic diagram of the structural evolution of Cu SAC is displayed in the right part of Figure 5g. In the early stages of the potentials (0.8–0.2 V), Cu single‐atoms retained their original chemical structure and existed in isolation; then during Cr(VI) reduction reaction (0.2–0 V) and under the reduction potentials of 0–0.6 V, some Cu single‐atoms transformed into dimers or even nanoclusters; after several times of repeat cyclic‐stability tests, the agglomeration of Cu atoms occurred to form Cu‐metallic nanoparticles. It can be concluded that both Pd─Cu DAC and Pd SAC catalysts exhibit robust structural stability for sustained electrochemical detection of Cr(VI), but part of Cu single atoms in Cu SS2AC tend to agglomerate as Cu‐metallic nanoparticles during electrochemical reduction of Cr(VI). These results also certify the strong coupling of Pd atoms with Cu atoms in Pd─Cu DAC is greatly favorable for the superior instinct structural stability of Cu atoms under the electrochemical potentials.

### 2.7 Illustration on Reaction Energy Barriers via Theoretical Calculation

**Figure 6:**
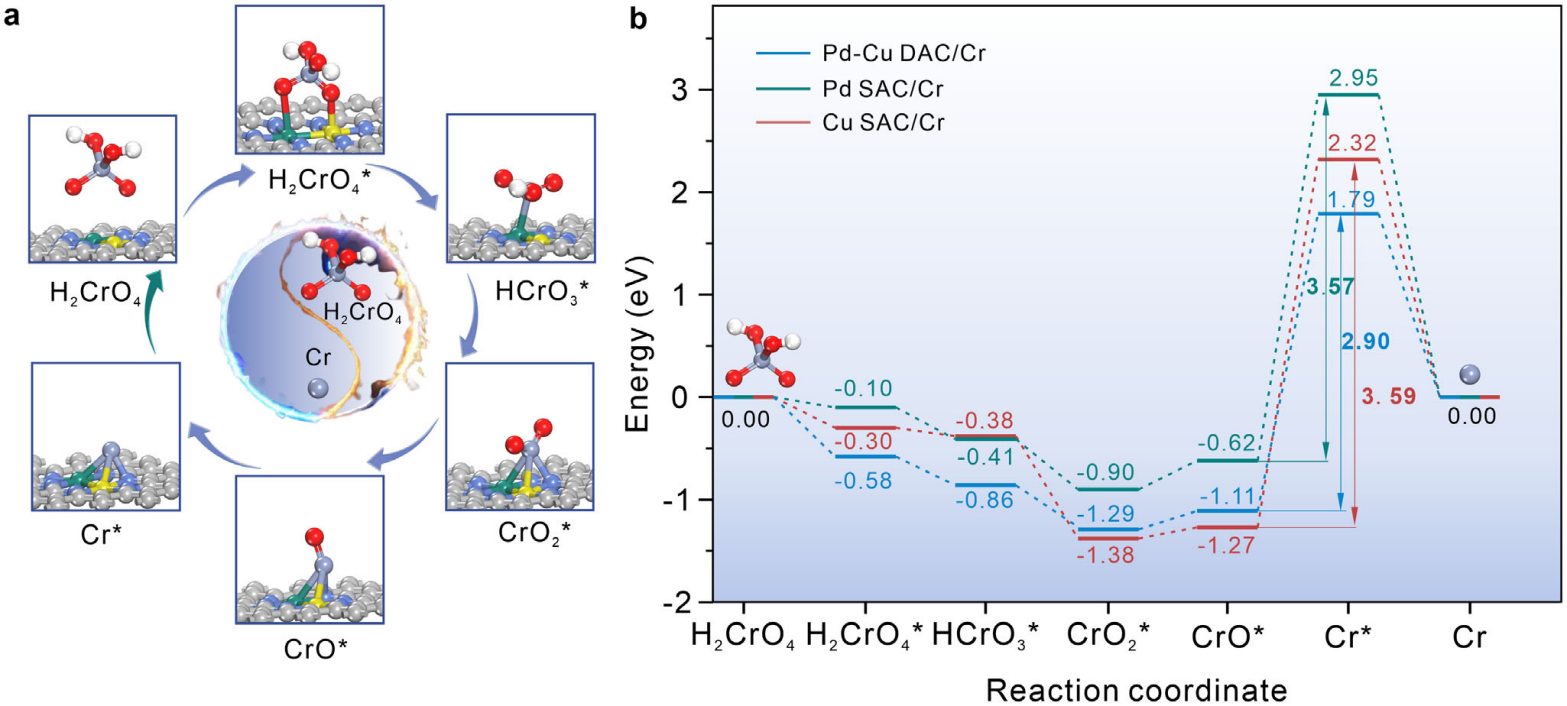
Theoretical calculations for reaction energy barriers. a) The interaction configurations of Cr(VI)‐transition states with Pd─Cu DAC during stepwise reduction processes. b) Comparison in the relative free energies of H_2_CrO_4_ stepwise reduction on Pd─Cu DAC, Pd SAC, and Cu SAC. The symbol (*) indicates the surface adsorbed states of Cr(VI) intermediates.

In addition, the corresponding reaction energy barriers are illustrated via theoretical calculations. The optimal interaction configurations of Pd─Cu DAC, Pd SAC, and Cu SAC adsorbing Cr(VI) intermediates during reduction processes were optimized via DFT calculation and displayed in **Figure** **6**a and Figures S34–S36 (Supporting Information), respectively. Besides, the relative free energy of H_2_CrO_4_ stepwise reduction on Pd─Cu DAC, Pd SAC, and Cu SAC catalysts is compared in Figure 6b, and their energy step diagrams display a similar tendency that shows a continuous downward trend during the process of H_2_CrO_4_ reducing into CrO_2_*, indicating that these are spontaneous processes without other external energy demanded. Afterward, the relative free energies are elevated during CrO_2_* stepwise reducing to CrO* and Cr*, and the required energy is provided by applied potentials. The transformation process of CrO_2_* into Cr* is the rate‐determining step of the entire electrochemical reduction reaction. The energy required in the rate‐determining step of Cr(VI) reduction reactions is the least on Pd─Cu DAC (2.90 eV), in comparison with that on Pd SAC (3.57 eV) and Cu SAC (3.59 eV), which proves the lowest reaction barriers and the fastest reduction rate on Pd─Cu DAC, thereby accelerating the dissociation of Cr‐O bonds and achieving high reaction efficiency.

Therefore, the dynamic “spring‐effect” of Cu─Pd and Cu─N bonds greatly contributes to the remarkable structure stability under applied potentials and their high reactivity for electrochemical reduction of Cr(VI) in a strong acid electrolyte. Besides, the mutually modulated electron‐orbit coupling effect between Pd and Cu atoms effectively prevents the isolated Cu single‐atoms from aggregating as dimers or even metallic nanoparticles, which is also favorable for advancing the catalytic activity of Pd single‐atoms. Pd─Cu dual‐atoms as two active centers interact with two O atoms of H_2_CrO_4_, forming the bridging interaction configuration and transferring electrons to activate H_2_CrO_4_, then accelerating their reduction rates efficiently.

## 3 Conclusion

In summary, a superior Pd─Cu dual‐atomic catalyst with the modulated inter‐metal coupling of PdN_3_─CuN_3_ coordination is developed to effectively surmount the common cyclic instability and deactivation problem of active Cu single‐atoms during reaction processes, meanwhile remarkably improving the catalytic activity of Pd single‐atoms for the electrochemical reduction of Cr(VI). In situ XAFS technology unravels that with the potential swept from 0.8 to −0.6 V, the reversible valent state change of Cu atoms is decreased between +1 and +2, meanwhile, the dynamic “spring‐like” behavior of Cu─Pd and Cu─N bonds reversibly stretching and recovering can be driven by different applied potentials during Cr(VI) reduction reactions, and mutually modulated electron‐orbit coupling effect between Pd and Cu atoms ensures the glorious cycle stability and stimulates catalytic reactivity of Pd─Cu DAC. But once there is no coupling and restriction of Pd atoms with Cu atoms, isolated Cu single atoms on Cu SAC irreversibly form into dimers or even small metallic nanoparticles with the change of potential, impeding the catalytic performance. Furthermore, the Pd─Cu DAC catalyst achieves ultra‐high turnover frequency in the catalysis reduction of Cr(VI) to Cr(III) and the lowest overpotential with a high response sensitivity of 0.51 µA ppb^−1^. It is discovered that the Pd─Cu diatomic centers as double active sites couple with two O atoms of H_2_CrO_4_ and transfer electrons to H_2_CrO_4_, which markedly expedites their reduction reaction processes and decreases catalytic reaction energy barriers. This work opens up a feasible route to engineer potential‐stable atomic‐level catalysts for wider catalytic applications and also provides constructive insights into exploring the structural evolution of catalysts during reaction processes from the in situ perspective.

## 4 Experimental Section

The detailed experimental processes were available in the Supporting Information.

## Conflict of Interest

The authors declare no conflict of interest.

## Supporting information



Supporting Information

## Data Availability

The data that support the findings of this study are available from the corresponding author upon reasonable request.
