# Supplementary material for: Potential‐Driven Dynamic Spring‐Effect of Pd─Cu Dual‐Atoms Empowered Stability and Activity for Electrocatalytic Reduction
Source: Adv Sci (Weinh). 2025 Apr 26;12(28):2501393. doi: 10.1002/advs.202501393 (PMC12302595; doi:10.1002/advs.202501393)
Supplement: Supplementary file 1 — Supporting Information [file ADVS-12-2501393-s001.docx]

Supporting Information

Potential-Driven Dynamic Spring-Effect of Pd-Cu Dual-Atoms Empowered Stability and Activity for Electrocatalytic Reduction

Pei-Hua Li, Yuan-Fan Yang, Zong-Yin Song, Bo Liang, Yong-Huan Zhao, Xin Cai, Zi-Hao Liu, Jing-Yi Lin, Meng Yang, Xiangyu Xiao,* Jing Zhang,* Wen-Qing Liu,* Xing-Jiu Huang*

**Contents**

**1.** **Experimental Section**

1.1. Chemical reagents

1.2. Instruments

1.3. Synthesis of Pd-Cu DAC, Pd SAC, and Cu SAC catalysts

1.4. Preparation of catalyst ink and modified electrodes

1.5. Electrochemical tests

1.6. Determination of turnover frequency

1.7. Adsorption experiments.

1.8. XAFS data analysis

1.9. In-situ XAFS experiments

1.10. DFT theoretical calculations

**2. Figures**

**Figure S1.** SEM, TEM, HAADF-STEM, and mapping images of Pd SAC.

**Figure S2.** SEM, TEM, HAADF-STEM, and mapping images of Cu SAC.

**Figure S3.** Comparison in XRD patterns, Raman spectra, XPS spectra, and EDS of Pd-Cu DAC, Pd SAC, and Cu SAC catalysts.

**Figure S4.** N_2_ adsorption-desorption isotherms and aperture distribution of Pd-Cu DAC, Pd SAC, Cu SAC, and N-C.

**Figure S5.** High-resolution XPS spectra of C 1s, N 1s, Pd 3d, and Cu 2p in Pd-Cu DAC, Pd SAC, and Cu SAC catalysts.

**Figure S6.** Wavelet transformed EXAFS spectra of Pd K-edge and Cu K-edge.

**Figure S7.** k^3^χ(k) oscillation curves corresponding to Figure 2a-d.

**Figure S8.** The atomic structure models of Pd-Cu DAC, Pd SAC, and Cu SAC catalysts optimized by DFT calculation.

**Figure S9.** Mulliken charge distribution of Pd SAC and Cu SAC catalysts.

**Figure S10.** Charge density difference plots of the horizontal slices through the Pd (or Cu), N, and C atomic layers on Pd SAC and Cu SAC catalysts.

**Figure S11.** PDOS diagrams of Pd and Cu single atoms in Pd-Cu DAC, Pd SAC, and Cu SAC.

**Figure S12.** EIS diagrams and CV curves of Pd-Cu DAC, Pd SAC, and Cu SAC.

**Figure S13.** LSV current signals of 200 and 400 ppb Cr(VI) measured by Pd-Cu DAC, Pd SAC, and Cu SAC.

**Figure S14.** The stability experiments of Pd-Cu DAC, Pd SAC, and Cu SAC electrodes in the repeated determination of Cr(VI).

**Figure S15.** HR-XPS spectra of Cr 2p, Pd 3d, and Cu 2p in Cu SAC/Cr samples.

**Figure S16.** HR-XPS spectra of Pd 3d and Cu 2p in Pd SAC/Cr and Cu SAC/Cr.

**Figure S17.** Fourier transformed Pd K-edge EXAFS spectra of Pd SAC/Cr and Cu K-edge EXAFS spectra of Cu SAC/Cr and their fitting curve.

**Figure S18.** The corresponding k^3^χ(k) oscillation curves of Figure 4c and 4d.

**Figure S19.** The corresponding k^3^χ(k) oscillation curves of Figure S17.

**Figure S20.** Normalized Cr K-edge XANES spectra in Pd-Cu DAC/Cr, Pd SAC/Cr, and Cu SAC/Cr samples.

**Figure S21.** Fourier transformed Cr K-edge EXAFS spectra and their fitting curves in Pd-Cu DAC, Pd SAC/Cr, and Cu SAC/Cr samples.

**Figure S22.** Optimal adsorption configurations of Pd-Cu DAC/Cr, Pd SAC/Cr, and (c) Cu SAC/Cr.

**Figure S23.** Charge density difference plot of the horizontal slices through the Pd, Cu, and N atomic layers on Pd-Cu DAC/Cr.

**Figure S24.** Charge density difference plots of the vertical slices through the Pd (or Cu), Cr, and O atomic layers on Pd SAC/Cr and Cu SAC/Cr.

**Figure S25.** PDOS diagrams of Pd, Cu, and O atoms in Pd-Cu DAC/Cr.

**Figure S26.** PDOS diagrams of Pd, N, Cr, and O atoms in Pd SAC/Cr.

**Figure S27.** PDOS diagrams of Cu, N, Cr, and O atoms in Cu SAC/Cr

**Figure S28.** Operation procedure of in-situ XAFS technique combined with electrochemical tests.

**Figure S29.** In-situ EXAFS spectra of Cu K-edge in Pd-Cu DAC/Cr with different applied potentials and the corresponding fitting curves.

**Figure S30.** In-situ EXAFS spectra of Cu K-edge in Cu SAC/Cr with different applied potentials and the corresponding fitting curves.

**Figure S31.** SEM image, HAADF-STEM image, and HR-XPS spectra of Pd 3d and Cu 2p in the used Pd-Cu DAC after electrochemical stability tests.

**Figure S32.** SEM image, HAADF-STEM images, and Pd 3d HR-XPS spectra of the used Pd SAC after electrochemical stability tests.

**Figure S33.** SEM image, TEM images, XRD pattern, and Cu K-edge EXAFS spectra of the used Cu SAC after electrochemical stability tests.

**Figure S34.** Side view and top view of the optimal interaction configuration of Pd-Cu DAC with Cr(VI)-transition states.

**Figure S35.** Side view and top view of the optimal interaction configuration of Pd SAC with Cr(VI)-transition states.

**Figure S36.** Side view and top view of the optimal interaction configuration of Pd SAC with Cr(VI)-transition states.

**3. Tables**

**Table S1.** The fitting results and parameters of Pd K-edge EXAFS spectra in Pd SAC and Pd-Cu DAC before and after interacting with Cr(VI).

**Table S2.** The fitting results and parameters of Cu K-edge EXAFS spectra in Cu SAC and Pd-Cu DAC before and after interacting with Cr(VI).

**Table S3.** Comparison in Cr(VI) reduction results achieved by the noble metal materials modified electrodes that previously reported.

**Table S4.** Fitting results of Cr K-edge EXAFS spectra in Pd-Cu DAC/Cr, Pd SAC/Cr, and Cu SAC/Cr.

**Table S5.** Mulliken charges of Cr, O, and H atoms in H_2_CrO_4_ before and after adsorbing with Pd-Cu DAC, Pd SAC, and Cu SAC.

**Table S6.** Mulliken charges of Pd, Cu, N atoms in Pd-Cu DAC, Pd SAC, and Cu SAC before and after adsorbing H_2_CrO_4_.

**Table S7.** Fitting results of Cu K-edge EXAFS spectra in Pd-Cu DAC collected during the in-situ electrochemical reduction of Cr(VI) under different applied potentials.

**Table S8.** Fitting results of Cu K-edge EXAFS spectra in Cu SAC/Cr collected during the in-situ electrochemical reduction of Cr(VI) under different applied potentials.

**4. References**

**1. Experimental Section**

**1.1. Chemical reagents**

All reagents were analytically pure and used without any other purification. The standard solution of 1000 ppm Cr(VI) was supplied by Guobiao (Beijing) Testing & Certification Co., Ltd. (China). Other chemical reagents were purchased from Shanghai Macklin Biochemcial Co., Ltd. The aqueous solutions were prepared by deionized water with a resistivity of 18.2 MΩ cm^-1^.

**1.2. Instruments**

XRD patterns were recorded via a Philips X’Pert Pro Super diffractometer with Cu *K*α radiation (λ=1.54 Å). SEM images were performed on a Quanta 200 FEG field-emission scanning electron microscopy. TEM and HAADF-STEM images were obtained from a JEOL ARM-200F field-emission transmission electron microscope. XPS spectra were collected from a VG ESCALAB MKII spectrometer with a Mg Kα X-ray source (1253.6 eV, 120 W). The contents of palladium and copper elements were analyzed by ICP-MS with a wavelength of 1890 nm (Agilent 7800). Raman spectra were operated on a Lab RAM HR800 confocal microscope Raman system (Horiba Jobin Yvon, Inc., USA). The whole electrochemical tests were executed on a CHI760E computer-controlled potentiostat (ChenHua Instruments Co., Shanghai, China).

**1.3. Synthesis of Pd-Cu DAC, Pd SAC, and Cu SAC catalysts**

For the Pd-Cu DAC sample, 4.64 mg of PdN_4_H_12_Cl_2_·H_2_O, 3.54 mg of CuCl_2_ (98%), and 1380 mg of H_3_NO·HCl (98.5%) were dissolved in 80 mL of deionized water, then stirred well to form a uniform mixed solution for 20 min. Subsequently, 288 mg of C_6_H_12_O_6_ (≥99.5%) was evenly dispersed in an 80 mL ethanol solution. [Afterward](javascript:;), the above-uniform solution was poured into the ethanol solution slowly and then stirred rapidly for 1 h. The fully mixed solution was dried at 70 ^o^C in an oven. After that, the dried solid powder was ground and heated at 600 ^o^C for 4 h in a tube furnace under the N_2_ gas atmosphere (heating rate 5 ^o^C min^-1^). After cooling down, the solid powder was evenly ground, then immersed in 1 M H_2_SO_4_ for 8 h to activate metallic atoms and remove by-products. Finally, the sample was collected via centrifugal separation and washed with deionized water several times. The Pd-Cu DAC catalyst was achieved after drying at 60 °C. The corresponding synthesis principle is based on the strong chelation effect between Cu^2+^ ions from CuCl_2_ and the lone pair electron of NH_3_ molecule from PdN_4_H_12_Cl_2_·H_2_O to construct Pd/Cu complex containing fixed-adjacent Pd-NH_3_-Cu atomic structures. Then, these Pd/Cu complexes were spatially isolated by glucose-derived polymer chains to guarantee the random and uniform distribution of the dual-metal precursors. This spatial confinement effectively prevents metal aggregation during the sintering process. During the sintering process, glucose chains were carbonized to form the carbon substrate, H_3_NO·HCl was heated thermally decomposed to produce NH_3_ molecules, etching the carbon substrate into a porous structure. Meanwhile, NH_3_ in Pd-NH_3_-Cu structures escaped from the carbon substrate, forming the adjacent Pd-Cu dual atom structures.

In addition, the feed ratio of the reactants is very important to ensure the uniformity of diatomic synthesis, which should guarantee the sufficient reaction between Cu^2+^ and PdN_4_H_12_Cl_2_·H_2_O and also avoid surplus reactants causing the migration and agglomeration of metal atoms. Besides, the calcination temperature, heating rate, calcination time, and atmosphere are vital to the uniformity of diatomic synthesis. When calcination temperature is higher than 600°C may result in Cu atom overflow or Pd atom migration. Relatively low calcination temperature (<600°C) and inadequate calcination time (<4 h) will hinder the complete elimination of NH_3_ ligands or nitrogen-containing intermediates from the Pd-NH_3_-Cu dual-atom structures. Residual N species could alter the local coordination environment of dual atoms or induce partial structural relaxation, thereby influencing the final atomic configuration and distance consistency. A slow heating rate of 5 ^o^C min^-1^ is beneficial to the metallic atoms gradually adapting to the thermal environment, preventing sudden migration or agglomeration. Calcination atmospheres affect the oxidation state and migration behavior of Pd and Cu metal atoms. Reducing atmospheres (such as H₂/Ar) may reduce the overflow temperature of metal atoms and increase the probability of metallic atoms overflowing, while the inert atmosphere of N₂ is favorable for maintaining the thermostability of metallic atoms. Therefore, the calcination temperature of 600 ^o^C, calcination time of 4 h, heating rate of 5 ^o^C min^-1^, and the calcination atmosphere of N_2_ were determined. This strategy guarantees the complete removal of NH_3_ molecules while suppressing the migration of Pd or Cu atoms, thereby locking the Pd-Cu dual-atom sites in adjacent configurations. The optimized thermal conditions minimize kinetic variations and stabilize the consistent Pd-Cu dual-atom architecture on the carbon substrate.

For the Pd SAC sample, 11.56 mg of PdN_4_H_12_Cl_2_·H_2_O and 1380 mg of H_3_NO·HCl were uniformly dissolved in 80 mL of deionized water, which then was poured into 80 mL ethanol solution containing 288 mg of C_6_H_12_O_6_. The other synthesis steps were the same as those of Pd-Cu DAC.

For the Cu SAC sample, 5.90 mg of CuCl_2_ and 1380 mg of H_3_NO·HCl were uniformly dissolved in 80 mL deionized water, which then was poured into 80 mL of ethanol solution containing 288 mg of C_6_H_12_O_6_. The other synthesis steps were the same as those of Pd-Cu DAC.

**1.4. Preparation of** **catalyst ink and modified electrodes**

A homogeneous catalyst ink was prepared by dispersing 5.0 mg of catalyst powder (Pd-Cu DAC, Pd SAC, or Cu SAC) in a mixture of 4900 μL deionized water and 100 μL Nafion solution (5 wt%). Then, the mixture was sonicated with the ice water in an ultrasonicator for 30 min to ensure complete dispersion and avert the thermal degradation of the catalyst. Next, 10 μL of this suspension solution (1 mg mL^-1^) was dropwise coated on the working carbon electrode surface of the screen-printed carbon electrode (SPCE, Metrohm China Ltd., 3 mm diameter) by a micropipette, followed by drying naturally to form a uniform thin film on the electrode surface. The modified electrode was finished and can be used for electrochemical experiments.

**1.5. Electrochemical tests**

Before electrochemical tests, N_2_ gas passed through 10 mL of 0.5 M H_2_SO_4_ solution for 20 min to expel dissolved oxygen. Subsequently, the electrochemical reduction of Cr(VI) was executed via linear sweep voltammetry (LSV). Firstly, the pre-enrichment time was set as 200 s to accumulate Cr(VI), which just relied on the adsorption capacity of catalysts at the electrode surface without any applied potential. Immediately, the potential that was applied to the working electrode consecutively varied from 0.8 V to -0.6 V (vs Ag/AgCl) with a scanning rate of 0.1 V s^-1^. Meanwhile, the curves of the current(I) as a function of the potential (E) were recorded with the different concertation of Cr(VI). The integral area of the current represents the amount of Cr(VI) involved in the reduction reaction to Cr(III). Later, the working electrode was applied to a potential of 0.6 V for 100 s to regenerate the electrode surface.

**1.6. Determination of** **turnover frequency**

Turnover frequency (TOF) is a vital indicator to reflect catalytic reaction rates and activity of catalysts, which refers to the product amount generated by the unit metallic site during a unit of time. The TOF value is calculated according to the Equation S1 below:^[1]^

$TOF=\frac{n}{N\times t}$ ( S1)

*n* is the number of Cr(VI) transforming into Cr(III). *N* is the number of metallic sites modified on the electrodes, and it represents the number of Pd or Cu single atoms, and Pd-Cu dual-atoms that participate in the reduction reaction of Cr(VI) in this work. *t* denotes the actual reaction time (unit: s).

Then, the value of *n* was obtained from the following Equation S2, S3, S4:

$n=\frac{Q}{3\times e}$ (S2)

$t=\frac{\triangle E}{v}$ (S3)

$Q=It=I(E)\frac{\triangle E}{v}=\frac{I(E)\times\triangle E}{\nu}=\frac{\int_{E1}^{E2} I\left( E \right)dE}{\nu}$ (S4)

where *Q* is the total coulomb of transferred electrons in the reduction process (unit: C). *e* is the electron charge and its value is 1.6x10^-19^ C. $\Delta E$ is the potential range occurring catalytic reduction reaction of Cr(VI) into Cr(III) (unit: V). ν is the potential scan rate and it was set to be 0.1 V in the LSV detection process in this work. Thus, the value of *n* can be confirmed as the Equation S5 below:

$n=\frac{Q}{3\times e}=\frac{\int_{E1}^{E2} I\left( E \right)dE}{3\times e\times\nu}$ (S5)

$\int_{E1}^{E2} I\left( E \right)dE$ can be gained via calculating the integrated area of the peak current in LSV curves.

The number of active sites (*N*) that participated in the catalytic reaction on the surface of Pd-Cu DAC, Pd SAC, and Cu SAC electrodes, can be calculated *via* the Equation S6：

$N=\frac{m}{M}\times N_{A}$ (S6)

where *m* indicates the mass of active atoms on the electrode surface (unit：g). *M* represents the molar mass of a metallic atom (Pd, 106.4 g mol^-1^; Cu, 63.5 g mol^-1^;). *N_A_* denotes the Avogadro constant of 6.02×10^23^.

**1.7. Adsorption experiments**.

10 mg of solid samples of Pd-Cu DAC, Pd SAC, or Cu SAC were evenly dispersed in 10 mL of 0.5 M H_2_SO_4_ solution, which contains 1000 ppb Cr(VI). Afterwards, the uniform suspension was oscillated in a shaker for 12 h at 25 ^o^C. Subsequently, the solid material was collected by centrifugation and washed once using 0.5 M H_2_SO_4_ solution to remove superfluous Cr(VI). Finally, the sample was freeze-dried for 24 h and put into further measurements.

**1.8. XAFS data analysis**

The XAFS spectra of Pd K-edge (*E*_0_ =24350 eV) and Cr K-edge (*E*_0_ = 5989 eV) were collected in Lytle-fluorescence mode at the BL14W1 beamline of Shanghai Synchrotron Radiation Facility (SSRF). The XAFS spectra of Cu K-edge (*E*_0_ = 8979 eV) were acquired in Lytle-fluorescence mode at the BL11B beamline of SSRF. The background signals of raw data were deducted by Athena software, then the signal intensity was normalized. Then, the treated data were applied to the Fourier transform and fitted their profiles via Artemis software

**1.9. In-situ XAFS experiments**

The in-situ XAFS measurements were operated at beamline 1W1B of the Beijing Synchrotron Radiation Facility (BSRF). A double-crystal Si(111) monochromator was employed to achieve an energy resolution of ΔE/E ≈ 2×10⁻⁴, with a Rh-coated harmonic rejection mirror to suppress higher-order harmonics. The Cu K-edge spectra (E₀ = 8979 eV) were collected in Lytle-fluorescence mode. Soller slits (divergence angle <5 mrad) were integrated to minimize scattering noise and enhance signal-to-noise ratio. For the design of electrochemical in-situ cell, a custom-designed polytetrafluoroethylene (PTFE) electrochemical cell was equipped with a Kapton film X-ray window (effective detection area: 0.5 cm diameter). The 30 mL electrolyte of 0.5 M H_2_SO_4_ was pre-saturated with high-purity N_2_ for 20 min to expel dissolved oxygen. For the electrochemical three-electrode system, the carbon paper (1.5*1.5 cm) was used as the working electrode, the Ag/AgCl electrode (3 M KCl) as the reference electrode, and a Pt wire electrode served as the counter electrode. In order to obtain a high signal-to-noise ratio spectra signal of Cu K-edge, we expanded the amount of catalyst by ten times. 320 μL prepared suspension solution of Pd-Cu DAC or Cu SAC catalyst ink (10 mg mL^-1^) was evenly dropwise coated in batches (4 times, 80 μL per time) on the carbon paper electrode, ensuring the uniformity and compactness of the catalyst on the electrode surface. Finally, a modified carbon paper electrode was yielded. The carbon cloth was adopted as the wire to connect the carbon paper electrode and the potentiostat, transmitting the electric current. For the in-situ XAFS-electrochemical tests, a CHI 760E potentiostat was used to apply stepwise potentials after stabilizing the open-circuit potential. In addition, each potential was applied for 10 min to the modified carbon-paper electrodes to reach a steady state, with XAFS data collected during the 8–10 min window to avoid transient effects. XANES and EXAFS spectra were acquired in quick-scan mode (energy step: 0.3 eV, dwell time: 1 s) and averaged three scans to improve data quality. Other in-situ experimental conditions were the same as that in electrochemical tests in the laboratory. For the XAFS data validation, the energy calibration was performed using a Cu foil standard. Background subtraction and normalization were executed using the IFEFFIT software package.

**1.10. DFT theoretical calculations**

The theoretical calculations were performed utilizing Generalized Gradient Approximation (GGA), and the Perdew-Burke-Ernzerhof (PBE) exchange-correlation parameterization of the first principle density functional theory (DFT) was carried out with CASTEP code. For self-consistent calculations, 10^-6^ eV was used as the convergence energy threshold. The geometry optimization parameters and convergence criteria of total energy, maximum atom force, maximum atom stress, and maximum displacement, were 1×10^-5^ eV/atom, 0.03 eV/Å, 0.05 Gpa, 0.001 Å, respectively. The geometric optimization algorithm adopted BFGS algorithm. Then, the pseudopotential Schrödinger equation was self-consistently solved. The kinetic energy cutoff for the plane wave basis expansion was set as 400 eV in this system, and the calculation accuracy was fixed as fine. Besides, a graphene supercell of 6×6 was employed to simulate the substrate of N-doped C materials with ~15 Å vacuum space in z-direction. The Brillouin zone integration was conducted using 2×2×1 gamma-centered k-point sampling in the entire calculation process. The Mulliken charge equation was adopted to determine the electronegativity of target atoms.

The adsorption energy (E_ads_) of an H_2_CrO_4_ on the substrate was defined as below the Equation S7:

E_ads_ = E_Cr/sub_ — E_sub_ — E_Cr_  (S7)

where E_Cr/sub_, E_sub_, and E_Cr_ signify the energy of H_2_CrO_4_ adsorbed on the catalyst surface, the energy of the clean surface, and the energy of H_2_CrO_4_ in the 15x15x15 box, respectively.

Herein, H_2_CrO_4_ reductions were considered as follows Equation S8-S13):

1. * + H_2_CrO_4_ → H_2_CrO_4_* (S8)
2. H_2_CrO_4_* + H^+^ + e^−^→ HCrO_3_* + H_2_O (S9)
3. HCrO_3_* + H^+^ + e^−^→ CrO_2_* + H_2_O (S10)
4. CrO_2_* + H_2_O + 2e^−^→ CrO* + 2OH^−^ (S11)
5. CrO* + H_2_O+ 2e^−^→ Cr* + 2OH^−^ (S12)
6. Cr* → * + Cr (S13)

The asterisk (*) denotes the active site of catalysts. The free energies of the H_2_CrO_4_ reduction steps were calculated via this equation: ΔG = ΔE_DFT_ + ΔE_ZPE_ − TΔS, here ΔE_DFT_ was the DFT electronic energy difference between each step, ΔE_ZPE_ and ΔS were the correction of zero-point energy and the variation of entropy, respectively, which were obtained by vibration analysis, T was the temperature (T = 300 K).

**2. Figures**


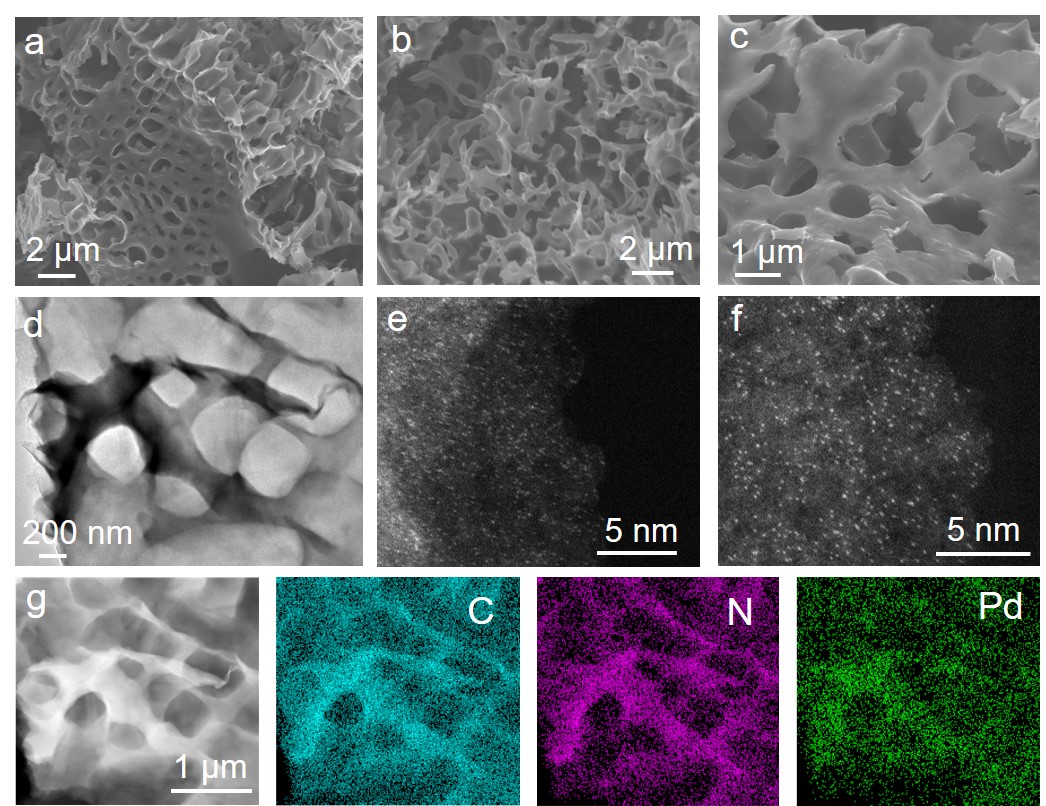


**Figure S1.** a-c) SEM and d) TEM images of Pd SAC catalyst. e, f) HAADF-STEM images of Pd SAC with aberration correction. The white bright spots denote metallic Pd single atoms. g) Elemental mapping images of C, N, and Pd elements in Pd SAC catalyst.

As observed in SEM and TEM images (Figure S1a-d), the Pd SAC catalyst exhibited a porous frame structure composed of thin sheets. Aberration-corrected HAADF-STEM images in Figures S1e and f show that a lot of isolated bright spots, on behalf of metal Pd atoms, were randomly dispersed on the base material, and no converged bright spots can be observed. These prove that all of the Pd atoms appeared in the form of single atoms and were stably anchored on the N-C substrate. Elemental mapping images (Figure S1g) reflect that C, N, and Pd atoms were uniformly distributed over the material structure.


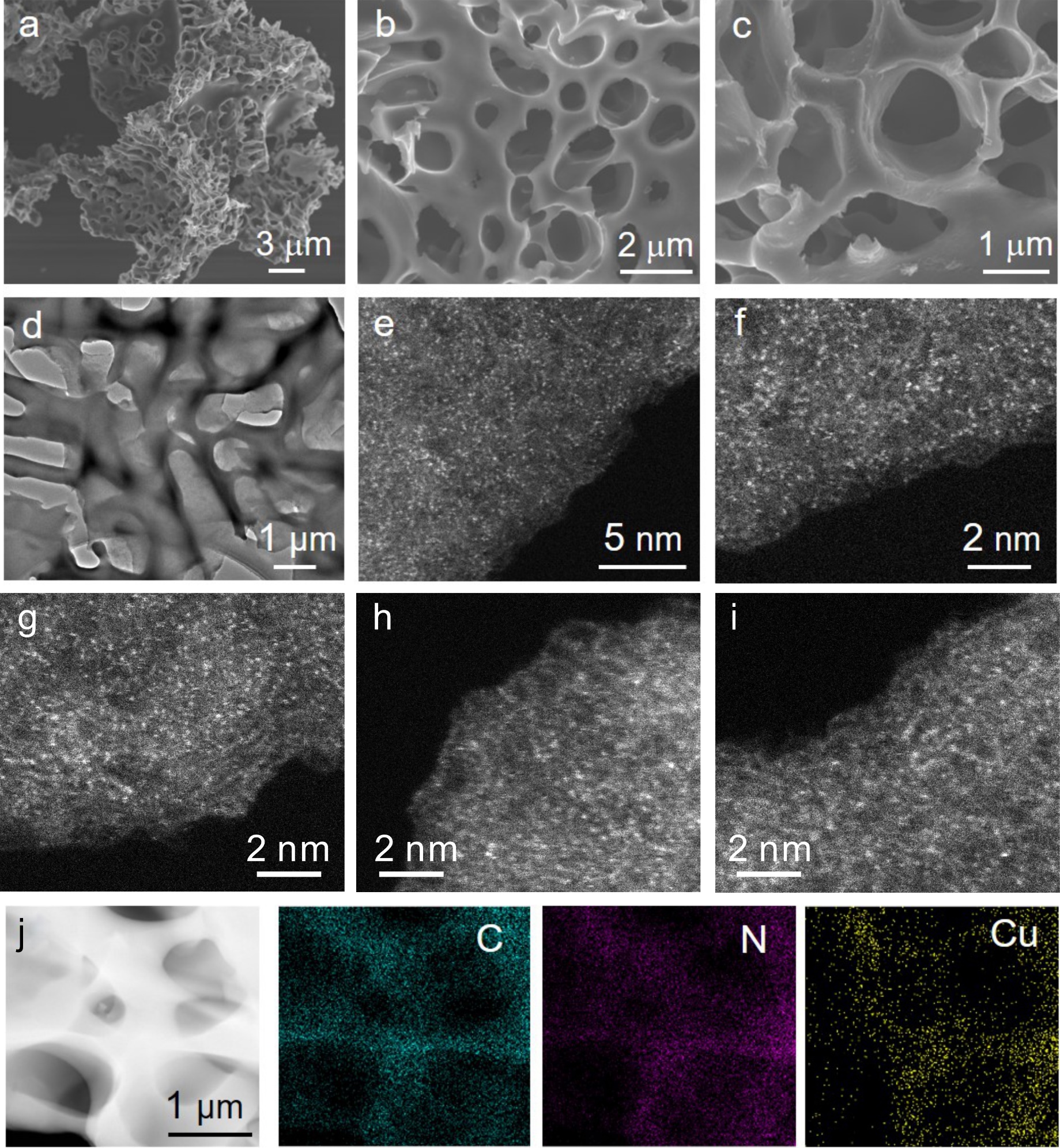


**Figure S2.** a-c) SEM and d) TEM images of Cu SAC catalyst. e-i) HAADF-STEM images of Cu SAC with aberration correction. The white bright spots denote metal Cu single atoms. j) Elemental mapping images of C, N, and Cu elements in Cu SAC catalyst.

As observed in SEM and TEM images (Figure S2a-d), the Cu SAC catalyst also possessed a porous frame structure composed of thin sheets. Aberration-corrected HAADF-STEM images in Figure S2e-i display that a large number of bright spots representing metal Cu atoms were randomly scattered on the base material, and there were no clustered dots of Cu atoms arose, which demonstrates the atomically dispersed form of Cu atoms over the N-C substrate. Elemental mapping images (Figure S2g) prove that the main elements of C, N, and Cu were homogeneously dispersed over the material structure. Notably, a few diatomic sites of Cu localized on the N-C support material are observed in Figure S2e-i, and a rational reason is that surface defects and edge dangling bonds of N-C materials possess high surface energy and can serve as "trap sites" for Cu atom migration, driving the dynamic aggregation of adjacent Cu single atoms to form diatomic structures during the high-temperature sintering process. Other possible reasons are the projective superposition effect and noise/resolution limitations of the field-emission transmission electron microscope. The multi-layered N-C carrier has a lot of folds, the single atoms of different planes may overlap in two-dimensional projection, presenting a "pseudo-diatomic" phenomenon. Besides, low signal-to-noise ratio or spherical aberration correction residual aberrations may lead to single-atom signal splitting and misidentification as diatomic sites.


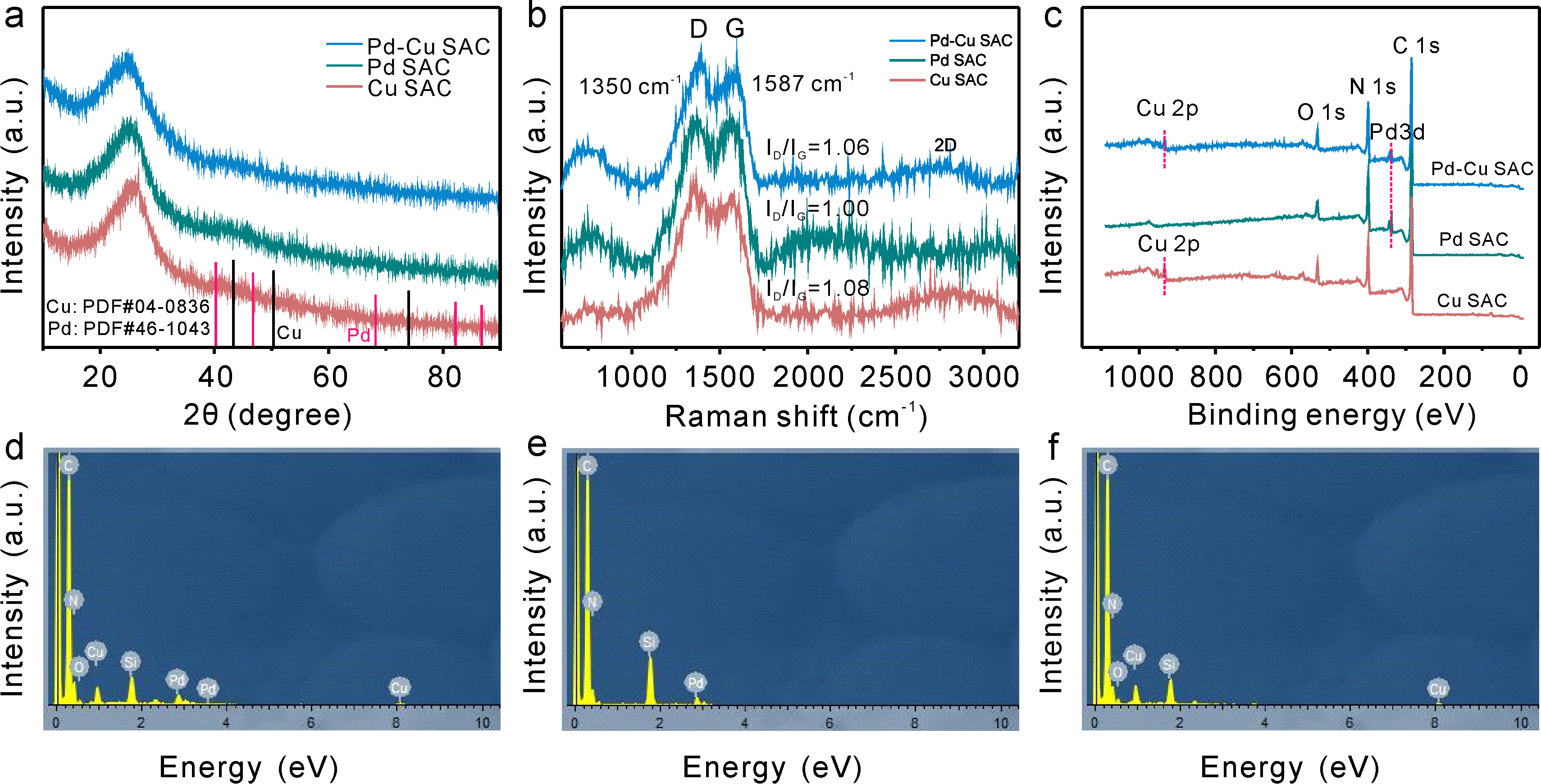


**Figure S3.** Comparison in a) XRD patterns, b) Raman spectra, c) XPS spectra, and d-f) EDS of Pd-Cu DAC, Pd SAC, and Cu SAC catalysts.

There was no diffraction peak of Pd or Cu metals presented in X-ray diffraction (XRD) patterns of Pd-Cu DAC, Pd SAC, and Cu SAC (Figure S3a) which indicated the uniform dispersion of Pd or Cu atoms. Only a widened diffraction peak belonging to the (002) face of graphene appeared at 26°, suggesting that the substrates of Pd-Cu DAC, Pd SAC, and Cu SAC had a graphene-like structure. Furthermore, Raman spectroscopy was employed to compare the relative content of defects in the graphene-like substrates of Pd-Cu DAC, Pd SAC, and Cu SAC samples. As displayed in Raman spectra (Figure S3b), the characteristic D and G peaks of graphene arose at 1350 and 1587 cm^-1^, and their peak intensity ratios (I_D_/I_G_) in Pd-Cu DAC, Pd SAC, and Cu SAC were 1.06, 1.00, and 1.08, respectively. It demonstrated the similar defect contents in these three catalysts.

**Figure S4.** N_2_ adsorption-desorption isotherms of a) Pd-Cu DAC, b) Pd SAC, c) Cu SAC, and d) N-C. The insets show their corresponding aperture distribution.

As depicted in Figure S4a-d, the specific surface areas of Pd-Cu DAC, Pd SAC, Cu SAC, and N-C materials were 275, 363, 223, and 371 m^2^ g^-1^, respectively. Their average pore diameters were 7.8, 7.1, 10.4 and 7.1 nm, respectively.


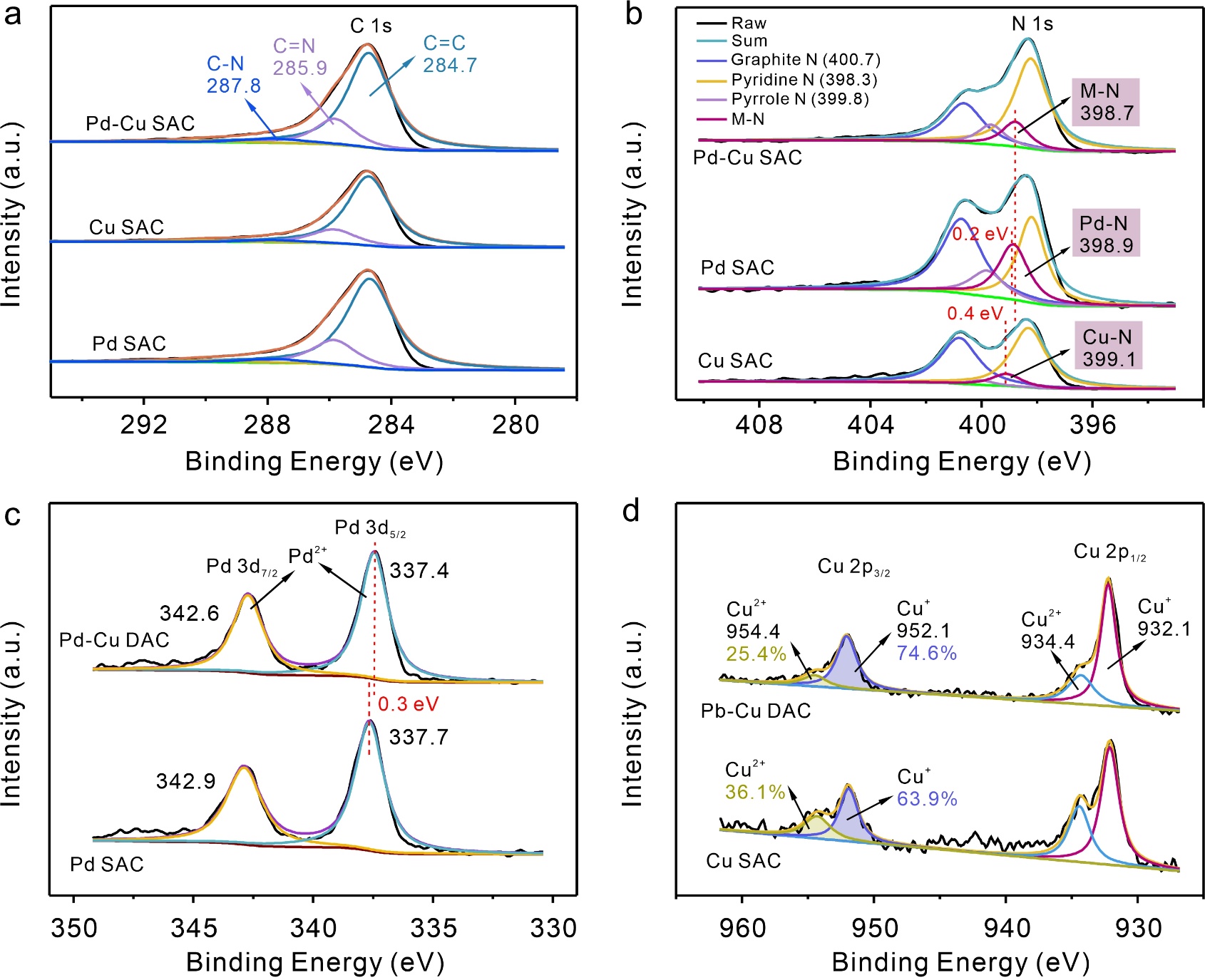


**Figure S5.** Comparison in high-resolution XPS spectra of a) C 1s and b) N 1s among Pd-Cu DAC, Pd SAC, and Cu SAC catalysts. c) High-resolution XPS spectra of Pd 3d in Pd-Cu DAC and Pd SAC. d) High-resolution XPS spectra of Cu 2p in Pd-Cu DAC and Cu SAC.

Figure S5 reflects the high-resolution XPS spectra of C 1s, N 1s, Pd 3d, and Cu 2p in Pd-Cu DAC, Pd SAC, and Cu SAC catalysts. As shown in Figure S5a, the C element in these three catalysts primarily existed in the form of C=C (284.7 eV), C=N (285.9 eV), and C-N (287.8 eV)^[2]^. As depicted in Figure S5b, the XPS spectra of the N element in these three catalysts can be deconvoluted into four peaks, including pyridine-N (398.3 eV), pyrrole-N (399.8 eV), graphite-N (400.7 eV), and M-N (M represents Pd or Cu)^[3]^. Notably, the characteristic peak of M-N in Pd-Cu DAC, Pd SAC, and Cu SAC catalysts located at 398.7, 398.9, and 399.1 eV, respectively. Their difference in peak position certified the chemical state diversity of the N element, which was possibly attributed to the introduction of different metal atoms. In addition, it was discovered that Pd 3d_5/2_ and 3d_7/2_ XPS peaks in Pd-Cu DAC shifted to lower binding energies by 0.3 eV, compared with those in Pd SAC (Figure S5c), which implied that the incorporation of Cu element caused Pd atoms enriching electrons and decreasing their chemical state slightly. Furthermore, as displayed in Figure S5d, Cu 2p_3/2_ and 2p_1/2_ XPS peaks in both Pd-Cu DAC and Cu SAC can be identified into four peaks, which presented at 932.1 and 952.1 eV belonged to Cu^+^, the other two peaks at 934.4 and 954.4 eV belong to Cu^2+^. But the relative content proportion of Cu^+^ in Pd-Cu DAC (74.6%) is apparently higher than that in Cu SAC, which suggests that the participation of Pd atoms promotes the partial conversion of Cu^2+^ into Cu^+^, decreasing the valent state of Cu single atoms.


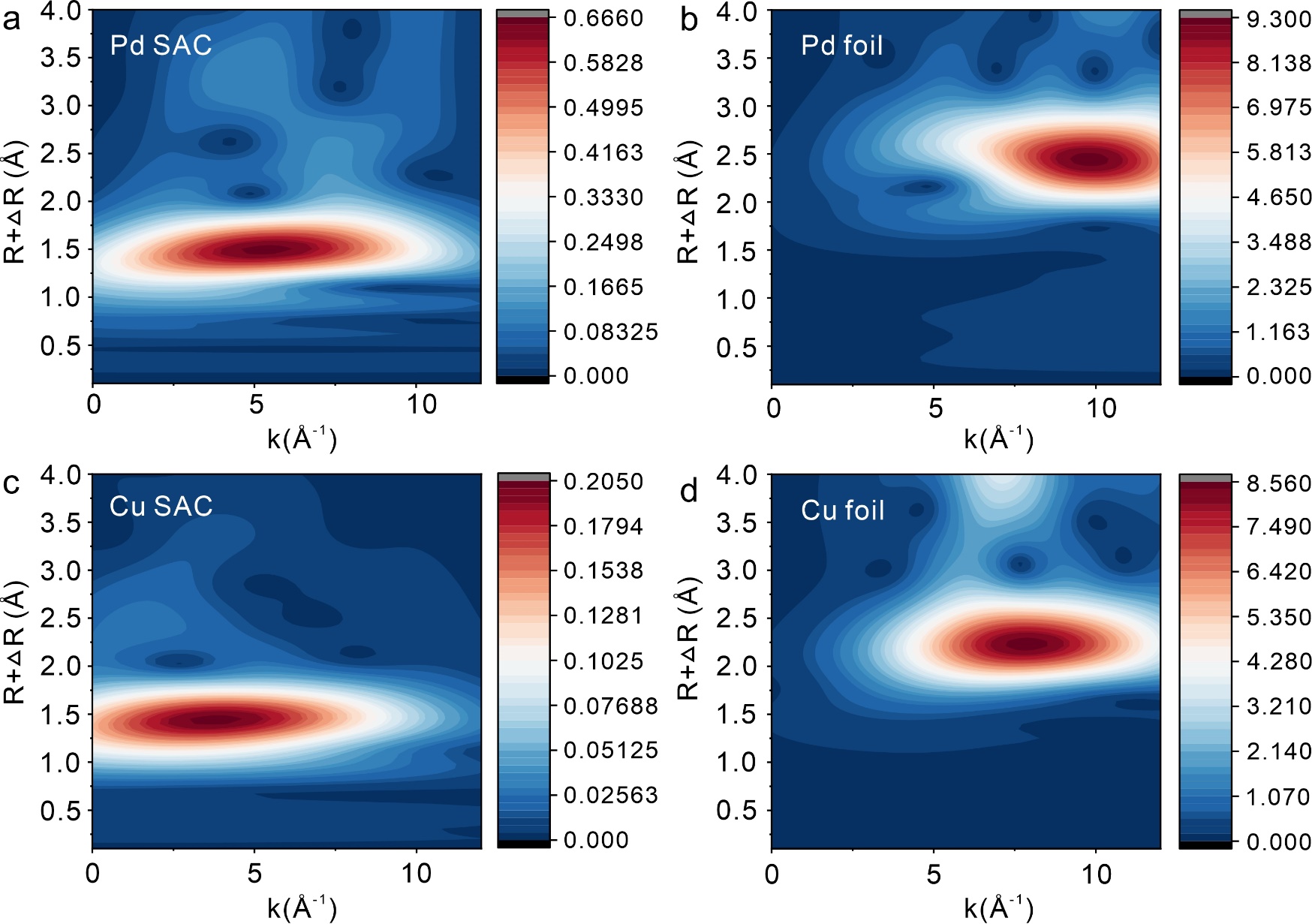


**Figure S6.** Wavelet transformed EXAFS spectra of Pd K-edge in a) Pd SAC and b) Pd foil. Wavelet transformed EXAFS spectra of Cu K-edge in c) Cu SAC and d) Cu foil.


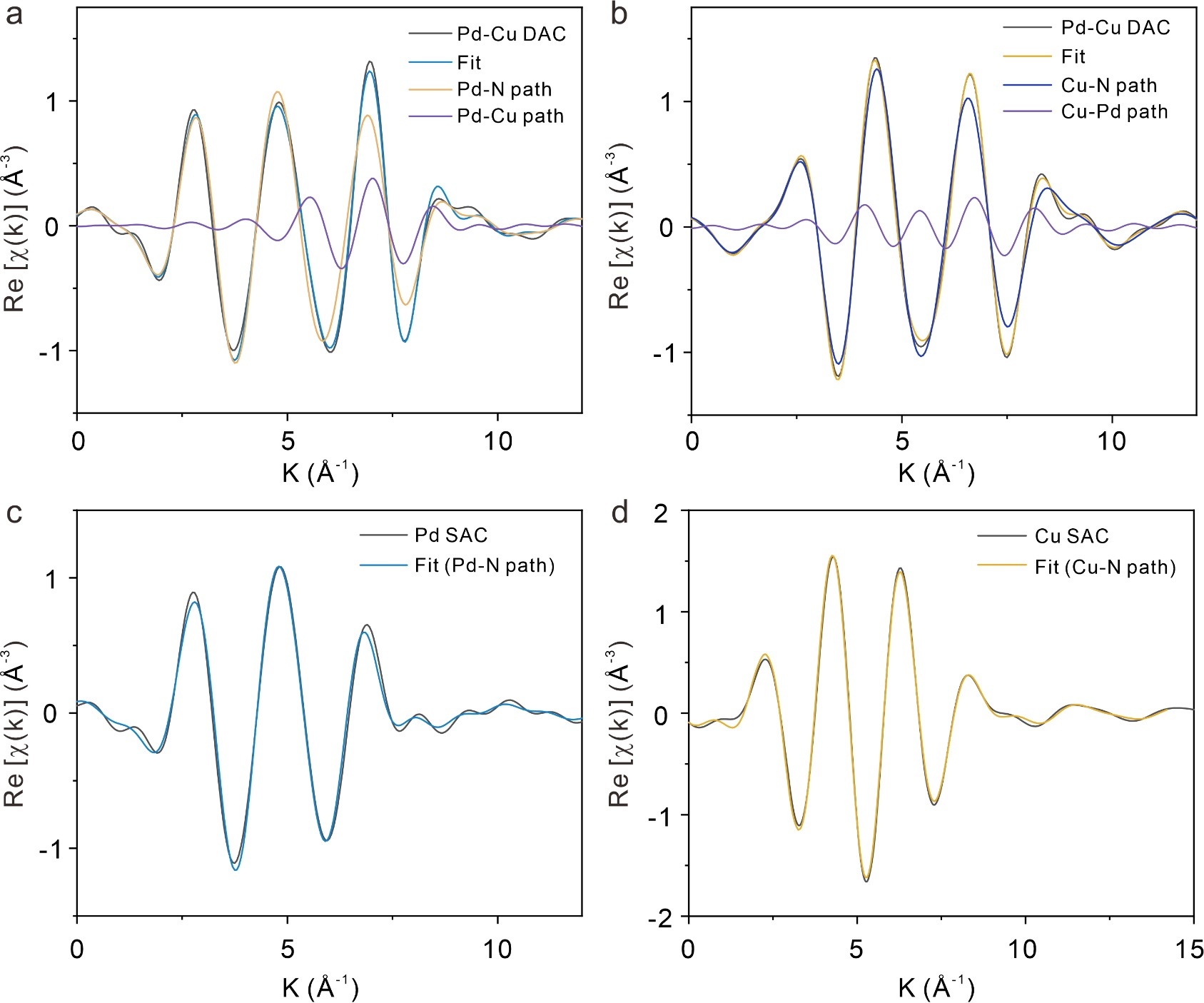


**Figure S7.** k^3^χ(k) oscillation curves corresponding to Figure 2a-d, respectively.


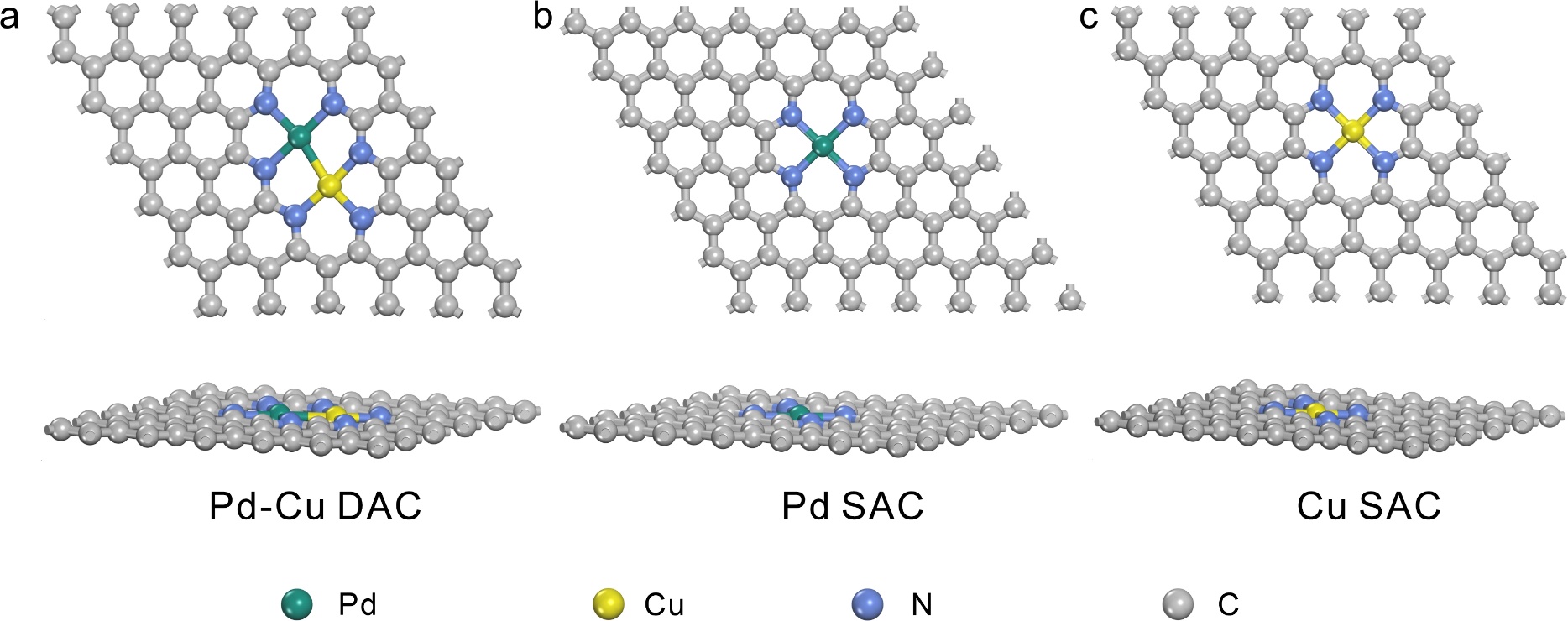


**Figure S8.** The atomic structure models of a) Pd-Cu DAC, b) Pd SAC, and c) Cu SAC catalysts optimized by DFT calculation (side view and top view).


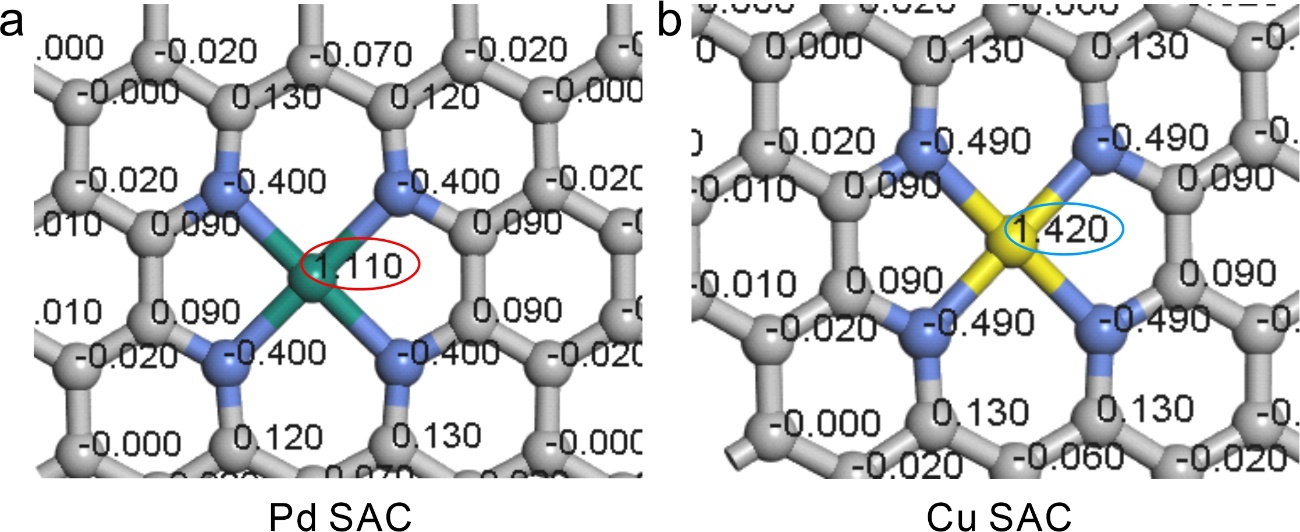


**Figure S9.** Mulliken charge distribution of a) Pd SAC and b) Cu SAC catalysts.


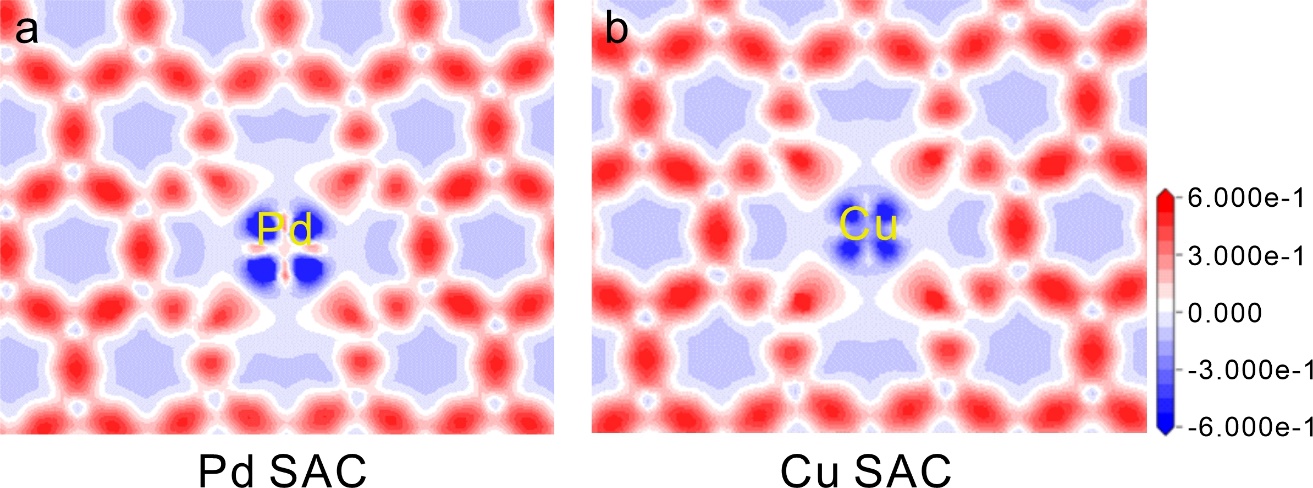


**Figure S10.** Charge density difference plots of the horizontal slices through the Pd (or Cu), N, and C atomic layers on a) Pd SAC and b) Cu SAC catalysts. The blue area represents electron depletion, and the red area represents electron accumulation.

**Figure S11.** Comparisons in PDOS diagrams of Pd (upper half) and Cu (bottom half) single atoms in Pd-Cu DAC, Pd SAC, and Cu SAC.

The PDOS diagrams of Pd and Cu atoms in Pd-Cu DAC, Pd SAC, and Cu SAC were calculated and compared in Figure S11. It is unraveled that the *s*-, *p*-, and *d*-orbital electrons of Pd atoms in Pd-Cu DAC shift to lower energy levels, compared to those in Pd SAC, which suggests that the participation of Cu atoms renders the system more stable. Similarly, by comparing the PDOS diagrams of Cu atoms in Pd-Cu DAC and Cu SAC, it is found that there are new *p*- and *d*-orbital electrons of Cu atoms generated at *ca.* -1.9 eV after Pd atoms incorporation, and the *d*-orbital electrons at the Fermi level slightly shift to 0.4 eV. Meanwhile, the newly generated orbital electrons of Cu atoms well coincide with the *d*-orbital electrons of Pd atoms at *ca.* -1.9 and 0.4 eV, indicating the strong bonding coupling interaction between Pd and Cu atoms. Moreover, the *d*-band center of Pd atoms in Pd-Cu DAC is altered to -3.55 eV from -3.17 eV (in Pd SAC) after the introduction of Cu atoms, and the *d*-band center of Cu atoms in Pd-Cu DAC also has a slight shift to -3.60 eV from -3.50 eV (in Cu SAC) *via* Pd atoms incorporation. The shifts of *d*-band centers of Pd and Cu atoms to lower energy levels are extremely likely favorable for the adsorption and catalysis toward the target Cr(VI).


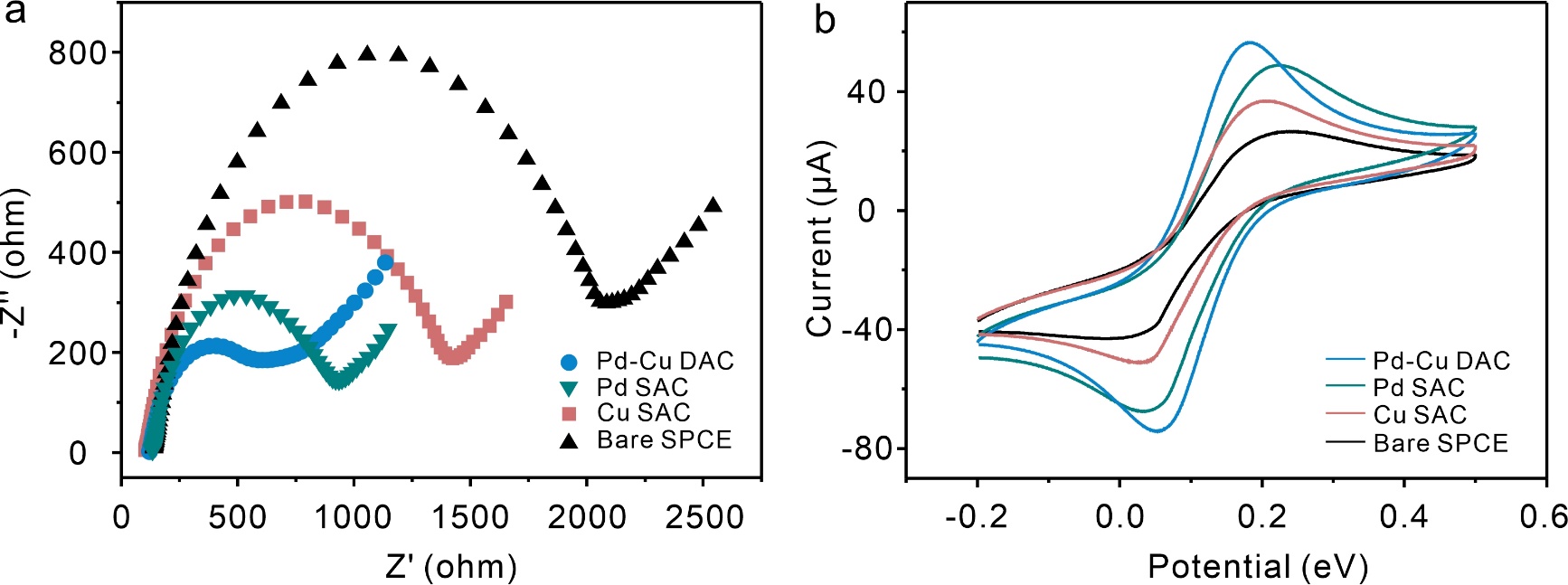


**Figure S12.** Comparison in a) EIS diagrams and b) CV curves of Pd-Cu DAC, Pd SAC, and Cu SAC catalysts modified SPCE tested in the 0.1 M KCl solution containing 5.0 mM K_3_[Fe(CN)_6_]. The potential scan rate was 0.1 V s^-1^.

We selected Fe(CN)_6_^3‑/4‑^ as the ion probes to investigate the electrochemical properties of modified electrodes. The electrochemical impedance spectroscopy (EIS) diagrams in Figure S12a exhibit that the charge-transfer resistances (R_ct_) of Pd-Cu DAC, Pd SAC, Cu SAC, and bare SPCE are *ca.* 650, 900, 1400, and 2150 Ω, respectively. It proves that the interface resistances of modified SPCEs are greatly decreased via the modification of these three catalysts, markedly enhancing the conductivity. The Pd-Cu DAC electrodes possess minimum resistance. What’s more, as depicted in cyclic voltammetry (CV) curves (Figure S12b), the Pd-Cu DAC electrode shows the highest response current toward Fe^3+^/Fe^2+^ redox reactions, compared to other modified electrodes, which implies the fastest electron transfer efficiency of Pd-Cu DAC. Thus, the identical results of EIS and CV certify the superior electronic transfer rate of Pd-Cu DAC catalyst, as well as the relatively inferior conductivity of Pd SAC and Cu SAC.


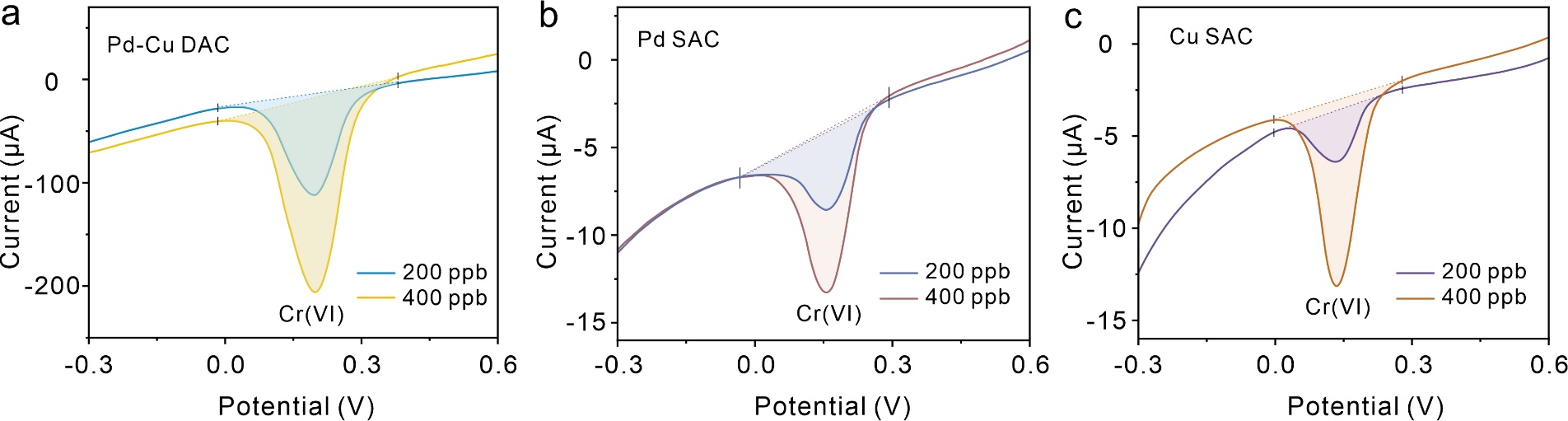


**Figure S13.** LSV current signals of 200 and 400 ppb Cr(VI) measured by a) Pd-Cu DAC, b) Pd SAC, and c) Cu SAC. The shadow areas of different colors represent the corresponding integrated area of peak currents.

Based on the above Equation S5, the number of Cr(VI) transforming into Cr(III) can be confirmed as below:

$n=\frac{Q}{3\times e}=\frac{\int_{E1}^{E2} I\left( E \right)dE}{3\times e\times\nu}$ (S5)

For the Pd-Cu DAC, the values of *n* in the determination of 200 and 400 ppb Cr(VI) were calculated based on Figure S13a as following:

$n(200 ppb)=\frac{1.23\times{10}^{-5}}{3\times{1.6\times10}^{-19} \times0.1}$ $=$ 2.56 $\times$ ${10}^{14}$

$n(400 ppb)=\frac{2.57\times{10}^{-5}}{3\times{1.6\times10}^{-19} \times0.1}$ $=$ 5.35 $\times$ ${10}^{14}$

For the Pd SAC, the values of *n* in the determination of 200 and 400 ppb Cr(VI) were obtained based on Figure S13b, as shown below:

$n(200 ppb)=\frac{4.80\times{10}^{-7}}{3\times{1.6\times10}^{-19} \times0.1}$ $=$ 1.00 $\times$ ${10}^{13}$

$n(400 ppb)=\frac{1.02\times{10}^{-6}}{3\times{1.6\times10}^{-19} \times0.1}$ $=$ 2.125 $\times$ ${10}^{13}$

For the Cu SAC, the values of *n* in the determination of 200 and 400 ppb Cr(VI) were calculated based on Figure S13c, as shown below:

$n(200 ppb)=\frac{3.91\times{10}^{-7}}{3\times{1.6\times10}^{-19} \times0.1}$ $=$ 8.15 $\times$ ${10}^{12}$

$n(400 ppb)=\frac{9.88\times{10}^{-7}}{3\times{1.6\times10}^{-19} \times0.1}$ $=$ 2.06 $\times$ ${10}^{13}$

For the actual reaction time (*t*), it can be obtained according to the above Equation S3:

For Pd-Cu DAC, $t=\frac{E}{v}=\frac{0. 40 V}{0.1 v/s}$ = 4.0 s

For Pd SAC, $t=\frac{E}{v}=\frac{0.33 V}{0.1 v/s}$ = 3.3 s

For Cu SAC, $t=\frac{E}{v}=\frac{0.28 V}{0.1 v/s}$ = 2.8 s

In this work, the electrode was modified with 10 μL of the suspension solution containing catalysts with a concentration of 1 mg mL^-1^. It is worth noting that the relative content of Pd and Cu elements in the Pd-Cu DAC catalyst are 3.0 wt% and 3.2 wt%, respectively, and the molar mass of the Pd atom is higher than that of the Cu atom. Thus, the number of Pd atoms in Pd-Cu DAC material is less than that of Cu atoms. It is assumed that all Pd atoms existed in pairs with Cu atoms in Pd-Cu DAC. Then, the number of active sites on the electrode surface (*N*) was evaluated based on the above Equation S6:

$N=\frac{m}{M}\times N_{A}$ (S6)

For Pd-Cu DAC, $N=\frac{1mg/mL 10 L3.0\%}{106.4 g/mol}\times6.02\times{10}^{23}=1.70\times{10}^{15}$

For Pd SAC, $N=\frac{1mg/mL10L4.5\%}{106.4 g/mol}\times6.02\times{10}^{23}=2.55\times{10}^{15}$

For Cu SAC, $N=\frac{1mg/mL10L5.0\%}{63.5 g/mol}\times6.02\times{10}^{23}=4.74\times{10}^{15}$

Therefore, according to the above results, the values of TOF in the detection of 200 and 400 ppb Cr(VI) can be calculated as below:

For Pd-Cu DAC catalyst:

$$TOF(200 ppb)=\frac{n}{N\times t}=\frac{2.56\times{10}^{14}}{1.70\times{10}^{15}\times4.0}\approx0.038 (s^{-1})$$

$$TOF(400 ppb)=\frac{n}{N\times t}=\frac{5.35\times{10}^{14}}{1.70\times{10}^{15}\times4.0}\approx0.079 (s^{-1})$$

For Pd SAC catalyst:

$$TOF(200 ppb)=\frac{n}{N\times t}=\frac{1.00\times{10}^{13}}{2.55\times{10}^{15}\times3.3}\approx0.0012 (s^{-1})$$

$$TOF(400 ppb)=\frac{n}{N\times t}=\frac{2.125\times{10}^{13}}{2.55\times{10}^{15}\times3.3}\approx0.0025 (s^{-1})$$

For Cu SAC catalyst:

$$TOF(200 ppb)=\frac{n}{N\times t}=\frac{8.15\times{10}^{12}}{4.74\times{10}^{15}\times2.8}\approx0.00061 (s^{-1})$$

$$TOF(400 ppb)=\frac{n}{N\times t}=\frac{2.06\times{10}^{13}}{4.74\times{10}^{15}\times2.8}\approx0.00155 (s^{-1})$$

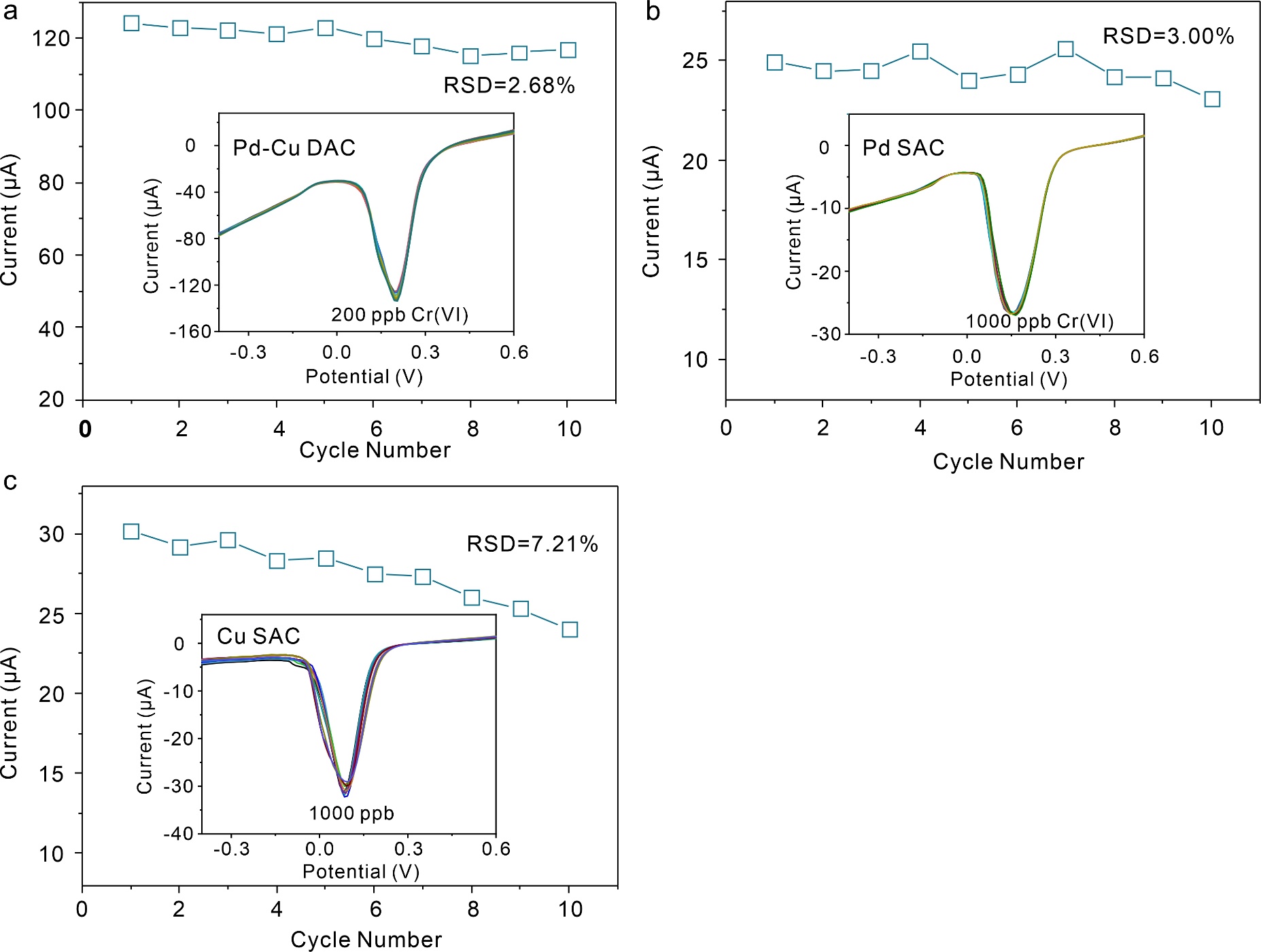


**Figure S14.** The stability experiments of a) Pd-Cu DAC, b) Pd SAC, and c) Cu SAC electrodes in the repeated determination of Cr(VI). The panels are their corresponding LSV response curves. The test conditions are the same as those in Figure 3.


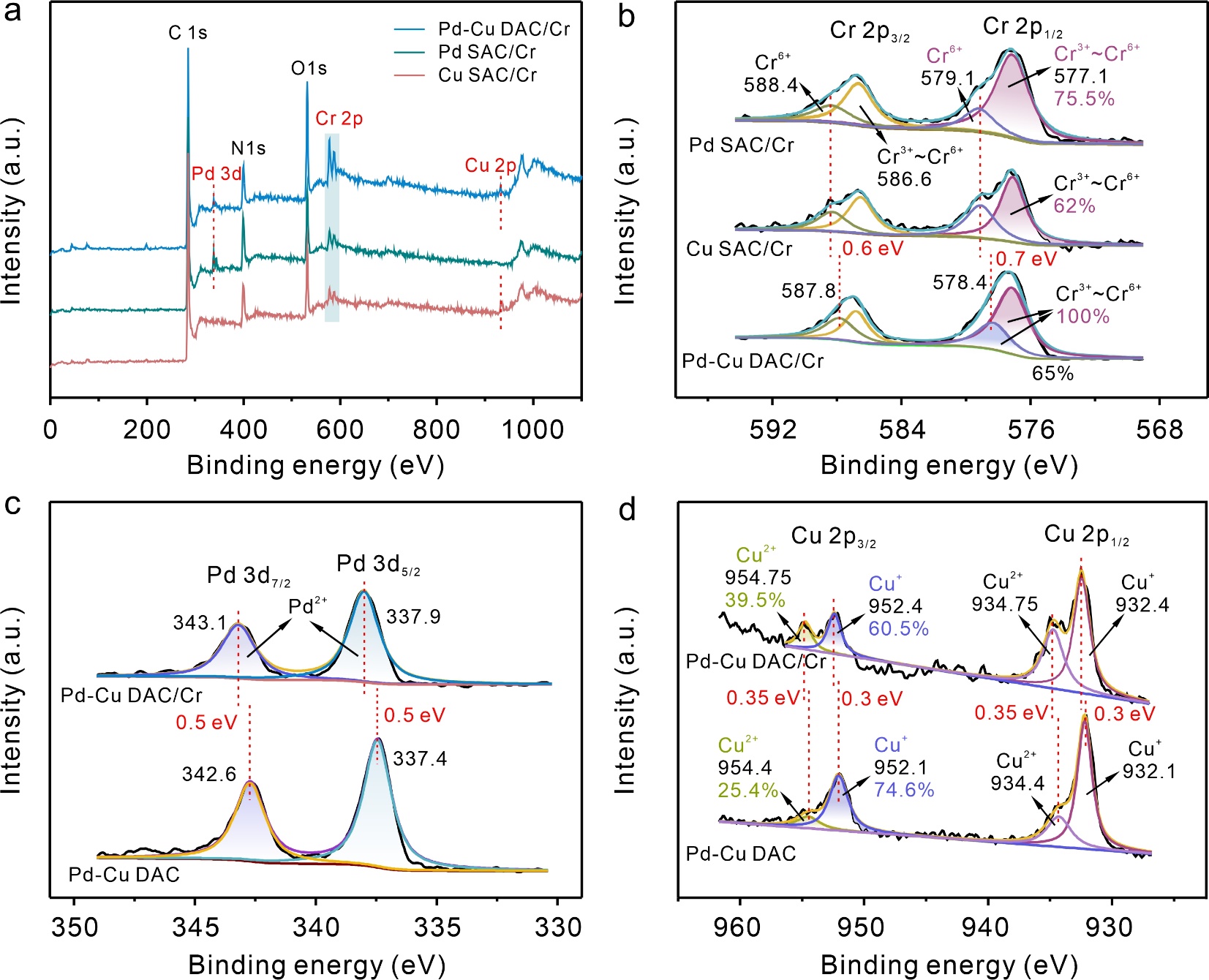


**Figure S15.** a) XPS spectra of Pd-Cu DAC, Pd SAC, and Cu SAC catalysts after Cr(VI) adsorption. b) Comparison in high-resolution XPS spectra of Cr 2p in Pd-Cu DAC/Cr, Pd SAC/Cr, and Cu SAC/Cr samples. Comparisons in high-resolution XPS spectra of c) Pd 3d and d) Cu 2p before and after Pd-Cu DAC adsorbing Cr(VI).

It is observed that after adsorbing Cr(VI), the characteristic peaks of Cr 2p were presented in XPS spectra of Pd-Cu DAC/Cr, Pd SAC/Cr, and Cu SAC/Cr samples (Figure S15a). Via analyzing the high-resolution XPS (HR-XPS) spectra of Cr 2p (Figure S15b), it can be found that in Pd SAC/Cr and Cu SAC/Cr samples, the HR-XPS spectra of Cr 2p_1/2_ and Cr 2p_3/2_ can be deconvoluted into four characteristic peaks located at 577.1, 579.1, 586.6, and 588.4 eV, respectively. The peaks at 579.1 and 588.4 eV are assigned into Cr^6+^, and the other two peaks at 577.1 and 586.6 eV are recognized to Cr^3+^ ⁓ Cr^6+^, which reflects that when adsorbed on Pd SAC and Cu SAC catalysts, a part of Cr(VI) ions are reduced immediately and the valent state is decreased into +3 ⁓ +6. The relative proportions of the reduced chromium ions are 75.5% and 62.0% on Pd SAC and Cu SAC, respectively. Similarly, the reduction of chromium ions also can be discovered in the HR-XPS spectra of Cr 2p in the Pd-Cu DAC/Cr sample. Four characteristic peaks arise at 577.1, 578.4, 586.6, and 587.8 eV, respectively. The peaks at 578.4 and 587.8 eV are shifted towards low binding energy, compared with that in Pd SAC/Cr and Cu SAC/Cr, suggesting that the valent state of the adsorbed chromium ions is lower than +6. It is demonstrated that all the chromium ions adsorbed on Pd-Cu DAC are reduced and transformed to Cr^3+^ ⁓ Cr^6+^, while chromium ions adsorbed on Pd SAC and Cu SAC are partially reduced, which further indicates the glorious catalytic ability of Pd-Cu DAC to reduce Cr(VI).

In addition, Figure S15c shows the HR-XPS spectra of Pd 3d in Pd-Cu DAC before and after adsorbing Cr(VI). The two characteristic peaks of Pd 3d_7/2_ and Pd 3d_5/2_ belonging to Pd^2+^ moved towards the high binding energy by 0.5 eV after interaction with Cr(VI). The increased binding energy of Pd^2+^ in Pd-Cu DAC/Cr implies the loss of electrons. What’s more, when comparing the HR-XPS spectra of Cu 2p_1/2_ and Cu 2p_3/2_ in Pd-Cu DAC before and after adsorbing Cr(VI) (Figure S15d), it is clearly found that the integral area ratio of characteristic peaks assigned to Cu^+^ (932.4 and 952.4 eV) in Pd-Cu DAC/Cr is obviously decreased to 60.5% from 74.6% (Pd-Cu DAC). Besides, the integral area ratio of characteristic peaks assigned to Cu^2+^ (934.75 and 954.75 eV) in Pd-Cu DAC/Cr is increased to 39.5% from 25.4% (Pd-Cu DAC), and their corresponding peak position shifts to high binding energy by 0.35 eV. The increased relative content of Cu^2+^ and positive shift of peak position demonstrate that the valence state of Cu element in Pd-Cu DAC is increased, accompanied by the loss of electrons. The above analysis suggests that both the Pd and Cu atoms in Pd-Cu DAC serve as active sites to interact with the adsorbate chromium ions.


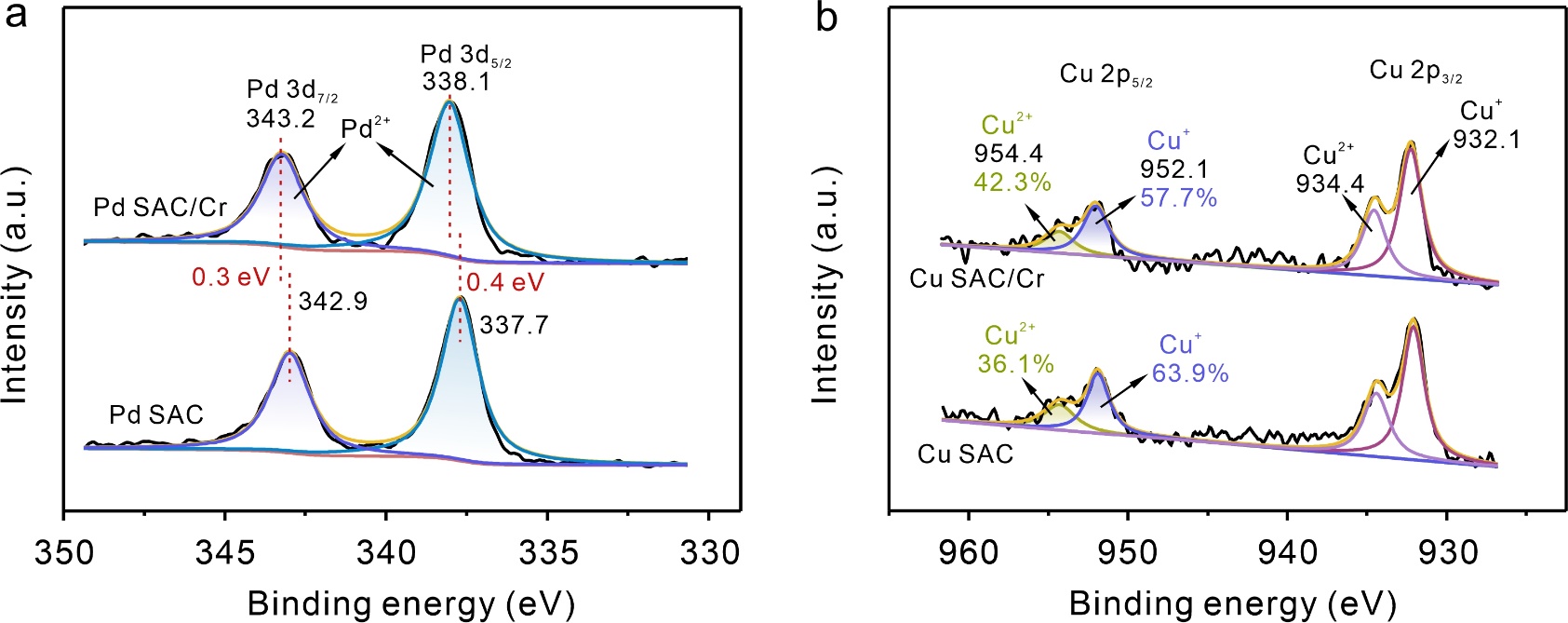


**Figure S16.** a) Comparison in HR-XPS spectra of Pd 3d in Pd SAC before and after adsorbing Cr(VI). b) Comparison in HR-XPS spectra of Cu 2p in Cu SAC before and after adsorbing Cr(VI).

Via analyzing the HR-XPS spectra of Pd 3d (Figure S16a), it is discovered that the characteristic peak of Pd 3d_7/2_ and Pd 3d_5/2_ in Pd SAC/Cr shift to a higher binding energy by 0.3 eV in comparison with that in Pd SAC, unraveling the slightly elevated chemical state of Pd sing atoms after interaction with Cr(VI). In addition, as depicted in HR-XPS spectra of Cu 2p_5/2_ and Cu 2p_3/2_ in Cu SAC/Cr and Cu SAC (Figure S16b), it is found that the position of characteristic peaks assigned to Cu^+^ and Cu^2+^ almost remain unchanged before and after adsorbing Cr(VI), however, their integral area and relative content of Cu^2+^ slightly increase, indicating the increased valence state of Cu single atoms and the partial transformation of Cu^+^ to Cu^2+^. These prove the strong chemical interaction between Pd SAC or Cu SAC with Cr(VI).


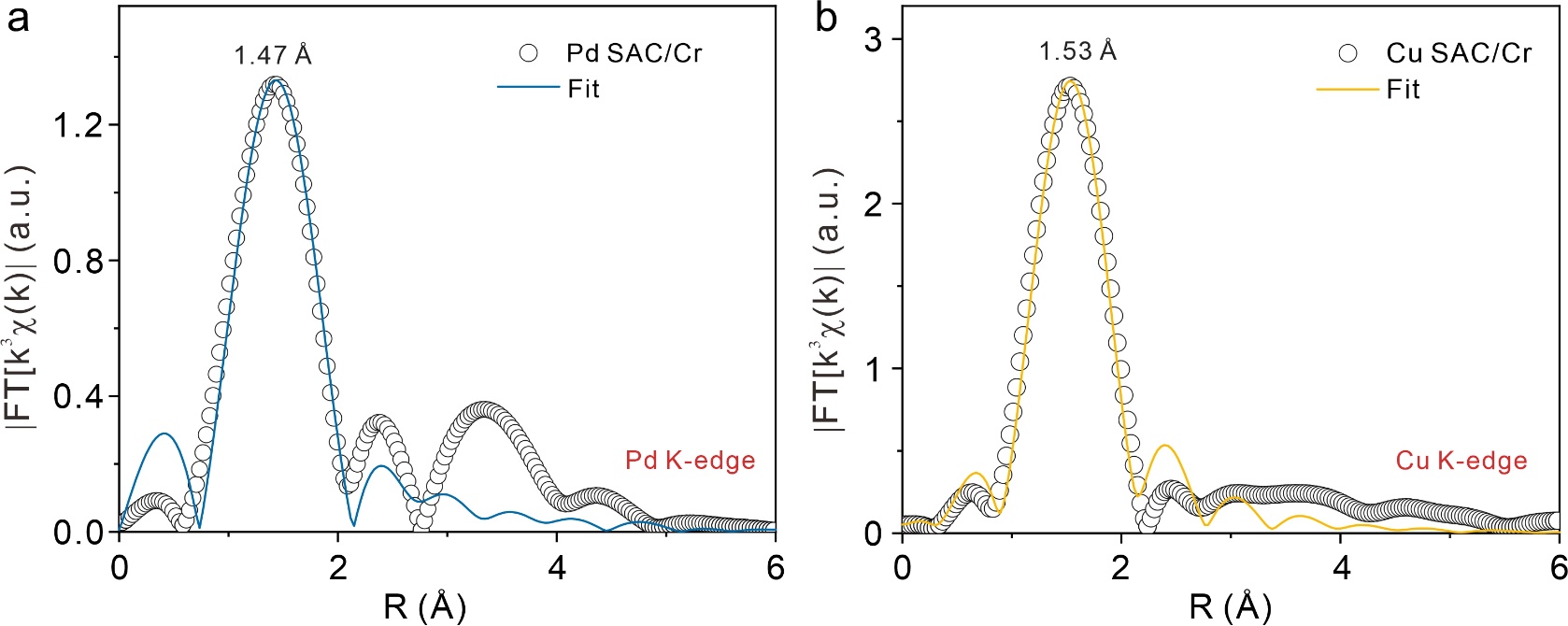


**Figure S17.** a) Pd K-edge EXAFS spectra (R space, k^3^-weighted) of Pd SAC/Cr after Fourier transform and the fitting curve. b) Cu K-edge EXAFS spectra (k^3^-weighted) of Cu SAC/Cr after Fourier transform and the fitting curve.

As shown in the Pd K-edge EXAFS spectrum (R space) of Pd SAC/Cr after the Fourier transform (Figure S17a), a main signal appears at 1.47 Å. After fitting this spectrum, it is revealed that there is just one scattering path of Pd-N presented in the first shell coordination of the Pd single atom. The bond lengths of Pd-N were 1.98 Å with a coordination number of 3.5.

As depicted in the Cu K-edge EXAFS spectra of Cu SAC/Cr processed via Fourier transform (Figure S17b), a main signal peak arises at 1.53 Å. After the fitting analysis, it was unraveled that only the scattering path of Cu-N appeared in the first shell coordination environment of Cu single atoms. The distance of the Cu-N scattering path is 1.95 Å with a coordination number of 3.9.


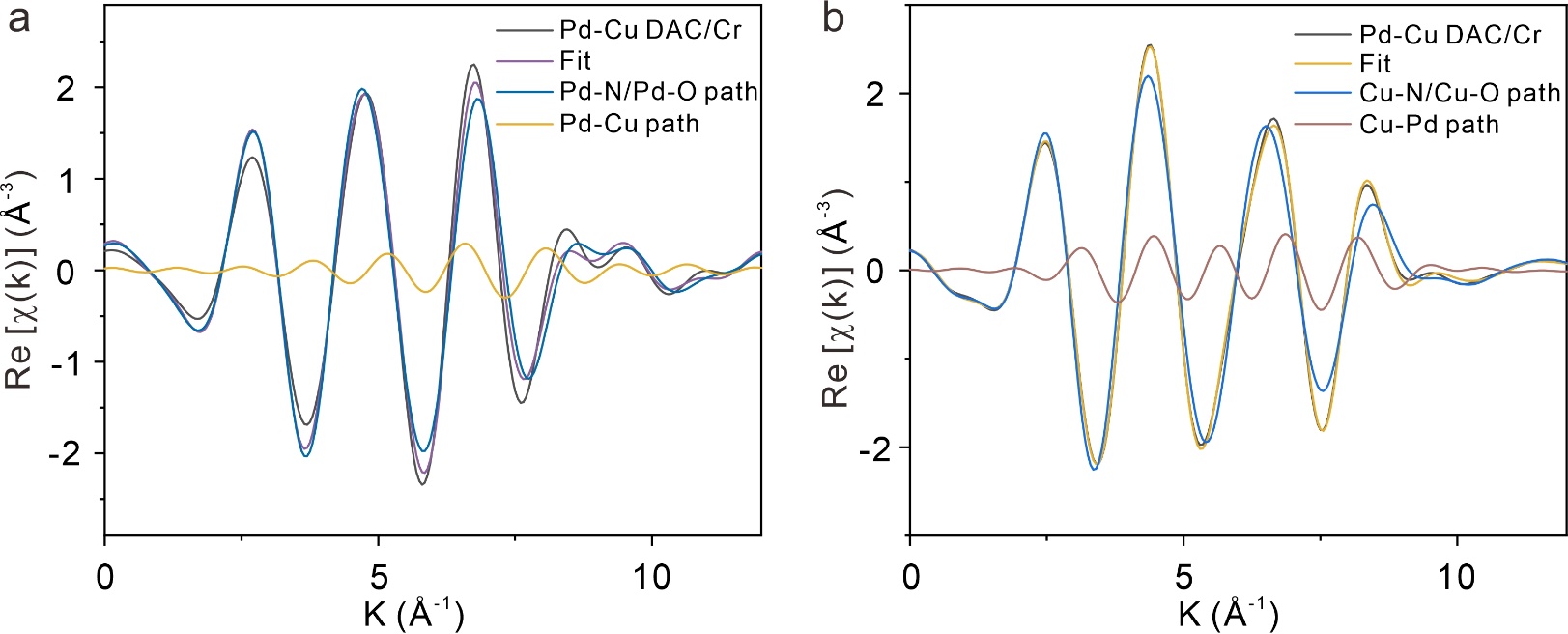


**Figure S18.** a) The corresponding k^3^χ(k) oscillation curves of Figure 4c. b) The corresponding k^3^χ(k) oscillation curves of Figure 4d. The good overlap between the original data and their fitting curves implies the accuracy and reliability of data analysis.


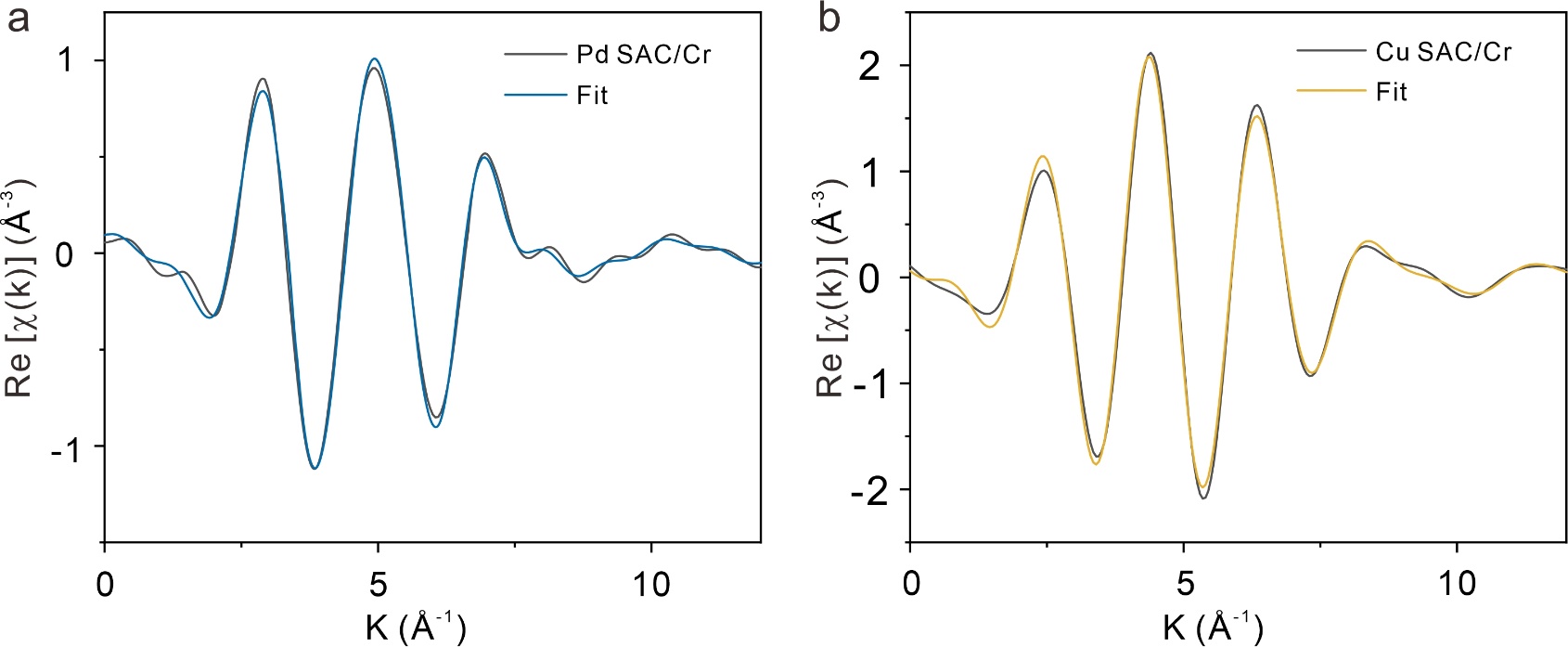


**Figure S19.** The corresponding k^3^χ(k) oscillation curves of Figure S17. The good overlap between the original data and their fitting curves implies the accuracy and reliability of data analysis.


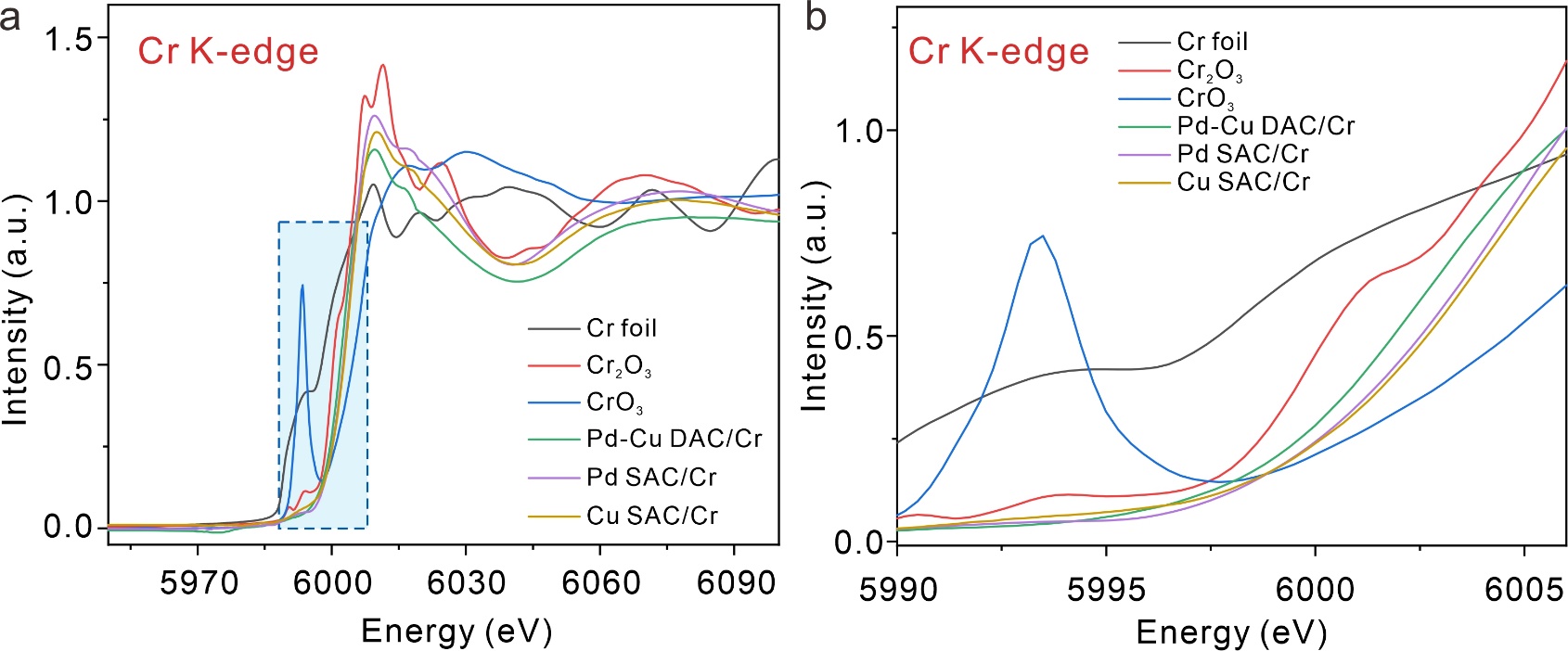


**Figure S20.** a) Comparison in the normalized Cr K-edge XANES spectra (k^2^-weighted) in Pd-Cu DAC/Cr, Pd SAC/Cr, and Cu SAC/Cr samples. b) Amplified plot corresponding to the blue background area in Figure S20a.


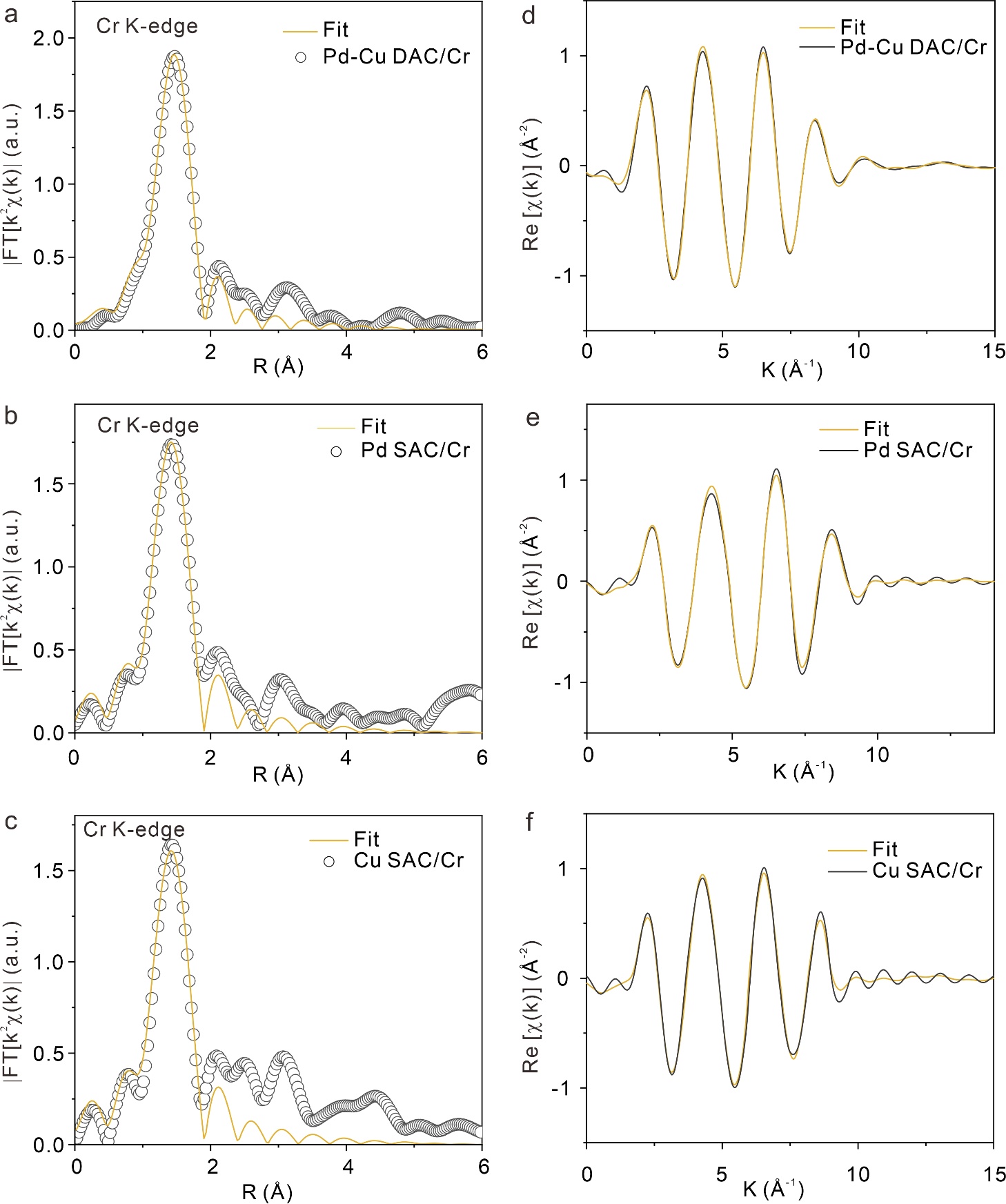


**Figure S21.** Cr K-edge EXAFS spectra (R space, k^2^-weighted) and their fitting curves in a) Pd-Cu DAC, b) Pd SAC/Cr, and c) Cu SAC/Cr samples after Fourier transform without correcting for scattering phase shift. d-f) The k^2^χ(k) oscillation curves corresponding to Figure S20a-c, respectively. S20


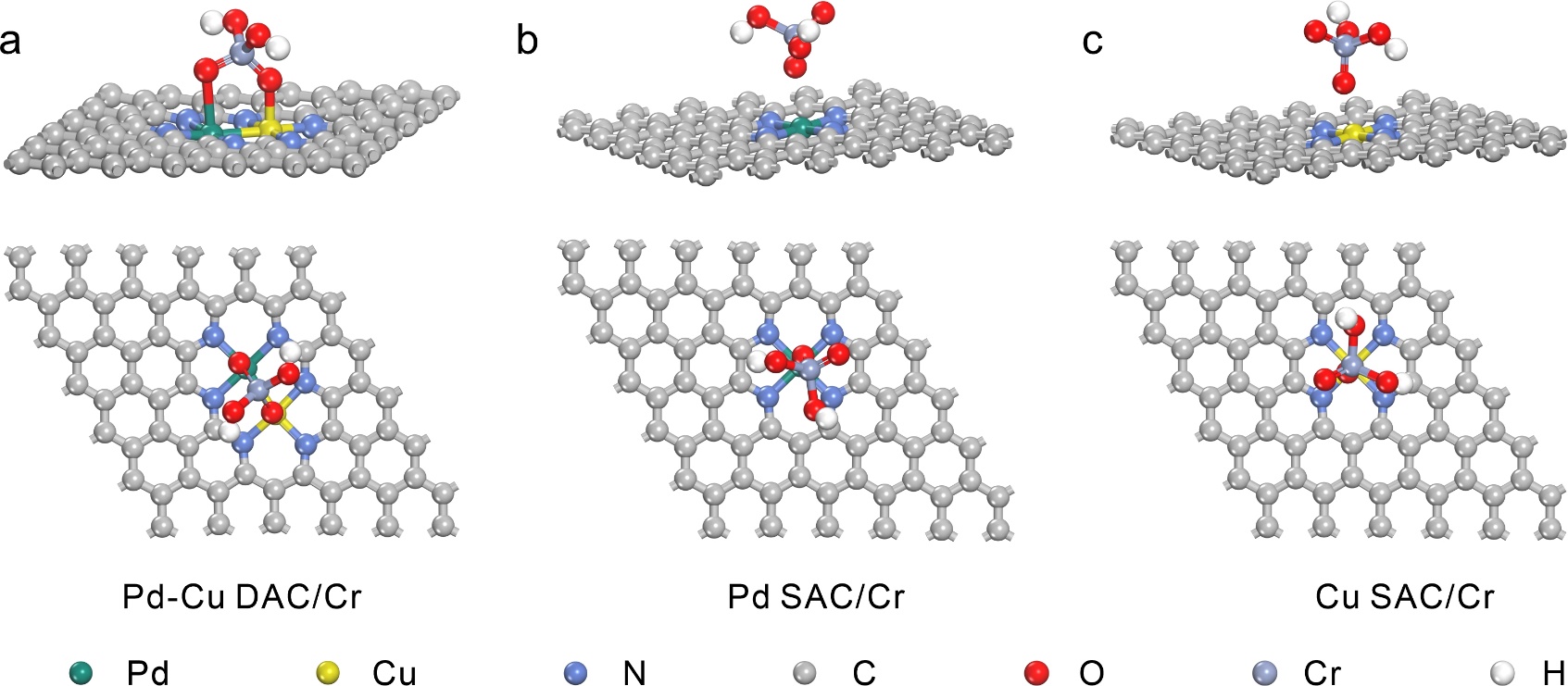


**Figure S22.** Side view and top view of the optimal interaction configurations of a) Pd-Cu DAC/Cr, b) Pd SAC/Cr, and c) Cu SAC/Cr.


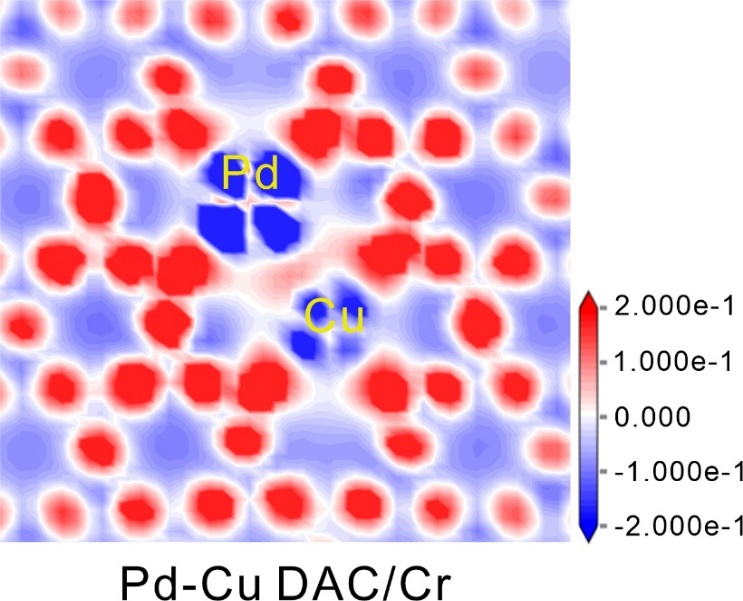


**Figure S23.** Charge density difference plot of the horizontal slices through the Pd, Cu, and N atomic layers on Pd-Cu DAC/Cr. The blue area represents electron depletion, and the red area represents electron accumulation.


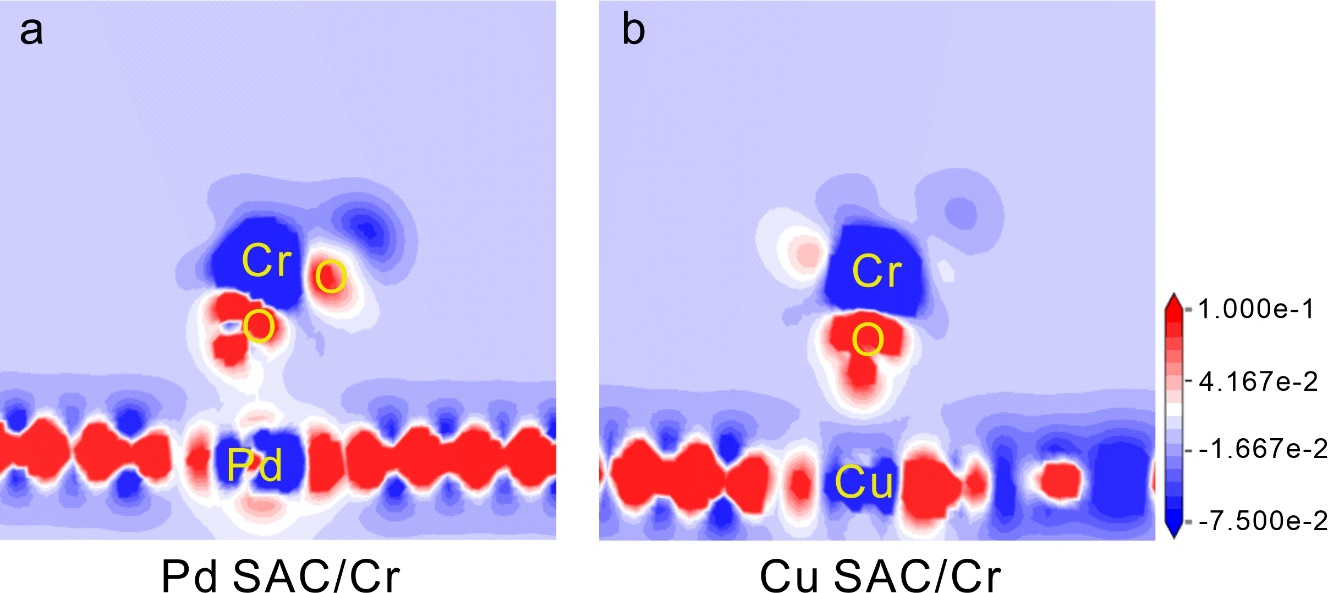


**Figure S24.** Charge density difference plots of the vertical slices through the Pd (or Cu), Cr, and O atomic layers on a) Pd SAC/Cr and b) Cu SAC/Cr. The blue area represents electron depletion, and the red area represents electron accumulation.


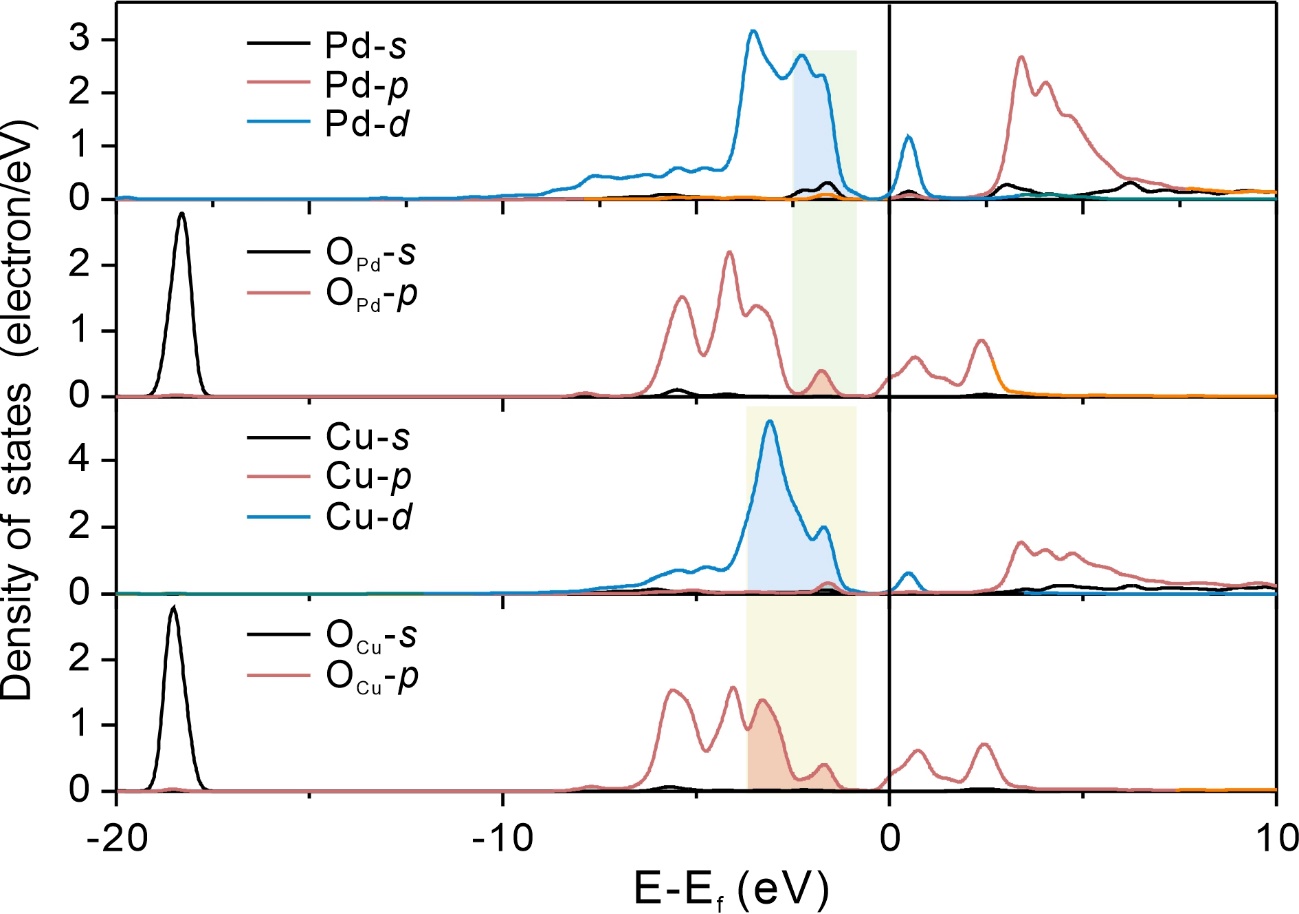


**Figure S25.** PDOS diagrams of Pd, Cu, and O atoms in Pd-Cu DAC/Cr.


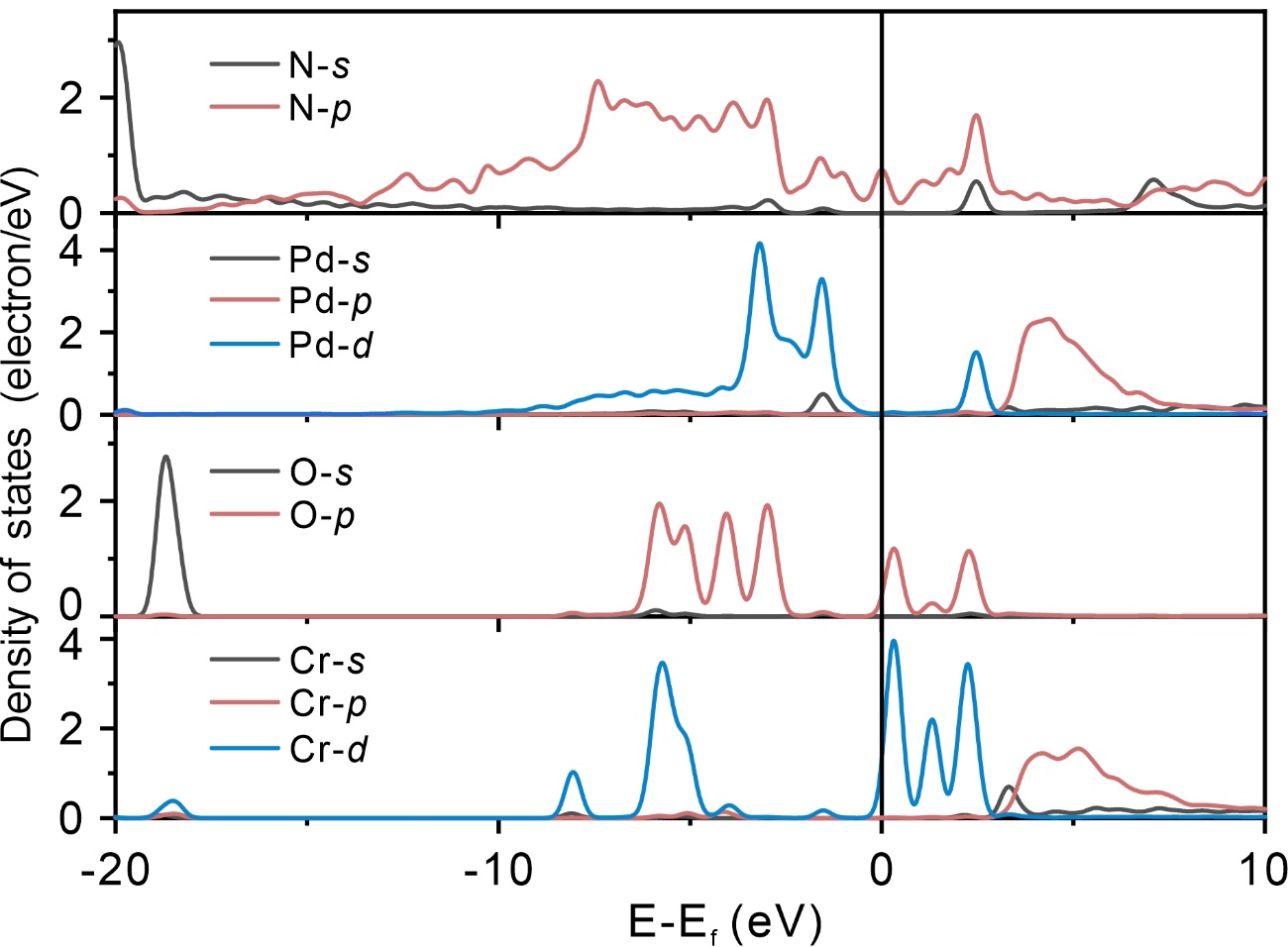


**Figure S26.** PDOS diagrams of Pd, N, Cr, and O atoms in Pd SAC/Cr.


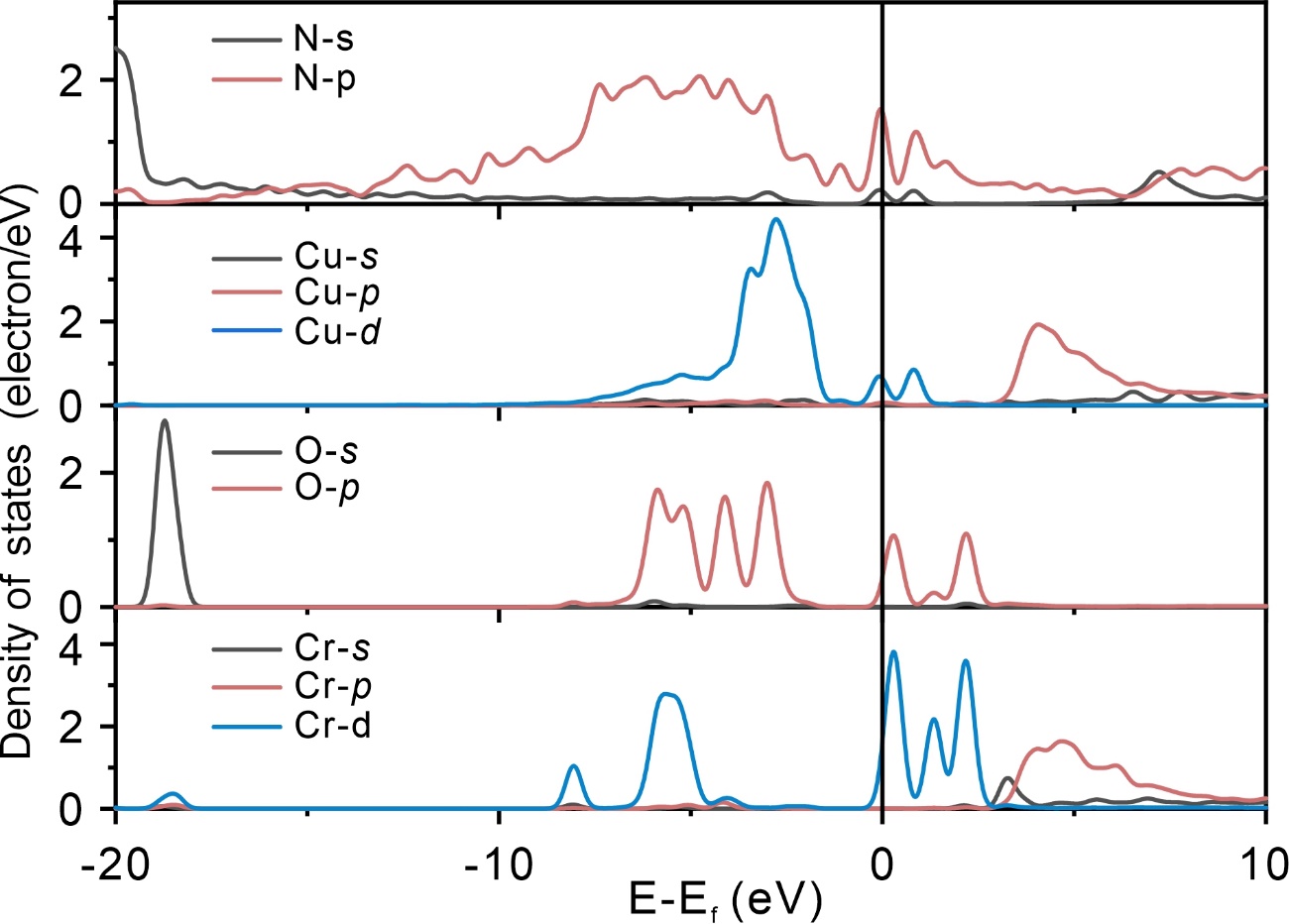


**Figure S27.** PDOS diagrams of Cu, N, Cr, and O atoms in Cu SAC/Cr.


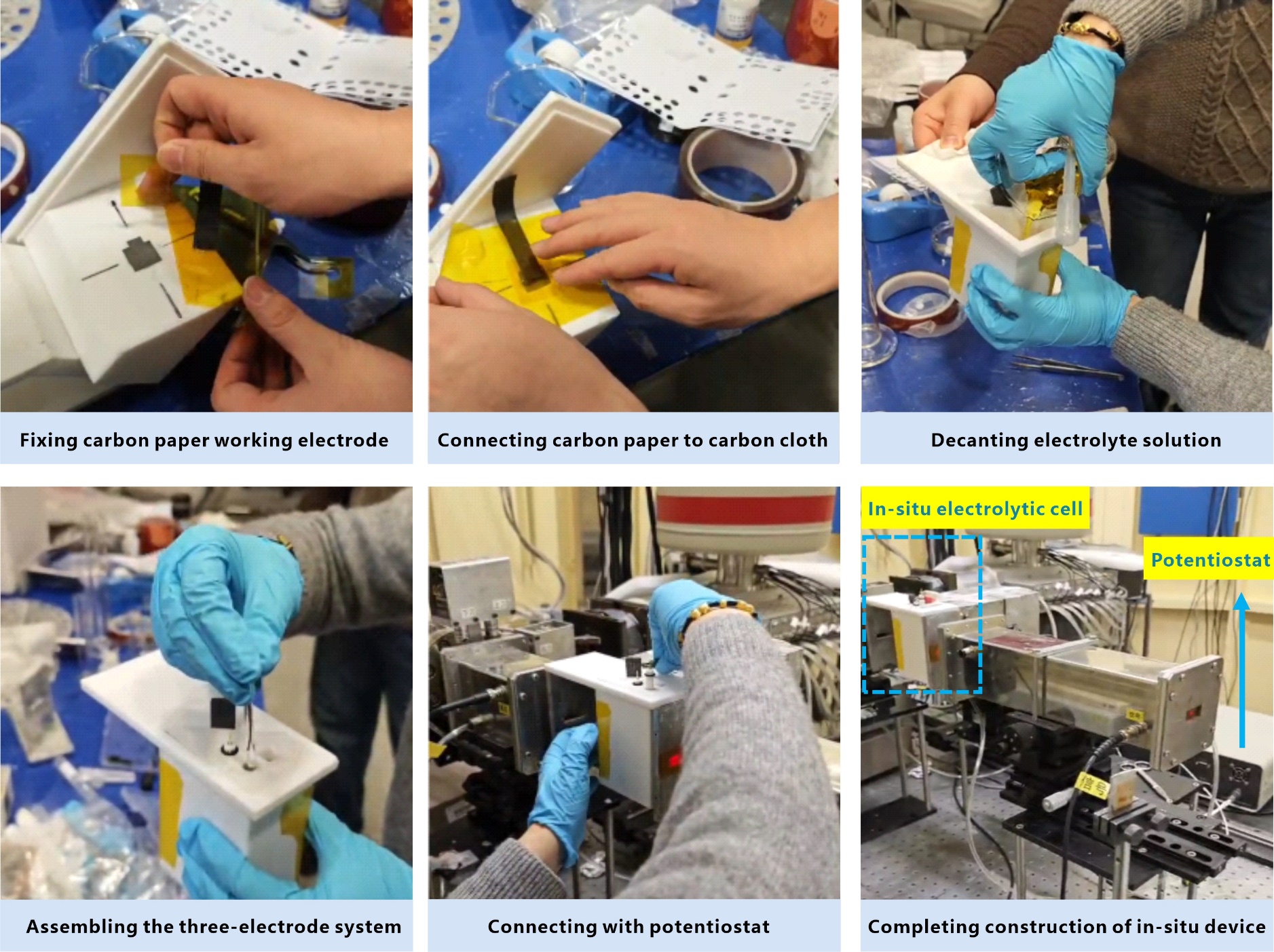


**Figure S28.** Operation procedure of in-situ XAFS technique combined with electrochemical tests.


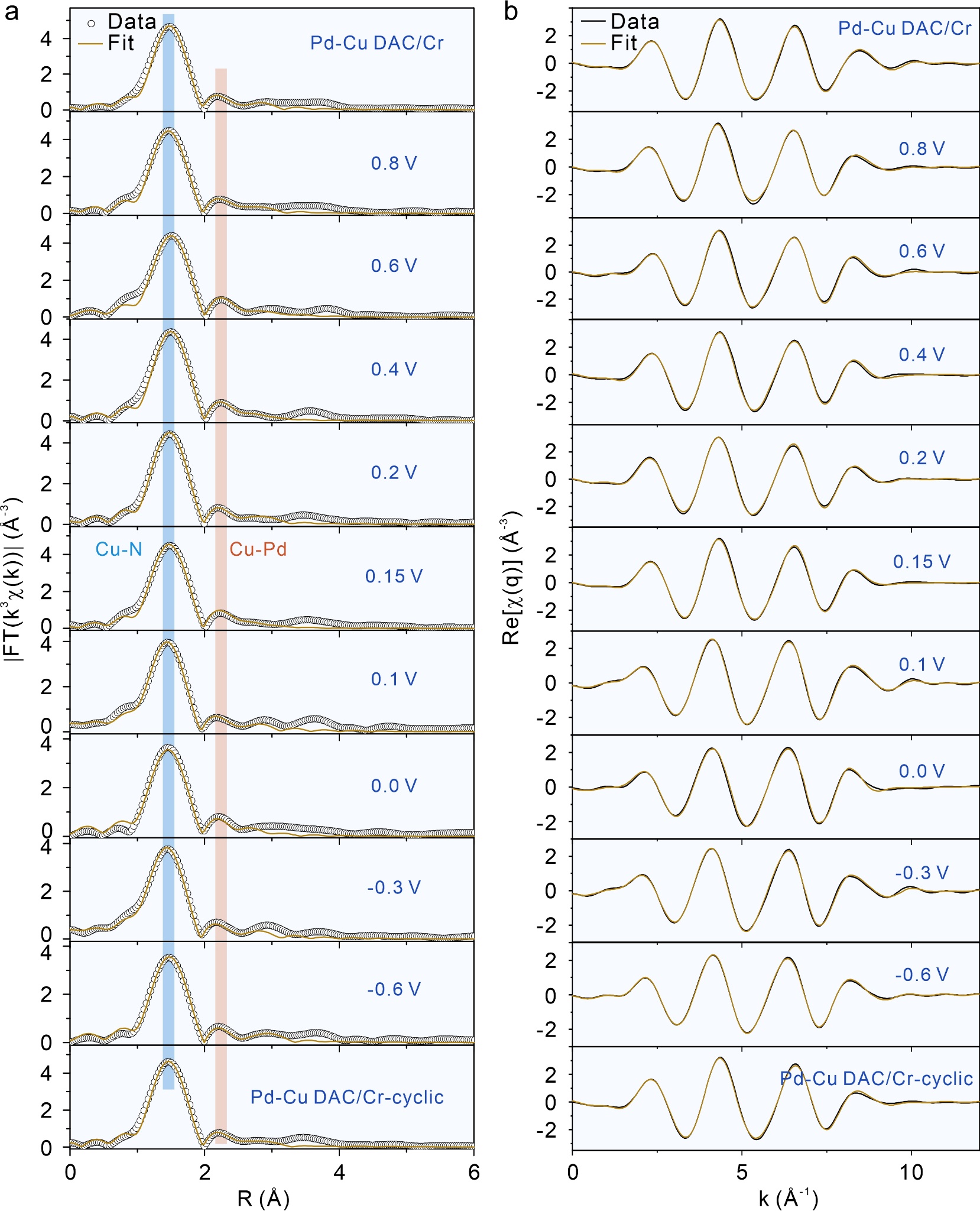


**Figure S29.** a) In-situ EXAFS spectra of Cu K-edge in Pd-Cu DAC/Cr with different applied potentials after Fourier transform and the corresponding fitting curves. b) The corresponding k^3^χ(k) oscillation curves of Figure S29a. The good overlap between the original data and their fitting curves proves the accuracy and reliability of data analysis.


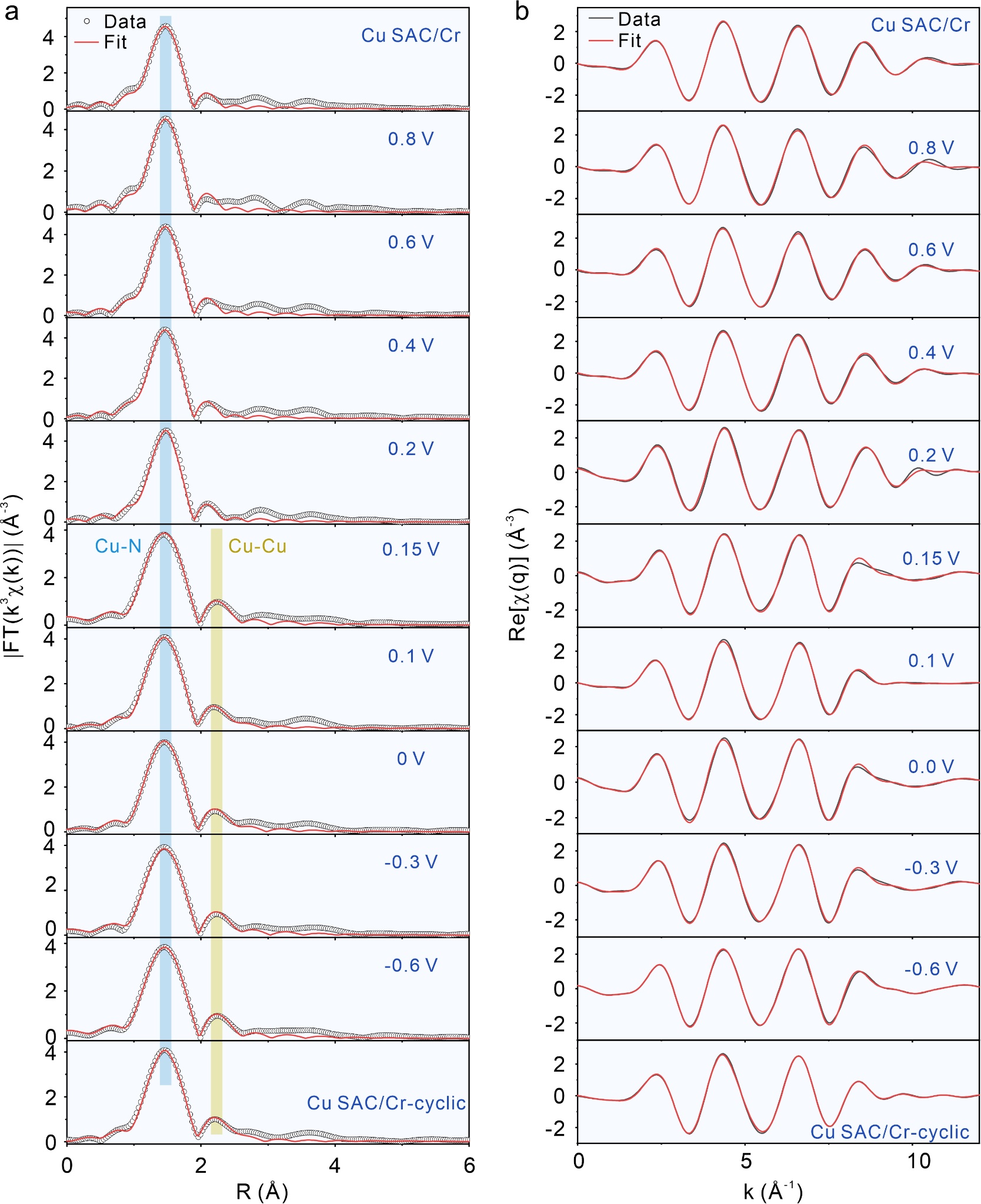


**Figure S30.** a) In-situ EXAFS spectra of Cu K-edge in Cu SAC/Cr with different applied potentials after Fourier transform and the corresponding fitting curves. b) The corresponding k^3^χ(k) oscillation curves of Figure S30a. The good overlap between the original data and their fitting curves proves the accuracy and reliability of data analysis.


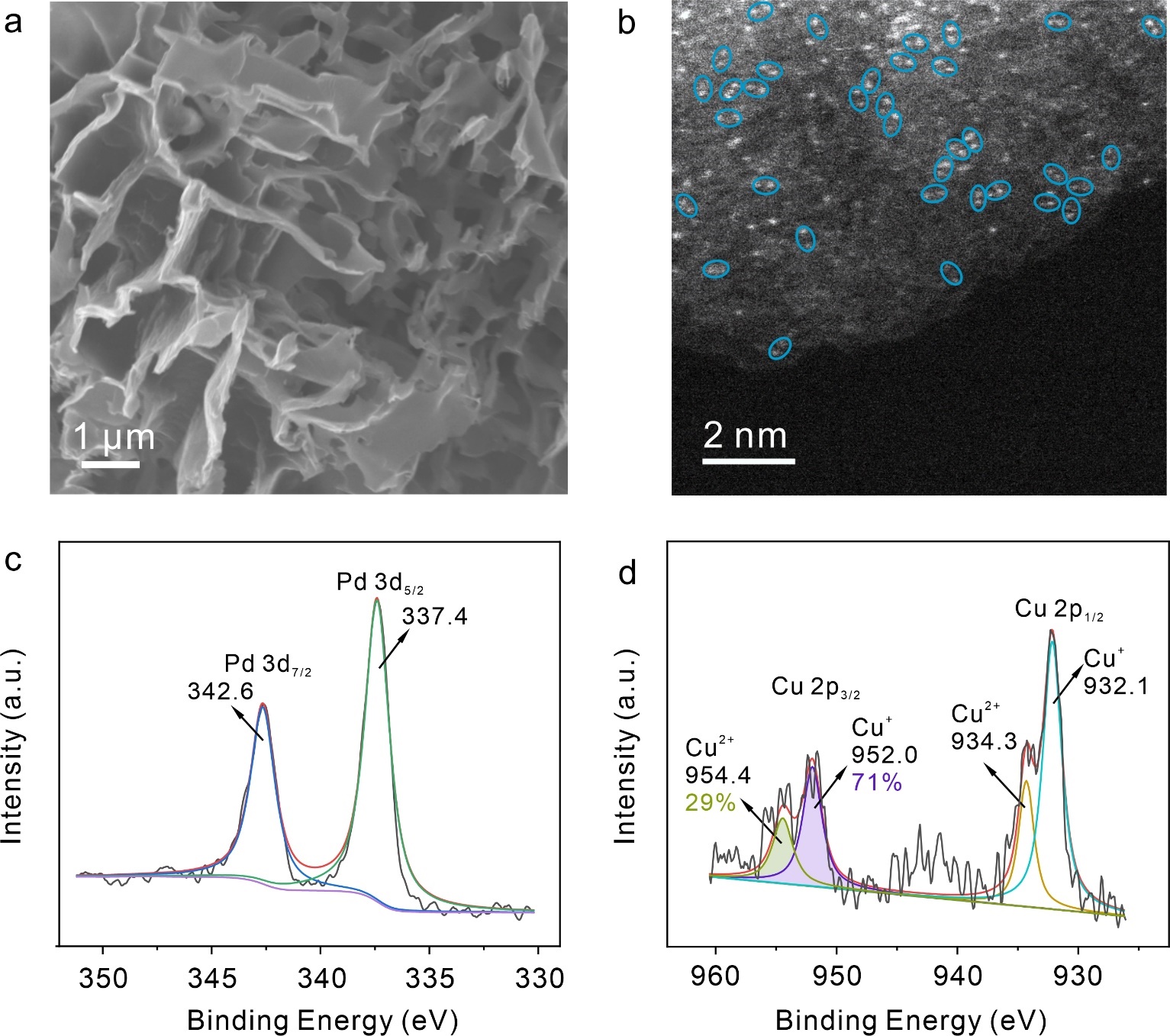


**Figure S31.** a) SEM image and b) HAADF-STEM image of the used Pd-Cu DAC after electrochemical stability tests. HR-XPS spectra of c) Pd 3d and d) Cu 2p in the used Pd-Cu DAC after electrochemical stability tests.


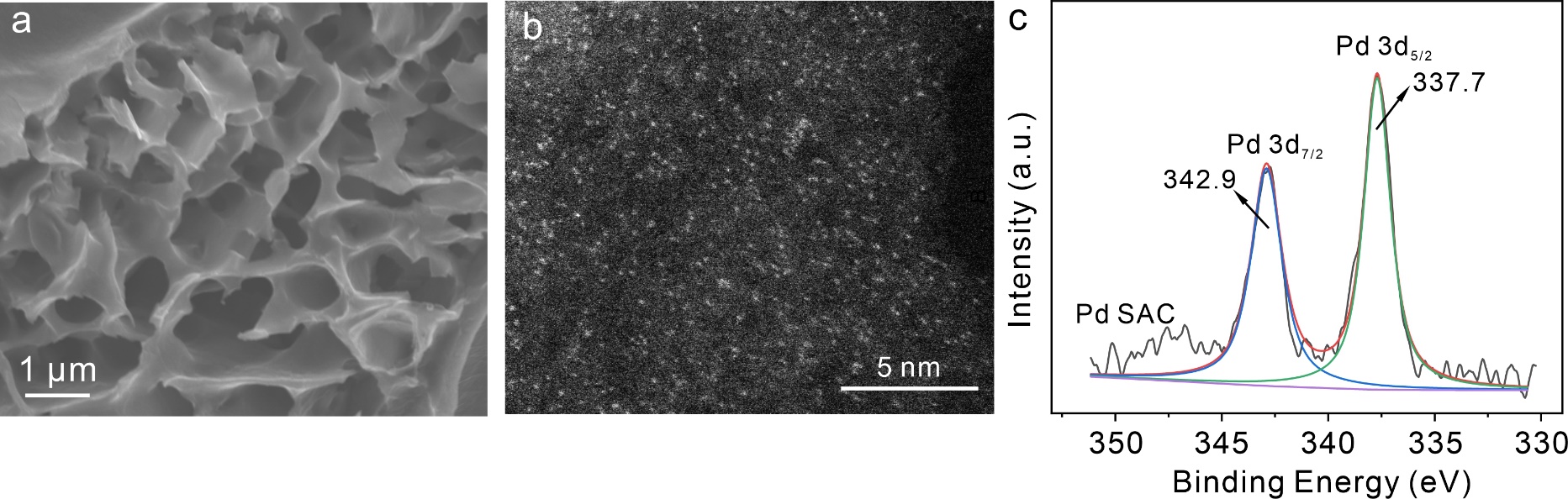


**Figure S32.** a) SEM image and b) HAADF-STEM image of the used Pd SAC after electrochemical stability tests. c) HR-XPS spectra of Pd 3d in Pd SAC after electrochemical stability tests.


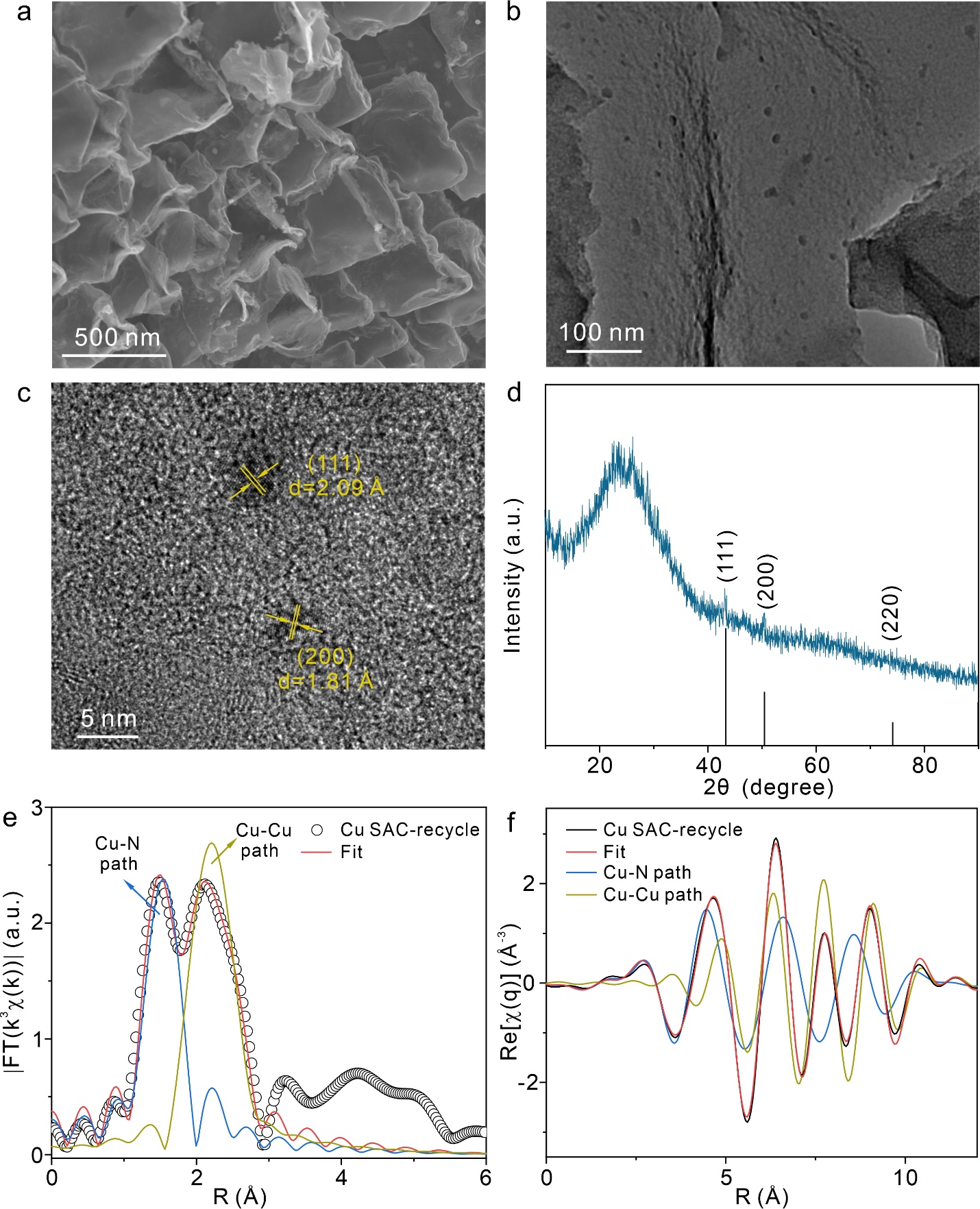


**Figure S33.** a) SEM image, b, c) TEM images, and d) XRD pattern of the used Cu SAC after electrochemical stability tests. e) Cu K-edge EXAFS spectra (R space, k^3^-weighted) of the used Cu SAC and f) the corresponding k^3^χ(k) oscillation curves.


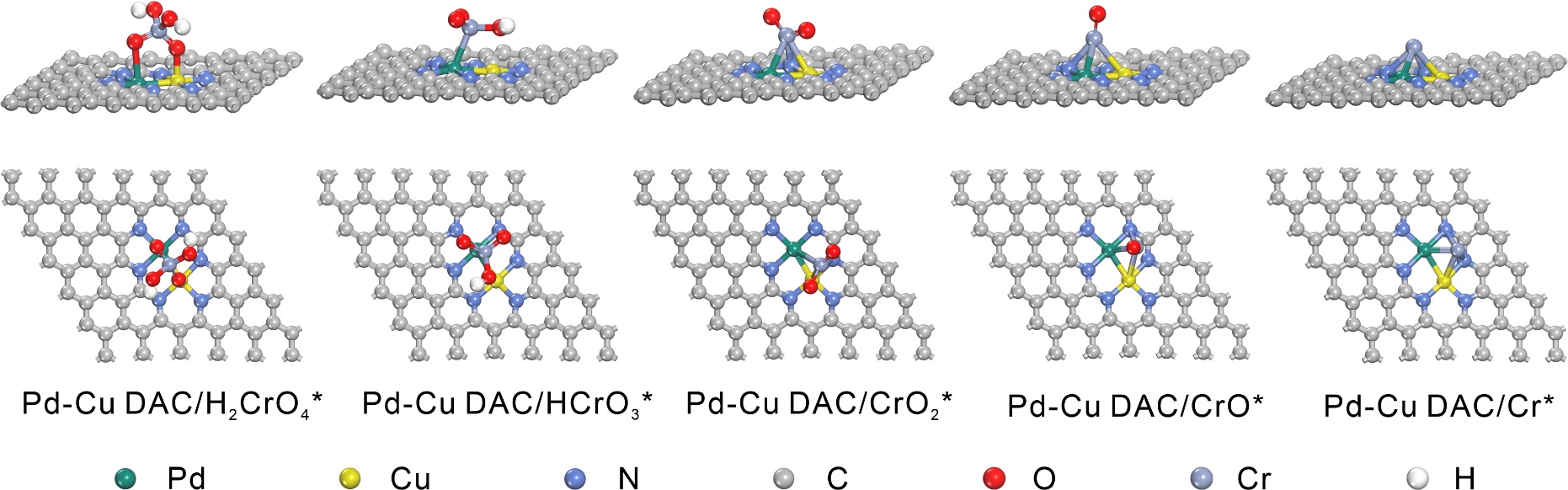


**Figure S34.** Side view and top view of the optimal interaction configuration of Pd-Cu DAC with Cr(VI)-transition states during the reduction reactions of Cr(VI) into Cr(III).


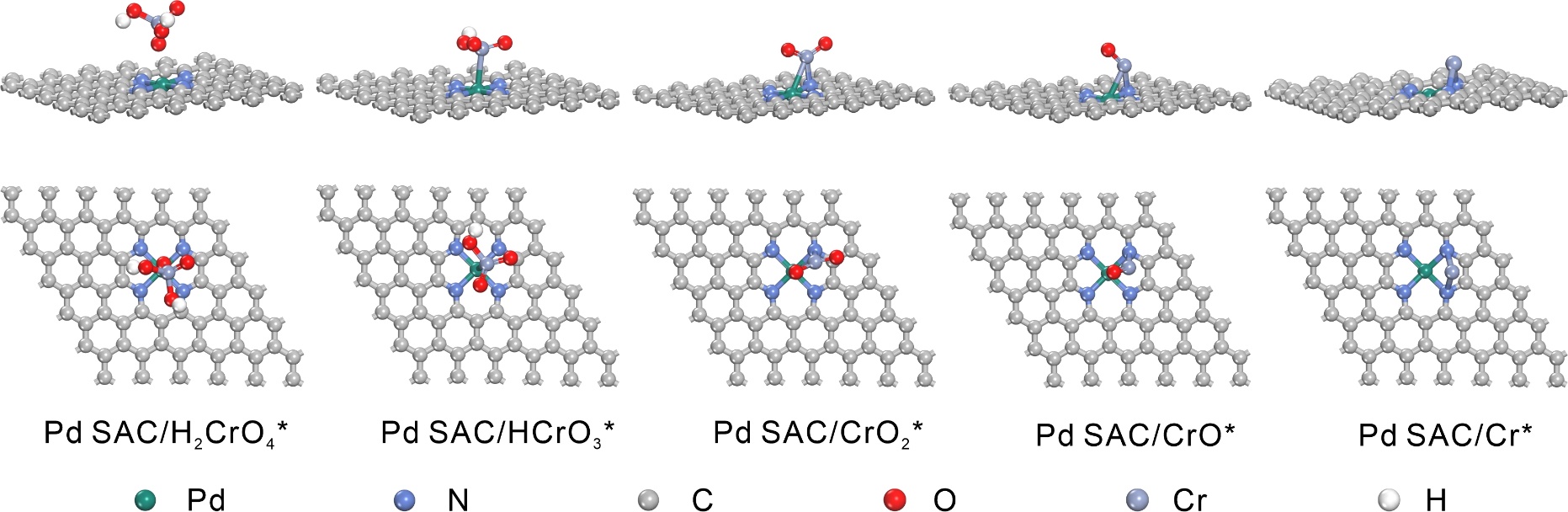


**Figure S35.** Side view and top view of the optimal interaction configuration of Pd SAC with Cr(VI)-transition states during the stepwise reduction reactions of Cr(VI) into Cr(III).


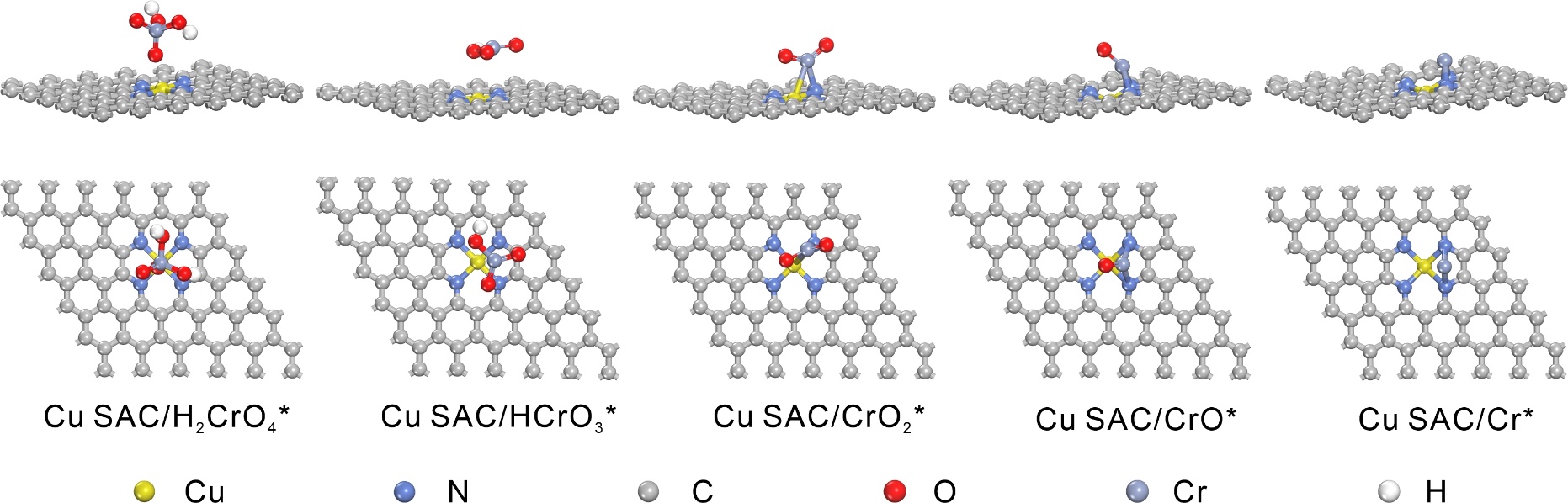


**Figure S36.** Side view and top view of the optimal interaction configuration of Pd SAC with Cr(VI)-transition states during the stepwise reduction reactions of Cr(VI) into Cr(III).

**3. Tables**

**Table S1.** The fitting results and parameters of Pd K-edge EXAFS spectra in Pd SAC and Pd-Cu DAC before and after interacting with Cr(VI) analyzed by the Artemis module of IFEFFIT (S_0_^2^=0.70).

| Samples | Path | CN | R (Å) | σ^2^ (10^-3^ Å^2^) | △E_0_ (eV) | R-factor |
| --- | --- | --- | --- | --- | --- | --- |
| Pd-Cu DAC | Pd-N | 2.9±0.3 | 2.02±0.02 | 6.9±0.2 | 10.4±2.1 | 0.010 |
|  | Pd-Cu | 0.7±0.1 | 2.44±0.03 | 6.9±0.2 | 8.5±1.4 |  |
| Pd SAC | Pd-N | 3.9±0.4 | 2.00±0.02 | 7.2±0.3 | 10.0±1.6 | 0.013 |
| Pd-Cu DAC/Cr | Pd-N/ Pd-O | 4.2±0.4 | 2.10±0.01 | 6.9±0.3 | 10.5±1.3 | 0.005 |
|  | Pd-Cu | 0.8±0.2 | 2.45±0.02 | 6.9±0.3 | 8.6±1.0 |  |
| Pd SAC/Cr | Pd-N | 3.5±0.3 | 1.98±0.03 | 7.2±0.3 | 10.0±1.1 | 0.020 |

Notes: CN, coordination number; R, the distance between absorber and backscatter atoms. σ^2^, Debye-Waller factor; △E_0_, the inner potential difference between the reference compound and the experimental sample. R-factor, goodness of fit; S_0_^2^, amplitude reduction factor.

**Table S2.** The fitting results and parameters of Cu K-edge EXAFS spectra in Cu SAC and Pd-Cu DAC before and after interacting with Cr(VI) analyzed by the Artemis module of IFEFFIT (S_0_^2^=0.85).

| Samples | Path | CN | R (Å) | σ^2^ (10^-3^ Å^2^) | △E_0_ (eV) | R-factor |
| --- | --- | --- | --- | --- | --- | --- |
| Pd-Cu DAC | Cu-N | 2.8±0.4 | 1.95±0.02 | 8.0 | 5.41±0.45 | 0.002 |
|  | Cu-Pd | 0.6±0.2 | 2.53±0.01 | 8.0 | 5.21±0.40 |  |
| Cu SAC | Cu-N | 3.7±0.2 | 1.94±0.02 | 8.0 | 5.41±0.60 | 0.008 |
| Pd-Cu DAC/Cr | Cu-N/ CuO | 4.0±0.2 | 1.97±0.01 | 8.0 | 5.45±0.52 | 0.001 |
|  | Cu-Pd | 0.7±0.1 | 2.56±0.01 | 8.0 | 5.20±0.80 |  |
| Cu SAC/Cr | Cu-N | 3.9±0.1 | 1.95±0.02 | 8.0 | 5.40±0.80 | 0.010 |

Notes: CN, coordination number; R, the distance between absorber and backscatter atoms. σ^2^, Debye-Waller factor; △E_0_, the inner potential difference between the reference compound and the experimental sample. R-factor, goodness of fit; S_0_^2^, amplitude reduction factor.

**Table S3.** Comparison in Cr(VI) reduction results achieved by the noble metal materials modified electrodes that previously reported.

| Electrodes | Electrolyte | Method | Linear range (ppb) | LOD  (ppb) | Sensitivity (μA ppb^-1^) | Ref. |
| --- | --- | --- | --- | --- | --- | --- |
| PcIrCl/GCE | 0.1 M HCl | CV | 5.2-1872 | 18.72 | 0.1805 | ^[4]^ |
| AuCs/BP-NG | 0.1 M H_2_SO_4_ | LSV | 20-240 | 3.7 | 0.414 | ^[5]^ |
| AuNPs-BDD | 0.1 M Sodium acetic buffer | CV | 10-1000 | 1.19 | 0.00375 | ^[6]^ |
| AuNPs/ITO | 0.01 M HCl | CV | 260-5200 | 104 | 0.0058 | ^[7]^ |
| g-C_3_N_4_/AgM/Nf | 0.1 M HCl | CV | 520-5200 | 0.00832 | 0.00082 | ^[8]^ |
| Ag-Au-SPCE | 0.1 M Acetic acid buffer | SWV | 50-5000 | 0.1 | 0.000232 | ^[9]^ |
| gold plated carbon based electrode | 0.3 HNO_3_ | LSV | 20-500 | 4.4 | 0.0007 | ^[10]^ |
| AuNPs/SPCE | 0.5 M HNO_3_ | LSV | 20-200 | 5.4 | 0.0011 | ^[11]^ |
| FeCo@PC-SE | 0.1 M HCl | Amperometry | 40-800 | 8 | 0.00775 | ^[12]^ |
| Co(P_4_Mo_6_O_31_)_2_ | 0.5 M H_2_SO_4_ | Amperometry | 26-19656 | 2.7 | 0.0047 | ^[13]^ |
| SPE-graphite | 0.1 M H_2_SO_4_ | LSV | 100-1000 | 19 | 0.00058 | ^[14]^ |
| Au/mpg-C_3_N_4_ | 0.1 M HCl | LSV | 100-1000 | 14 | 0.0022 | ^[15]^ |
| TiC@CN_x_ NFAs | 0.05 M HCl | Amperometry | 10.4-1253.2 | 0.208 | 0.0169 | ^[16]^ |
| Pd-Cu DAC | 0.5 M H_2_SO_4_ | LSV | 20-500 | 0.63 | 0.51 | This work |
| Pd SAC | 0.5 M H_2_SO_4_ | LSV | 100-1000 | 67.4 | 0.023 |  |
| Cu SAC | 0.5 M H_2_SO_4_ | LSV | 100-1000 | 56.8 | 0.034 |  |

Notes: PcIrCl, iridium tetra[4-o-tolylsulfanyl]chloride phthalocyanine; GCE, glass carbon electrode; AuCs/BP-NG, Au_25_ nanoclusters loaded on the nanocomposites of black phosphorus and N-doped graphene; BDD, boron-doped diamond; AuNPs: Au nanoparticles; ITO: indium tin oxide; mpg: mesoporous g-C_3_N_4_; TiC@CN_x_ NFAs, N-doped carbon coated TiC nanofiber arrays; SWV, square wave voltammetry; SPCE, screen-printed carbon electrodes

**Table S4.** The fitting results of Cr K-edge EXAFS spectra in Pd-Cu DAC/Cr, Pd SAC/Cr, and Cu SAC/Cr analyzed by the Artemis module of IFEFFIT (S_0_^2^=0.70).

| Samples | Path | CN | R (Å) | σ^2^ (10^-3^ Å^2^) | △E_0_ (eV) | R-factor |
| --- | --- | --- | --- | --- | --- | --- |
| Pd-Cu DAC/Cr | Cr-O | 4.2±0.3 | 1.97±0.01 | 2.9±0.3 | -3.78±1.5 | 0.018 |
| Pd SAC/Cr | Cr-O | 4.1±0.2 | 1.92±0.02 | 2.9±0.1 | -3.78±1.3 | 0.019 |
| Cu SAC/Cr | Cr-O | 3.9±0.3 | 1.93±0.01 | 2.9±0.4 | -3.78±1.1 | 0.020 |

Notes: CN, coordination number; R, the distance between absorber and backscatter atoms. σ^2^, Debye-Waller factor; △E_0_, the inner potential difference between the reference compound and the experimental sample. R-factor, goodness of fit; S_0_^2^, amplitude reduction factor.

**Table S5.** Comparison in Mulliken charges of Cr, O, and H atoms in H_2_CrO_4_ before and after adsorbing with Pd-Cu DAC, Pd SAC, and Cu SAC.

| Structural models | Mulliken charge (*e*) | | | | | | | | | |  |
| --- | --- | --- | --- | --- | --- | --- | --- | --- | --- | --- | --- |
|  | Cr | | O_1_ | O_2_ | O_3_ | O_4_ | H_1_ | H_2_ | total | | |
| H_2_CrO_4_ | | 1.40 | -0.39 | -0.39 | -0.78 | -0.78 | 0.47 | 0.47 | | 0 | |
| Pd-Cu DAC/Cr | | 1.40 | -0.52 | -0.55 | -0.80 | -0.80 | 0.43 | 0.44 | | -0.40 | |
| Pd SAC/Cr | | 1.40 | -0.42 | -0.42 | -0.79 | -0.79 | 0.46 | 0.46 | | -0.10 | |
| Cu SAC/Cr | | 1.42 | -0.46 | -0.42 | -0.79 | -0.79 | 0.46 | 0.45 | | -0.13 | |

**Table S6.** Comparison in Mulliken charges of Pd, Cu, N atoms in Pd-Cu DAC, Pd SAC, and Cu SAC before and after adsorbing H_2_CrO_4_.

| Structural models | Mulliken charge (*e*) | | | | | | | | | | | | | |
| --- | --- | --- | --- | --- | --- | --- | --- | --- | --- | --- | --- | --- | --- | --- |
|  | Pd | | Cu | | | N_1_ | N_2_ | | N_3_ | N_4_ | N_5_ | | N_6_ | |
| Pd-Cu DAC | | 0.92 | | 0.95 | -0.38 | | | -0.39 | -0.41 | -0.49 | | -0.51 | | -0.51 |
| Pd-Cu DAC/H_2_CrO_4_ | | 0.96 | | 0.99 | -0.36 | | | -0.39 | -0.47 | -0.47 | | -0.47 | | -0.48 |
| Pd SAC | | 1.11 | | - | -0.40 | | | -0.40 | -0.40 | -0.40 | | - | | - |
| Pd SAC/Cr | | 1.14 | | - | -0.39 | | | -0.39 | -0.39 | -0.39 | | - | | - |
| Cu SAC | | - | | 1.42 | -0.49 | | | -0.49 | -0.49 | -0.49 | | - | | - |
| Cu SAC/Cr | | - | | 1.44 | -0.48 | | | -0.48 | -0.47 | -0.47 | | - | | - |

**Table S7.** The fitting results of Cu K-edge EXAFS spectra in Pd-Cu DAC collected during the in-situ electrochemical reduction of Cr(VI) under different applied potentials. (S_0_^2^=0.85)

| Samples | Path | CN | R (Å) | σ^2^ (10^-3^ Å^2^) | △E_0_ (eV) | R-factor |
| --- | --- | --- | --- | --- | --- | --- |
| Pd-Cu DAC/Cr | Cu-N /Cu-O | 4.2±0.1 | 1.95±0.01 | 8.0 | 5.39±0.68 | 0.004 |
|  | Cu-Pd | 0.6±0.1 | 2.56±0.02 | 8.0 | 4.91±0.84 |  |
| 0.8 V | Cu-N /Cu-O | 4.2±0.2 | 1.96±0.01 | 8.0 | 5.67±0.84 | 0.004 |
|  | Cu-Pd | 0.6±0.1 | 2.55±0.02 | 8.0 | 5.42±1.05 |  |
| 0.6 V | Cu-N /Cu-O | 4.0±0.2 | 1.96±0.01 | 8.0 | 5.53±0.93 | 0.006 |
|  | Cu-Pd | 0.5±0.1 | 2.55±0.02 | 8.0 | 5.27±0.45 |  |
| 0.4 V | Cu-N /Cu-O | 4.0±0.2 | 1.96±0.01 | 8.0 | 5.60±0.86 | 0.006 |
|  | Cu-Pd | 0.5±0.2 | 2.55±0.02 | 8.0 | 5.45±0.73 |  |
| 0.2 V | Cu-N /Cu-O | 4.1±0.3 | 1.97±0.01 | 8.0 | 5.30±0.78 | 0.005 |
|  | Cu-Pd | 0.5±0.1 | 2.56±0.01 | 8.0 | 5.17±1.16 |  |
| 0.15 V | Cu-N /Cu-O | 4.1±0.2 | 1.96±0.01 | 8.0 | 5.68±0.80 | 0.004 |
|  | Cu-Pd | 0.5±0.2 | 2.56±0.01 | 8.0 | 4.86±0.26 |  |
| 0.1 V | Cu-N /Cu-O | 3.8±0.1 | 1.98±0.01 | 8.0 | 5.74±0.64 | 0.006 |
|  | Cu-Pd | 0.3±0.1 | 2.58±0.01 | 8.0 | 4.59±0.90 |  |
| 0 V | Cu-N /Cu-O | 3.4±0.2 | 1.99±0.01 | 8.0 | 5.58±0.88 | 0.009 |
|  | Cu-Pd | 0.3±0.1 | 2.59±0.01 | 8.0 | 4.64±0.66 |  |
| -0.3 V | Cu-N /Cu-O | 3.2±0.2 | 1.98±0.01 | 8.0 | 5.80±0.81 | 0.004 |
|  | Cu-Pd | 0.3±0.1 | 2.60±0.01 | 8.0 | 5.07±0.90 |  |
| -0.6 V | Cu-N /Cu-O | 3.0±0.1 | 1.99±0.01 | 8.0 | 5.50±0.46 | 0.001 |
|  | Cu-Pd | 0.3±0.1 | 2.62±0.02 | 8.0 | 4.99±1.11 |  |
| Pd-Cu/Cr-refresh | Cu-N /Cu-O | 4.2±0.2 | 1.95±0.01 | 8.0 | 5.73±0.69 | 0.003 |
|  | Cu-Pd | 0.6±0.2 | 2.56±0.01 | 8.0 | 5.02±0.76 |  |

Notes: CN, coordination number; R, the distance between absorber and backscatter atoms. σ^2^, Debye-Waller factor; △E_0_, the inner potential difference between the reference compound and the experimental sample. R-factor, goodness of fit; S_0_^2^, amplitude reduction factor.

**Table S8.** The fitting results and parameters of Cu K-edge EXAFS spectra in Cu SAC collected during the in-situ electrochemical reduction of Cr(VI) under different applied potentials. (S_0_^2^=0.85)

| Samples | Path | CN | R (Å) | σ^2^ (10^-3^ Å^2^) | △E_0_ (eV) | R-factor |
| --- | --- | --- | --- | --- | --- | --- |
| Cu SAC/Cr | Cu-N | 3.91±0.16 | 1.94±0.01 | 8.0 | 4.15±0.54 | 0.004 |
| 0.8 V | Cu-N | 3.90±0.18 | 1.94±0.01 | 8.0 | 4.38±0.60 | 0.006 |
| 0.6 V | Cu-N | 3.73±0.12 | 1.94±0.01 | 8.0 | 4.49±0.65 | 0.008 |
| 0.4 V | Cu-N | 3.74±0.10 | 1.94±0.01 | 8.0 | 4.53±0.56 | 0.006 |
| 0.2 V | Cu-N | 3.93±0.11 | 1.94±0.01 | 8.0 | 5.11±0.61 | 0.007 |
| 0.15 V | Cu-N | 2.92±0.16 | 1.94±0.01 | 8.0 | 4.27±0.91 | 0.004 |
|  | Cu-Cu | 0.47±0.13 | 2.52±0.06 | 8.0 | 18.20±1.22 |  |
| 0.1 V | Cu-N | 2.82±0.09 | 1.94±0.01 | 8.0 | 4.30±0.55 | 0.002 |
|  | Cu-Cu | 0.51±0.10 | 2.51±0.04 | 8.0 | 18.06±1.50 |  |
| 0 V | Cu-N | 2.88±0.16 | 1.94±0.01 | 8.0 | 4.43±0.96 | 0.004 |
|  | Cu-Cu | 0.50±0.13 | 2.52±0.05 | 8.0 | 18.35±1.90 |  |
| -0.3 V | Cu-N | 2.82±0.16 | 1.94±0.01 | 8.0 | 4.29±0.91 | 0.004 |
|  | Cu-Cu | 0.50±0.13 | 2.51±0.05 | 8.0 | 18.25±1.75 |  |
| -0.6 V | Cu-N | 2.86±0.13 | 1.94±0.01 | 8.0 | 4.39±0.76 | 0.002 |
|  | Cu-Cu | 0.58±0.10 | 2.52±0.05 | 8.0 | 18.54±1.58 |  |
| Cu SAC/Cr-refresh | Cu-N | 2.73±0.12 | 1.94±0.01 | 8.0 | 4.73±0.73 | 0.002 |
|  | Cu-Cu | 0.63±0.15 | 2.52±0.04 | 8.0 | 18.45±1.95 |  |
| Cu SAC-recycle | Cu-N | 1.63±0.11 | 1.97±0.01 | 8.0 | 6.77±1.48 | 0.006 |
|  | Cu-Cu | 2.56±0.38 | 2.53±0.02 | 8.0 | 10.30±0.56 |  |

Notes: CN, coordination number; R, the distance between absorber and backscatter atoms. σ^2^, Debye-Waller factor; △E_0_, the inner potential difference between the reference compound and the experimental sample. R-factor, goodness of fit; S_0_^2^, amplitude reduction factor.

**4. References**

[1] P. H. Li, Z. Y. Song, X. Y. Xiao, B. Liang, M. Yang, S. H. Chen, W. Q. Liu, X. J. Huang, *J. Hazard. Mater.* **2023**, *442*, 130122.

[2] a) J. Han, H. Bao, J. Q. Wang, L. Zheng, S. Sun, Z. L. Wang, C. Sun, *Appl. Catal. B- Environ.* **2021**, *280*, 119411; b) W. Y. Noh, E. M. Kim, K. Y. Kim, J. H. Kim, H. Y. Jeong, P. Sharma, G. Lee, J.-W. Jang, S. H. Joo, J. S. Lee, *J. Mater. Chem. A* **2020**, *8*, 18891-18902.

[3] a) X. Li, C. S. Cao, S. F. Hung, Y. R. Lu, W. Cai, A. I. Rykov, S. Miao, S. Xi, H. Yang, Z. Hu, J. Wang, J. Zhao, E. E. Alp, W. Xu, T. S. Chan, H. Chen, Q. Xiong, H. Xiao, Y. Huang, J. Li, T. Zhang, B. Liu, *Chem* **2020**, *6*, 3440-3454; b) Z. Huang, H. Pan, W. Yang, H. Zhou, N. Gao, C. Fu, S. Li, H. Li, Y. Kuang, *ACS Nano* **2018**, *12*, 208-216; c) Y. Wang, Q. Li, L. C. Zhang, Y. Wu, H. Chen, T. Li, M. Xu, S. J. Bao, *J. Mater. Chem. A* **2021**, *9*, 7137-7142.

[4] S. Aralekallu, M. Palanna, S. Hadimani, C. P. K. Prabhu, V. A. Sajjan, M. O. Thotiyl, L. K. Sannegowda, *Dalton Trans.* **2020**, *49*, 15061-15071.

[5] X. Y. Xiao, Z. Y. Song, H. Xie, Y. H. Zhao, S. H. Chen, Y. Y. Li, M. Yang, P. H. Li, H. Ji, X. J. Huang, *Adv. Funct. Mater.* **2022**, *32*, 2209283.

[6] Y. Xu, C. Xiong, C. Gao, Y. Li, C. Bian, S. Xia, *Micromachines-Basel* **2020**, *11*.

[7] M.-C. Tsai, P.-Y. Chen, *Talanta* **2008**, *76*, 533-539.

[8] A. Karthika, S. Nikhil, A. Suganthi, M. Rajarajan, *Adv. Powder Technol.* **2020**, *31*, 1879-1890.

[9] K. Zhao, L. Ge, T. I. Wong, X. Zhou, G. Lisak, *Chemosphere* **2021**, *281*, 130880.

[10] R. T. Kachoosangi, R. G. Compton, *Sens. Actuators B Chem.* **2013**, *178*, 555-562.

[11] J. Tu, Y. Gan, T. Liang, H. Wan, P. Wang, *Sens. Actuators B Chem.* **2018**, *272*, 582-588.

[12] A. Bhadra, A. Kundu, C. Retna Raj, *J. Chem. Sci.* **2021**, *133*, 125.

[13] Y. Wang, Y. Ma, Q. Zhao, L. Hou, Z. Han, *Sens. Actuators B Chem.* **2020**, *305*, 127469.

[14] P. M. Hallam, D. K. Kampouris, R. O. Kadara, C. E. Banks, *Analyst* **2010**, *135*, 1947-1952.

[15] X. Chen, X. X. Ke, Y. Liu, R. Weerasooriya, H. Li, Y. C. Wu, *J. Environ. Chem. Eng.* **2021**, *9*, 104642.

[16] J. Yang, H. Qi, A. Li, X. Liu, X. Yang, S. Zhang, Q. Zhao, Q. Jiang, Y. Su, L. Zhang, J. F. Li, Z. Q. Tian, W. Liu, A. Wang, T. Zhang, *J. Am. Chem. Soc.* **2022**, *144*, 12062-12071.
